# MYOD1 mutation drives cancer stem cell pathways and therapy-resistance in spindle cell/sclerosing rhabdomyosarcoma

**DOI:** 10.1038/s41467-026-73546-7

**Published:** 2026-06-03

**Authors:** Yun Wei, Luis Antonio Corchete Sánchez, Sabateeshan Mathavarajah, Diego Antelo, Shuze Wang, Jihee Lee, Alexander Daiki Weissman, Devika D. Kannambadi, Qian Qin, Sara G. Danielli, Elisa J. Quantin, Tiffany C. Eng, Alexandra Veloso, Yueyang Wang, Gunnlaugur P. Neilsen, Chuan Yan, Valerie Shiwen Yang, Anand G. Patel, Selene C. Koo, Patience Odeniyide, Christine A. Pratilas, Miguel N. Rivera, Esther Rheinbay, David M. Langenau

**Affiliations:** 1 Molecular Pathology Unit, Massachusetts General Research Institute, Harvard Medical School, Charlestown, MA USA; 2 Krantz Family Center for Cancer Research, Massachusetts General Hospital, Charlestown, MA USA; 3 Harvard Stem Cell Institute, Harvard Medical School, Cambridge, MA USA; 4 Broad Institute of MIT and Harvard, Cambridge, MA USA; 5 Department of Pediatric Oncology, Dana-Farber Boston Children’s Cancer and Blood Disorders Center, Boston, MA USA; 6 Center for Sarcoma and Connective Tissue Oncology, Department of Orthopedic Surgery, Massachusetts General Hospital, Boston, MA USA; 7 Institute of Molecular and Cell Biology (IMCB), Agency for Science, Technology and Research (A*STAR), Singapore, Singapore; 8 Division of Medical Oncology, National Cancer Centre Singapore, Singapore, Singapore; 9 Oncology Academic Clinical Program, Duke-NUS Medical School, Singapore, Singapore; 10 Department of Developmental Neurobiology, St. Jude Children’s Research Hospital, Memphis, TN USA; 11 Department of Oncology, St. Jude Children’s Research Hospital, Memphis, TN USA; 12 Department of Pathology, St. Jude Children’s Research Hospital, Memphis, TN USA; 13 Division of Pediatric Oncology, The Sidney Kimmel Comprehensive Cancer Center, Johns Hopkins University School of Medicine, Baltimore, MD USA

**Keywords:** Cancer genetics, Cancer stem cells, Sarcoma

## Abstract

Tumor growth and relapse are often driven by cancer stem cells, but self-renewal mechanisms and genetic mutations that elevate their number are not fully understood. Here, we show that recurrent L122R mutation (Leucine to Argine change at amino acid 122) in the DNA-binding site of the Myogenic Differentiation 1 (MYOD1) transcription factor increases cancer stem cell frequency in aggressive Spindle cell/sclerosing rhabdomyosarcoma. MYOD1^L122R^ also makes tumors resistant to chemotherapy and radiation. Epigenetic analysis reveals that MYOD1^L122R^ binds MYC-like DNA recognition motifs to activate stem cell programs, while retaining some wild-type function to regulate muscle pathways that drive transformation. Mechanistically, MYOD1^L122R^ transcriptionally activates *Receptor tyrosine kinase-like Orphan Receptor 2 (ROR2)* to turn on the non-canonical Wingless/Integrated (WNT) planar cell polarity pathway to increase both cancer stemness and therapy resistance. Targeting ROR2 with antibody-drug conjugates kills MYOD1^L122R^-mutated tumor cells, offering therapeutic opportunities. These findings provide insights into how MYOD1^L122R^ rewires rhabdomyosarcoma to a stem-like state and defines a unique class of oncogenic transcription factors found in aggressive cancers.

## Abstract (web summary)

A molecular alteration, MYOD1^L122R^, predicts poor prognosis for patients with rhabdomyosarcoma. Here, the authors demonstrate that this mutation increases cancer stem cell frequency via upregulation of ROR2, identifying a therapeutic target in preclinical models of MYOD1^L122R^ rhabdomyosarcoma.

## Introduction

Cancer stem cells (CSCs) are critical drivers of tumor growth and relapse, as they possess the unique ability to self-renew, generate heterogeneous tumor progeny and propagate cancer^1^. This self-renewal capacity is often governed by the same signaling pathways found in normal development and regeneration after injury, including Wnt, Planar Cell Polarity, Notch, and Hedgehog pathway activation^2–6^. In tumors that follow the cancer stem cell model, these tumor-propagating CSCs are thought to form a reservoir of resistant cells, which can evade conventional therapies like chemotherapy and radiation, contributing to tumor recurrence after initial treatment. Despite significant advancements in identifying CSCs in a subset of malignancies, much remains unknown about the specific pathways that regulate their retention and expansion within tumors, hindering the development of effective therapies targeting this population, especially in the context of pediatric cancers. Additionally, CSCs can exhibit considerable heterogeneity in pathways that drive elevated stemness even within related tumors, making it difficult to identify common self-renewal mechanisms and to develop strategies for CSC eradication. Unraveling the complexities of CSC biology will be critical for developing more targeted and effective cancer treatments.

Cancer is highly heterogeneous, and this is true even within malignancies that develop from similar cells and tissue-of-origin. For example, rhabdomyosarcoma (RMS) is a common skeletal muscle cancer found in children and adolescents where tumor cells are arrested in an undifferentiated muscle cell state and yet contain distinct and transcriptionally-defined cell states that differ between subtypes^7–11^. Using single cell RNA sequencing and functional experiments, we and others have shown that RMS without characteristic *PAX3/7-FOXO1* gene fusions (fusion-negative; FN-RMS) have RAS pathway activation, are hierarchically organized, and contain a transcriptionally unique cancer stem cell population^8,11^. By contrast, fusion-positive RMS (FP-RMS) have chromosome translocations resulting in the genesis of the fusion-oncoproteins PAX3-FOXO1 or PAX7-FOXO1, contain different cell populations that include neural cells, and likely do not follow a cancer stem cell model^12–14^. In 2014, the WHO identified a third subtype of pediatric and adolescent rhabdomyosarcoma called spindle cell/sclerosing rhabdomyosarcoma (SS-RMS)^15–19^. Genome sequencing identified that a large fraction of SS-RMS harbor a recurrent somatic mutation within the DNA binding motif of the MYOD1 transcription factor (TF; leucine residue 122 to arginine, L122R). These MYOD1^L122R^ mutated SS-RMS are the most aggressive, therapy resistant, and nearly universally fatal subtype of RMS^15,16,20^. Yet, apart from its association with inferior outcome, it is unknown how MYOD1^L122R^ promotes tumor aggression, especially in the context of cancer stem cell biology and therapy resistance. In addition, detailed mechanistic studies have yet to be completed to show how this gene mutation alters RMS biology or how these tumors might be therapeutically targeted.

In this work, we show that MYOD1^L122R^ elevates the number of tumor-propagating cancer stem cells, while simultaneously increasing resistance to chemotherapy and irradiation. Genome-wide binding studies reveal that MYOD1^L122R^ alters DNA binding specificity to recognize the MYC:MAX motif and transcriptionally upregulates SS-RMS cancer stem cell programs. Yet, MYOD1^L122R^ also retains a subset of canonical binding targets to lock cells in a transformed skeletal muscle state. Specifically, *Receptor tyrosine kinase-like Orphan Receptor 2 (ROR2)* is a direct and unique transcriptional target of MYOD1^L122R^. Following transcriptional upregulation of *ROR2*, it activates a non-canonical planar cell polarity WNT11-ROR2-VANGL2-RHOA pathway. Finally, we use the ROR2 antibody drug conjugate, Ozuriftamab vedotin, to target ROR2-expressing MYOD1^L122R^ RMS, providing therapeutic opportunities to explore in future. Our work also identified that DNA binding domain mutations in developmental transcription factors likely define aggressive subtypes of cancers, with a common theme of these mutations resulting in gain-of-function neomorphic DNA binding site specificity while also retaining wild-type function to drive transformation.

## Results

### MYOD1^L122R^ increases tumor-propagating cell number in RAS-pathway-activated zebrafish RMS

**Fig. 1:**
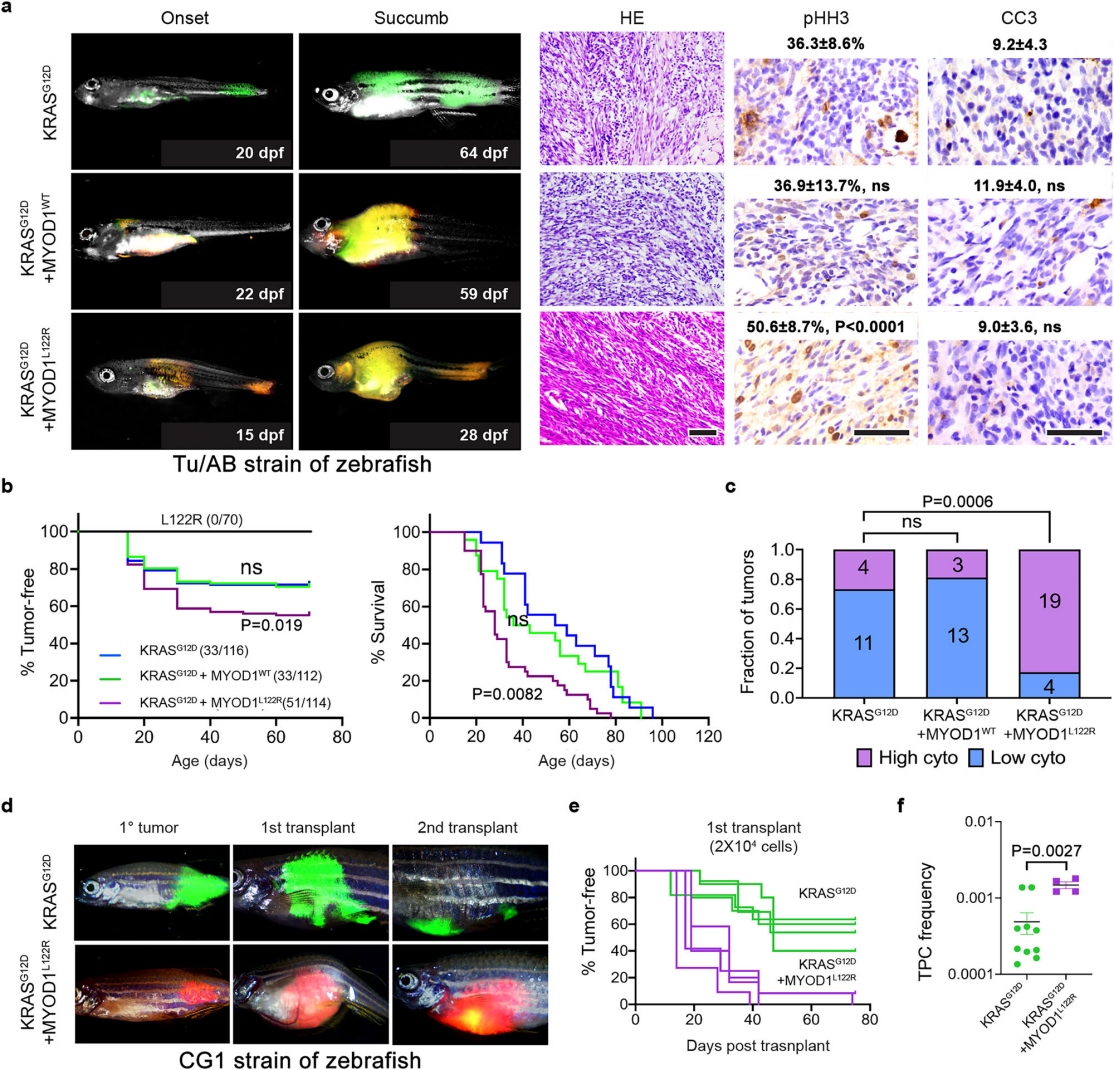
*MYOD1*^L122R^ collaborates with *KRAS*^G12D^ to increase RMS aggressiveness and to elevate cancer stemness in zebrafish RMS. **a** Representative images of Tu/AB transgenic zebrafish shown at the time of tumor onset or when moribund at sacrifice (left two panels, merged brightfield and fluorescent images) and juxtaposed with tumor histology (right image panels). Hematoxylin and eosin (HE), proliferation marker phosphorylated histone 3 (pHH3) (numbers indicate percentage of total population), and apoptosis marker cleaved caspase 3 (CC3) (numbers indicate cells per field of view). Days post-fertilization (dpf) noted within images. Scale bars equal 50 µm. Quantification denotes number of positive cells (mean ± std, with asterisks denoting statistical differences in comparison to KRAS^G12D^ alone expressing RMS, two-sided Student’s *t*-test, right, see Source Data file for detailed numbers of fish). **b** Tumor onset and tumor survival curves. The number of fish that were microinjected with *rag2* promoter driven transgenes and developed RMS, compared to the total number of fish analyzed are note in parentheses, Logrank (Mantel-Cox) test was used for onset and survival analysis. **c** Quantification of the number of animals with defined tumor histology. High and low cytoplasmic (cyto) to nuclei ratio, Two-sided chi-square was used for two comparisons as indicated, ns., non-significant. **d–f** Analysis of tumor growth in CG1 strain zebrafish models. Representative images of zebrafish with primary tumors (1°), first transplant (1st), and secondary transplant (2nd). Zebrafish were engrafted at ~2 months of age. Merged brightfield and fluorescent images shown (**d**). Kaplan–Meier analysis depicting tumor onset in primary transplanted fish (**e**, *n* = 6–8 recipient fish/tumor, 2 × 10^4^ cells injected per fish). Tumor-propagating cell frequency calculated based on secondary transplant using limiting dilution cell transplantation and ELDA analysis (**f**, KRAS^G12D^ (*n* = 10 primary fish tumors, including 6 previously published samples^32,47^), KRAS^G12D^ + MYOD1^L122R^ (*n* = 4 primary fish tumors), mean ± S.E.M noted), two-sided Student’s *t*-test was performed. ns, non-significant, for (**a**–**c**). *P* < 0.05 was considered statistically significant. Source data are provided as a Source data file.

To investigate the role of MYOD1^L122R^ in RMS onset and aggression, we transgenically expressed human *MYOD1*^L122R^ under control of muscle-expressing gene promoters, including m-*cadherin (cdh15), myogenin (myog), myosin light chain, phosphorylatable, fast skeletal muscle (mylpfa),* and *recombinase activating gene 2 (rag2; promoter:MYOD1*^L122R^-T2A-*tdTomato*)^21^. Specifically, wildtype Tuebingen/AB (Tu/AB) zebrafish were microinjected at the one-cell stage and then followed weekly for tumor onset for up to 2 years (Fig. 1b, Supplementary Fig. S1a, and Supplementary Table 1). Remarkably, no zebrafish developed RMS when transgenically expressing only *MYOD1*^L122R^, irrespective of the promoter used (*n* = 0 of 243). These results suggest that *MYOD1*^L122R^ is not oncogenic on its own, but rather is likely a modifying gene associated with RMS aggression.

*MYOD1*^L122R^ mutation frequently co-occurs with activating point mutations in *RAS* family members in an aggressive subtype of human SS-RMS^15,22,23^. To experimentally test if RAS pathway activation collaborates with MYOD1^L122R^ to alter tumor onset and/or to elevate overall aggression, we co-injected *rag2-KRAS*^G12D^ and *rag2-GFP* into one-cell stage fish alone or in combination with *rag2-MYOD1-T2A-tdTomato* or *rag2-MYOD1*^L122R^*-T2A-tdTomato*. Animals that co-expressed mutant *MYOD1*^L122R^ and *KRAS*^G12D^ had higher disease penetrance and worse survival when compared to *KRAS*^G12D^-alone or *KRAS*^G12D^ + *MYOD1* expressing tumors (Fig. 1a, b, Supplementary Fig. S1b, and Supplementary Table 2), suggesting that RAS pathway activation is necessary for MYOD1 oncogenic function. Next, we assessed the histology of tumors and found that *MYOD1*^L122R^-expressing tumors had larger numbers of spindled cells and contained cells with a higher cytoplasm-to-nuclei ratio, a phenotype that is commonly associated with mesenchymal cells (*n* = 19 of 23 in MYOD1^L122R^ tumors had spindle morphology compared with *n* = 4 of 15 in KRAS^G12D^-alone expressing tumors, *p* < 0.001 Fisher’s Exact test, Fig. 1a, c). In addition, we observed higher numbers of proliferative cells in *MYOD1*^L122R^ expressing tumors as determined by phosphorylated Histone 3 (pHH3) staining with no overall changes in apoptosis when assessed by activated cleaved caspase 3 staining (CC3, Fig. 1a).

We next analyzed the aggression of transplanted tumors, comparing engraftment growth and penetrance when injected into CG1-strain, adult syngeneic zebrafish. As may be expected based on the elevated aggressiveness of primary tumors (Supplementary Fig. S1b), we found that transplantation of 2 × 10^4^ *KRAS*^G12D^ + *MYOD1*^L122R^ expressing RMS cells led to fully penetrant disease, while only ~50% of *KRAS*^G12D^-alone expressing RMS developed tumors (Fig. 1d, e). Finally, we transplanted sorted tumor cells into syngeneic CG1-strain recipient zebrafish at limiting dilution to assess effects on tumor-propagating cell (TPC) frequency (sorting strategy shown in Supplementary Fig. S1c–f). Indeed, we observed a significant three-fold increase in TPCs from *MYOD1*^L122R^ tumors when compared to *KRAS*^G12D^ alone expressing RMS (*p* < 0.0027; Fig. 1f), indicating that *MYOD1*^L122R^ is a modifier of disease aggression and acts specifically by increasing tumor stemness.

### *MYOD1*^L122R^ induces cancer stemness and drives therapy resistance in human RMS

To study the gain-of-function effects mediated by MYOD1^L122R^, we conditionally expressed HA-tagged MYOD1^WT^ or HA-tagged mutant MYOD1^L122R^ in fusion-negative human RMS cell lines, RD and Ruch2. RD endogenously expresses wildtype MYOD1 while Ruch2 does not, and both have mutational activation of the RAS signaling pathway (Supplementary Fig. S2a, b). In addition, we used CRISPR homology-directed-repair (HDR) followed by single cell cloning^24^ to generate RD cell models that harbored (i) a single allele knock-in of the missense T > G DNA change that results in the L122R mutation (clones LR1 and LR2), (ii) deletion of one copy of *MYOD1* to account for possible effects of single copy loss-of-function (clones DE1 and DE2), or (iii) two wild-type copies of *MYOD1*, generated from CRISPR clones that lacked mutation of the *MYOD1* locus when assessed by PCR Sanger sequencing and Western blot (clones WT1 and WT2, Supplementary Fig. S2c). Growth rates were similar between parental and engineered RMS models (Supplementary Fig. S2d-f).

**Fig. 2:**
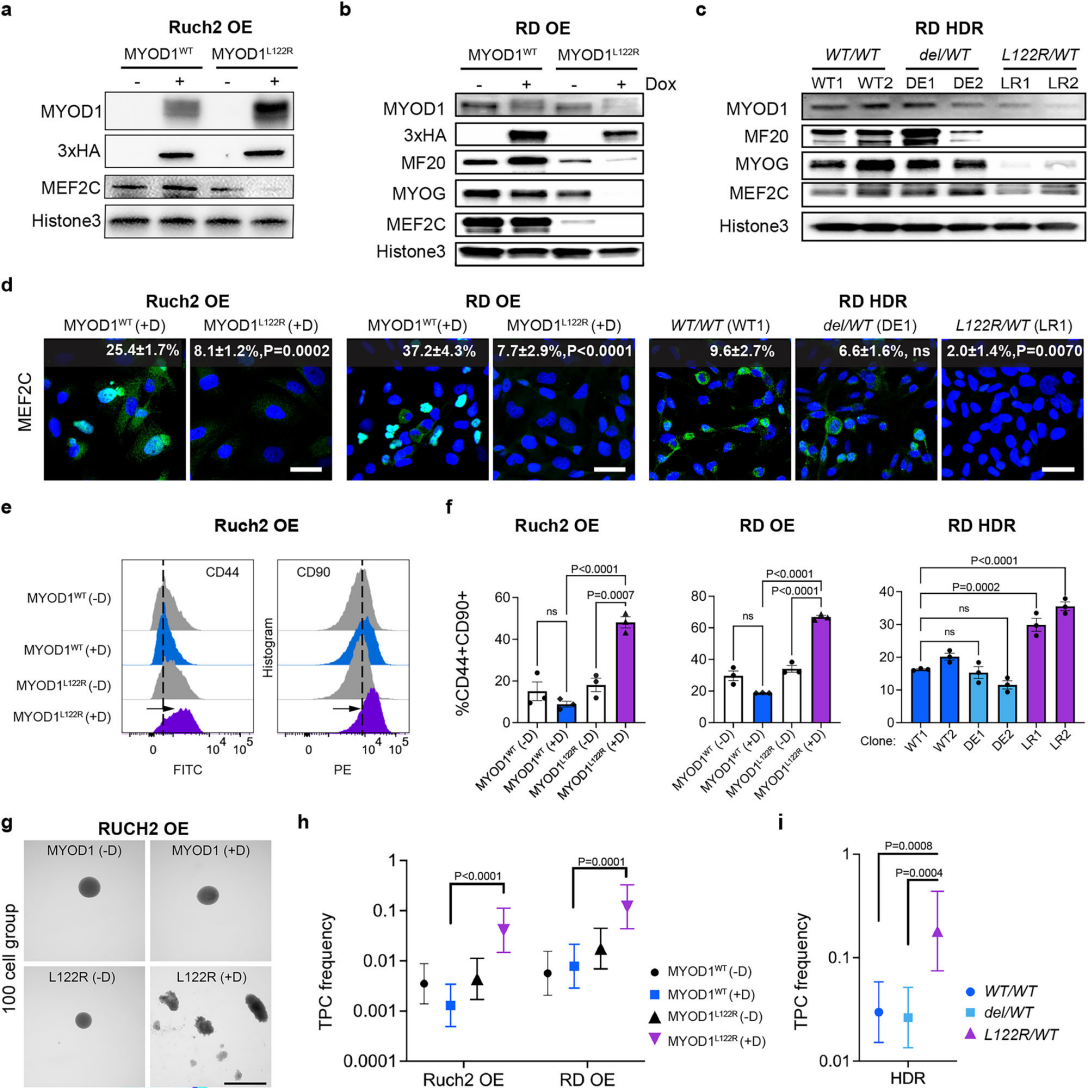
*MYOD1*^L122R^ suppresses muscle differentiation and elevates cancer stemness in human engineered, isogeneic RMS models. **a–c** Western blot analysis of doxycycline-inducible (over expression, OE) or CRISPR/Cas9 knock-in models generated by homology-directed-repair (HDR) that express MYOD1 and/or MYOD1^L122R^. Addition of doxycycline (Dox) noted by + signs in (**a**, **b**). Representative blots shown for three biological replicates with similar results. **d** Immunofluorescence images and quantification of the numbers of differentiated MEF2C+ cells (green) and counterstained with DAPI nuclei stain (blue) in doxycycline-induced models (+D) or representative HDR clones, scale bar equals 20 µm. Data obtained from individual fields of view for each condition (*n* = 3 biological replicates). Experiment was replicated independently twice with similar results. **e**, **f** Flow cytometry analysis for stemness markers CD44 and CD90 (**e)** and quantification of percent CD44 + CD90+ progenitor cancer stem cells within each model (**f**). Data obtained from three independent biological replicates (**f**, mean ± S.E.M. noted). Experiment was replicated independently twice with similar results. **g–i** Tumorsphere assays to determine the tumor-propagating cell (TPC) frequency in engineered RMS models. Representative images of tumorspheres from Ruch2 OE cells (**g)** and quantification of TPC frequency by limiting dilution tumorsphere assay and ELDA analysis (**h**, **i**, markers note median and whisker plot lines ± 95% confidence interval, with *p* values indicated in Source Data), scale bar equals 100 µm (**g**). Tumor sphere assay was replicated independently twice with similar results (*n* = 3 biological replicates for RD OE and RUCH2 OE; *n* = 6 biological replicates for RD HDR). For statistical comparison, data were analyzed using a One-way ANOVA followed by Tukey’s multiple-comparison (**d**, **f**, **h**, **i**). The samples derive from the same experiment but run on different gels for MYOD1, 3xHA, MEF2C, Histone 3, and MF20, with the exception of MYOG and MF20 (for **b**) that were run on the same gel. All samples were processed in parallel (**a**–**c**). *P*  <  0.05 was considered statistically significant. Source data are provided as a Source data file.

To assess the role of MYOD1^L122R^ in suppressing muscle differentiation in human RMS, we examined expression of myocyte-specific enhancer factor 2C (MEF2C), myogenin (MYOG), and myosin heavy chain (MF20). MYOD1^L122R^ RMS cells had low to no expression of MF20, MYOG, or MEF2C when assessed by Western blot (Fig. 2a-c) and had significantly reduced numbers of MEF2C- and MYOG-expressing cells by immunofluorescence staining (*p* < 0.01 for all comparisons, Fig. 2d and Supplementary Fig. S3a–e). We also observed a significant two-fold increase in CD44 + CD90+ expressing cells^8^ by flow cytometry in MYOD1^L122R^-engineered models (*p* < 0.05, Fig. 2e, f and Supplementary Fig. S3f), suggesting that MYOD1^L122R^ confers stem-like properties to the tested cell lines. MYOD1^L122R^ models also had elevated numbers of ALDH positive cells (Aldefluor assay, Supplementary Fig. S3g, h), a well-known marker of cancer stem cells in breast cancer^25^ and was recently shown to enrich for TPCs in FN-RMS^26^. We also confirmed elevated tumor-propagating potential and higher stemness using limiting-dilution tumorsphere assays in the *MYOD1*^L122R^ engineered models, with each showing a remarkable ten-fold increase in the overall numbers of tumor-propagating cells (*p* < 0.001, Fig. 2g, h and Supplementary Fig. S3i–k). MYOD1^L122R^ RMS cells also failed to differentiate when grown in low serum medium conditions and retained elevated stemness as assessed by the high overall numbers of CD44+ CD90+ cells (Supplementary Fig. S4). Finally, we validated that PDX models with *MYOD1*^L122R^ also have significantly reduced numbers of differentiated cells by measuring Myogenin and Desmin, well-established muscle differentiation markers^11^ (*p* < 0.0001, Supplementary Fig. S5). Taken together, these data demonstrate a major role for MYOD1^L122R^ in inducing cancer stemness and preventing differentiation.

**Fig. 3:**
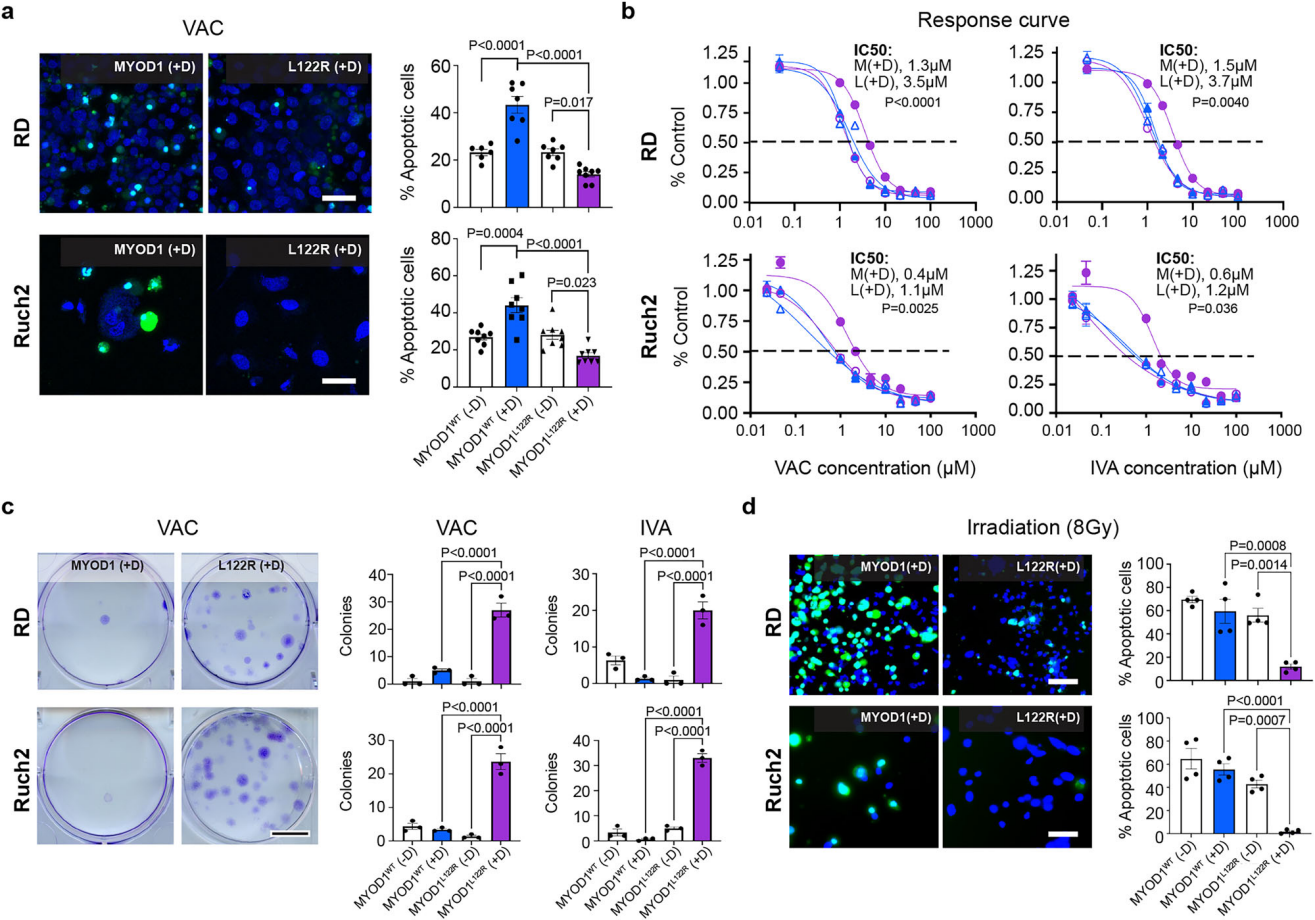
*MYOD1*^L122R^ induces chemotherapy- and irradiation-resistance. **a** Representative confocal images of engineered cell models treated 4.8 µM VAC (vincristine, actinomycin-D and cyclophosphamide) and imaged at 48 h with the apoptotic reporter Cell Event Caspase-3/7 Green (green) and DAPI nuclei stain (blue, left panels) with quantification noted to right. Doxycycline-inducible MYOD1 models and MYOD1^L122R^ expressing models shown. Doxycycline addition or not noted by + or −. Scale bar equals 20 µm. mean ± S.E.M. Data obtained from individual fields of view for each condition across multiple wells (*n* = 8 biological reps). Experiment was replicated independently twice with similar results. **b** Dose-response curves for engineered models treated with VAC or IVA (ifosfamide, vincristine, and actinomycin D). IC_50_ noted for doxycycline treated cells expressing MYOD1 and MYOD1^L122R^. Statistical significance was determined using a Two-Way ANOVA followed by Šídák’s multiple comparisons test comparing doxycycline-treated MYOD1 and MYOD1^L122R^ cells. Indicated significance for multiple comparison (between WT and L122R) refers to dosage at 2.1 µM (next highest concentration after IC50 for WT for RD and RUCH2). Data are mean ± STD from *n*  =   3 biological replicates. Experiment was replicated independently twice with similar results. **c** Analysis of clonogenic tumor growth following treatment with 4.8 µM VAC or IVA for 48 h and then plated at 200–300 cells/well in media. Representative images of crystal-violet staining of RMS cells after 14 days of drug removal with quantification noted to the right, scale bar equals 1 cm. mean ± S.E.M. Shown is a representative example from one of three independent, biological replicate experiments; similar results were replicated in an independent experiment. **d** Representative confocal images of engineered cell models treated with irradiation (8 Gy, Cs-137) and imaged after 5 days with the apoptotic reporter Cell Event Caspase-3/7 Green (green) and DAPI nuclei stain (blue, left panels), with quantification noted to right, scale bar equals 20 µm, mean ± S.E.M. Shown is a representative example from one of four biological replicates; similar results were observed three times in independent experiments. One-way ANOVA followed by Tukey’s multiple comparison, in **a**, **c**, **d**. *P* < 0.05 was considered statistically significant. Source data are provided as a Source data file.

To test if *MYOD1*^L122R^ tumor models have reduced responses to chemotherapy or radiation, we next exposed our engineered RMS models to two standard-of-care chemotherapies: VAC (vincristine, actinomycin D, and cyclophosphamide) and IVA (I-ifosfamide, vincristine, and actinomycin D)^27^. MYOD1^L122R^ engineered RMS models had significantly fewer caspase-3/7+ apoptotic cells following VAC or IVA treatment when compared to parental cells or cells that express wild-type MYOD1 (*p* < 0.001, Fig. 3a and Supplementary Fig. S6a, b). Moreover, the dose response curves for both VAC and IVA were right shifted, reflecting that 2-3-fold higher drug concentrations were required to induce apoptosis in MYOD1^L122R^ tumor cells over the 48-hour experiment (Fig. 3b). RMS with *MYOD1*^L122R^ also regrew more efficiently after VAC and IVA treatment, forming greater than 8 times more colonies compared to control cells in clonogenic assays two weeks post-drug treatment (*p* < 0.001, Fig. 3c and Supplementary Fig. S6c). Similarly, MYOD1^L122R^ expressing cells had fewer caspase-3/7+ cells 5 days following 8 Gy radiation (Fig. 3d) and efficiently regrew significantly more colonies 3 weeks after radiation (Supplementary Fig. S6d). Taken together, MYOD1^L122R^ confers resistance to commonly used cancer therapy.

**Fig. 4:**
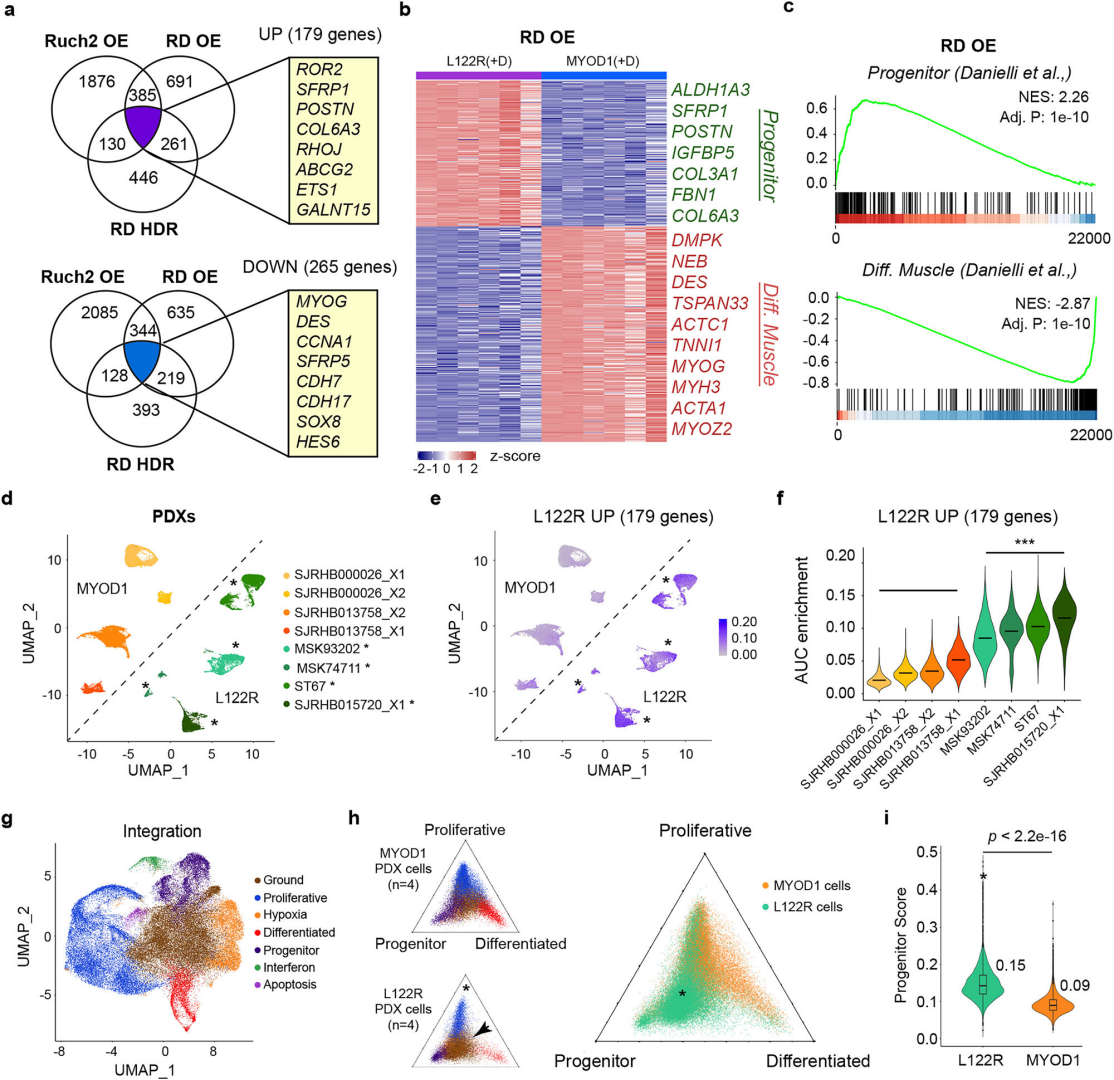
MYOD1^L122R^ transcriptionally upregulates progenitor stem cell programs while suppressing differentiation in engineered and patient-derived SS-RMS models. **a**, **b** Bulk RNA-sequencing identified genes that were up- and down-regulated in comparing MYOD1^L122R^ engineered models. Venn diagram visualization identifies high-confidence transcriptional targets that were found to be similarly regulated in three models (179 upregulated and 265 down regulated genes, (**a**)). Heatmap showing gene expression in doxycycline-inducible RD OE models with z-score indicated in (**b**). Genes listed in green indicate those associated with the FN-progenitor cancer stem cell signature (Progenitor) whereas red indicate those associated with differentiated muscle signature (Diff. Muscle) (**b**). **c** GSEA blots showing enrichment of the FN-progenitor cancer stem cell signature (Progenitor) and depletion of the differentiated muscle signature (Diff. Muscle) in RD OE MYOD1^L122R^ models. Similar results were seen in the RD HDR and Ruch2 OE models (Progenitor: NES (>2.26 and adjusted *p*-value 1 × 10^−10^ and Differentiated Muscle: NES <-2.56 and adjusted *p*-value <1.5 × 10^−10^)^11^. Quantification used GSEA statistics with adjusted *p*-values noted, as described by Subramanian et al. (2005), NES, Normalized Enrichment Score, NES>1 for up, NES<-1 for down. **d**–**i** Single-cell RNA-sequencing analysis of patient-derived xenograft (PDX) samples (FN-RMS with wildtype MYOD1 (*n* = 4)^8^ and those with mutant MYOD1^L122R^ (*n* = 4)). UMAP showing all 8 PDXs, with orange shading noting samples with wildtype MYOD1 and green for those with mutant MYOD1^L122R^ (also noted by stars). The 179 upregulated gene signature identified in engineered cell models was also significantly enriched in PDXs with MYOD1^L122R^ (**e**, **f**), **f** two-sided Student’s *t*-test was performed by comparing median values between MYOD1 and MYOD1L122R PDXs, ***, *p *< 0.001. Integration clustering and cell state annotation for all 8 scRNA sequencing of PDXs (**g**). Ternary plots showing only RMS-specific cell states comparing wildtype MYOD1 PDXs with mutant MYOD1^L122R^ PDXs (Proliferation, Progenitor, and Differentiated muscle cell states noted) (**h**). Quantification of progenitor score across PDXs with wild-type and mutant MYOD1 using a Student’s *t*-test (**i**), *p* < 2.2e-16. The box plots indicate the median (center line), the 25th and 75th percentiles (bounds of the box), and the whiskers extend to the minimum and maximum values within 1.5 times the interquartile range. Individual points represent outliers.

To study the transcriptomic changes induced by MYOD1^L122R^, we next performed bulk RNA-sequencing on each of the engineered models (Supplementary Fig. S7a, b, ≥3 replicates per sample). From this analysis, we identified 179 *MYOD1*^L122R^ upregulated genes and 265 downregulated genes across all models (≥2-fold up/down, adjusted *p* < 0.05, Supplementary Data 1 and Fig. 4a, b). Upregulated genes were enriched for epithelial-to-mesenchymal transition (EMT) genes that have previously been shown to be enriched in FN cancer stem cells^8^, while downregulated genes contained myogenesis and muscle differentiation genes^8,11^ (Supplementary Fig. 7c). We have also recently defined RMS-specific cell state for progenitor/cancer stem cells (CSCs) and differentiated muscle cells that do not drive tumor growth from single-cell transcriptomic profiles^11^. GSEA analysis using each of these signatures confirmed elevation of the FN-progenitor/CSC signature in all *MYOD1*^L122R^ models, with a reduction of the differentiated muscle gene signature (Fig. 4c and Supplementary Fig. S7d). This transcriptomic analysis further supports that *MYOD1*^L122R^ induces cancer stemness/progenitor signatures at the expense of myogenic differentiation programs.

To validate findings from our isogenic experimental models, we next analyzed single-cell RNA sequencing of FN-RMS and *MYOD1*^L122R^ SS-RMS PDXs (*n* = 4 per tumor subtype, total 55,500 cells, Fig. 4d and Supplementary Data 2). As expected, the genes upregulated in the MYOD1L^122R^ engineered models were also significantly enriched in *MYOD1*^L122R^ PDXs and patient samples (Fig. 4d–f). We next integrated the 8 PDX samples using a reciprocal PCA approach and annotated each cluster for cell state (Fig. 4g). We observed a reduction in the overall numbers of differentiated cells and a shift towards cells with progenitor cell state characteristics in *MYOD1*^L122R^ samples (Fig. 4h). MYOD1^L122R^ PDXs also contained higher fractions of progenitor cells and reductions in differentiated muscle cells compared to FN-RMS PDXs (*p* < 2.2 × 10^−16^, Fig. 4h, I). In total, these experiments confirm an overall elevation of known transcriptional cancer stem cell programs in *MYOD1*^L122R^ engineered and patient-derived models.

### MYOD1^L122R^ reprograms the RMS transcriptome by altering DNA binding specificity and redistributing histone modifications genome-wide

**Fig. 5:**
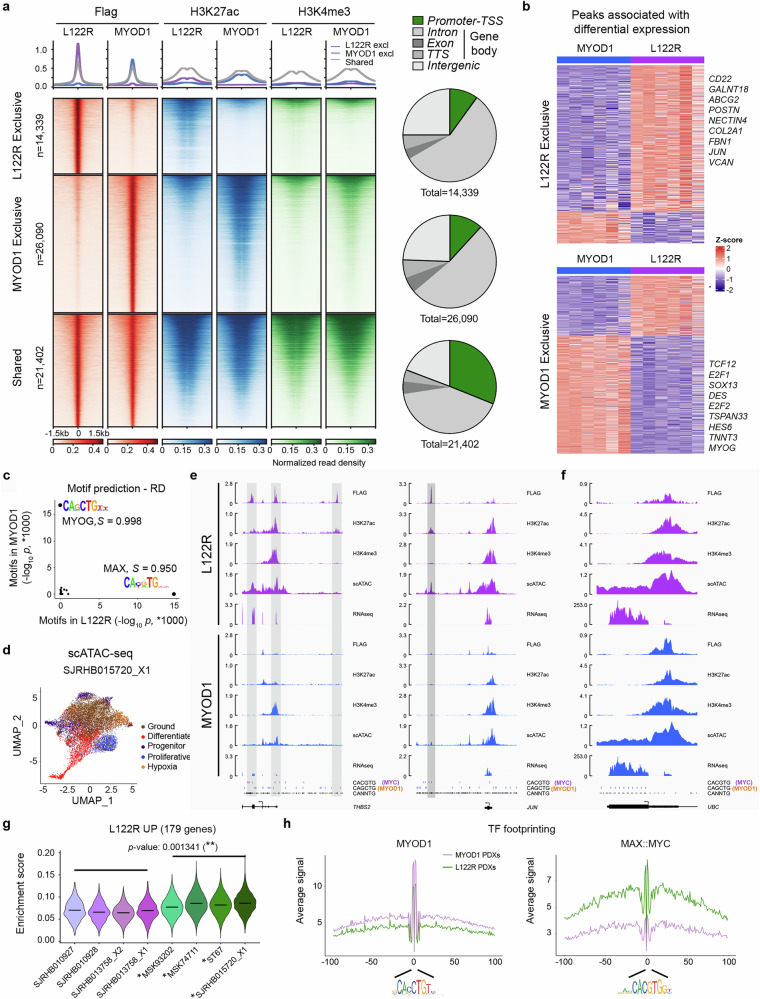
MYOD1^L122R^ alters DNA binding specificity, changes the histone modification landscape, and activates gene expression. **a** ChIP-seq from engineered RD cells that express flag-tagged MYOD1 or MYOD1^L122R^. Tornado plots comparing Flag, H3K27ac, and H3K4me3 binding across the genome (left). Pie charts detailing the class of genomic regions occupied exclusively by MYOD1^L122R^ (top), MYOD1 (middle), and co-bound by both (bottom). Promoter/TSS (transcription start site) is defined as spanning the transcription starting site and/or within −500 bp upstream (green shading). **b** Heat map showing bulk RNA expression of genes predicted to be bound to and presumably regulated by MYOD1^L122R^ or MYOD1. **c** Graph denoting differences in binding affinity for MYOD1 verses MYOD1^L122R^ based on analysis of exclusive binding sites in the genome and de novo motif prediction using HOMER. Similarly to top predicted, known motifs are noted, and similarity score indicated (S). **d** Representative example of scATAC-seq UMAP from a MYOD1^L122R^ PDX with predicted cells states annotated. **e**, **f** DNA occupancy map showing the correlation of FLAG, H3K27ac, H3K4me3 in RD cells and open chromatin regions identified by scATAC sequencing of PDX models for *THBS2* and *JUN* that are bound exclusively by MYOD1^L122R^ (**e**) or *UBC* that is bound by both MYOD1 and MYOD1^L122R^ (**f**). Motifs found in the genomic interval are noted at the bottom (MAX:MYC CACGTG in purple, MYOD1 CAGCTG in blue, and CANNTG highlighted in bold grey). RNAseq tracks correspond to expression of *THBS2*, *JUN*, and *UBC* in the RD cells. **g** Quantification of scATAC accessibility in PDXs. Combined open chromatin regions quantified across the 179 genes upregulated by MYOD1^L122R^, two-sided Student’s *t*-test was performed to compare medians of wildtype MYOD1 vs. mutant MYOD1^L122R^ (*n* = 4 each group, *p* = 0.001341). **h** Transcription factor footprinting from combined analysis of scATAC-seq open chromatin regions using the MYOD1 motif (left) and the MAX:MYC motif (right; combined analysis of 4 wildtype MYOD1 and 4 mutant MYOD1^L122R^ PDXs). Source data are provided as a Source Data file.

To unbiasedly assess the DNA targets of MYOD1^L122R^, we performed ChIP-sequencing (chromatin immunoprecipitation followed by next-generation sequencing) using engineered models that express doxycycline-inducible Flag-tagged MYOD1 or Flag-tagged MYOD1^L122R^ (RD, Fig. 5 and Ruch2, Supplementary Fig. S8). This assay identified many genomic loci that were exclusively bound by either MYOD1^L122R^ (14,339 in RD and 20,123 in Ruch2) or MYOD1^WT^ (26,090 RD and 22,656 in Ruch2, Fig. 5a, Supplementary Fig. S8a, and Supplementary Data 3 and 4), suggesting a neomorphic function for MYOD1^L122R^. We also found a large fraction of shared genomic regions bound by both wildtype and mutant MYOD1 (*n* = 21,402 in RD and *n* = 20,843 in Ruch2), indicating that MYOD1^L122R^ retains some wild-type transcriptional activity at many genomic loci. Shared peaks were enriched at transcription start/promoter sites, while MYOD1^L122R^ and MYOD1^WT^ exclusive peaks were more frequently located at distant sites within gene bodies or intergenic regions (Supplementary Fig. S9a, b). Finally, targets of MYOD1^L122R^ exclusive peaks included many genes found in the Progenitor signature, with 141 unique DNA-bind peaks corresponding to 73 Progenitor signature genes in RD cells (43.7%, *n* = 171 genes in signature, Supplementary Data 5), suggesting that MYOD1^L122R^ regulates progenitor pathways to alter SS-RMS biology.

To characterize the chromatin state of genomic regions bound by MYOD1^L122R^, we next performed ChIP-seq for activating histone marks histone 3 lysine 4 trimethylation (H3K4me3; a marker of active promoters) and histone 3 lysine 27 acetylation (H3K27ac, a marker of active promoters and intragenic/distal enhancers). A significant number of exclusive peaks bound by either MYOD1^L122R^ or wild-type MYOD1 were marked with only H3K27ac, suggesting that the specific differences between MYOD1^WT^ and MYOD1^L122R^ are mediated through enhancer or other regulatory elements (Fig. 5a and Supplementary Fig. S8a, b). In addition, RNA-seq from these same samples confirmed that gene loci near exclusive MYOD1^L122R^ or wildtype MYOD1 peaks were transcriptionally upregulated (Fig. 5b) only in the respective cell type. By contrast, shared peaks had characteristics of active promoters (H3K4me3 and H3K27ac signal), and were transcriptionally regulated in both cell line conditions (Fig. 5a and Supplementary Fig. S8a). Together, our data support a model where MYOD1 and MYOD1^L122R^ share transcriptional activator roles at gene promoters while neomorphic binding of MYOD1^L122R^ at distal regulatory sites leads to upregulation of differential transcriptional programs.

To unbiasedly define the DNA motifs found within the MYOD1 or MYOD1^L122R^ binding sites, we next performed de novo binding-motif predication from ChIP-seq peaks^28^. This analysis confirmed strong enrichment for the classic MYOD1/MYOG binding motif (-CAGCTG-) at sites exclusively bound by wildtype MYOD1 while mutant MYOD1^L122R^ peaks were enriched for the MAX:MYC motif (-CACGTG-) (Fig. 5c and Supplementary Fig. S9c). These preferences were further supported by protein structure prediction using AlphaFold (Supplementary Fig. S10a, b). By contrast, the shared peaks identified in our ChIPseq studies had a much weaker and degenerate MYOD1/MYOG or MAX:MYC binding motif consensus sequence (CANNTG), suggesting lower specificity binding mediated by complexes of interacting transcription factor machinery at the promoter transcriptional start sites. As representative examples, we examined two genes that have MYOD1^L122R^ exclusive binding sites, *THBS2* and *JUN* (Fig. 5e and Supplementary Fig. S11a). Both genes were highly expressed in our engineered *MYOD1*^L122R^ models, and DNA elements bound by MYOD1^L122R^ harbored the CACGTG motif (MAX:MYC) but not CAGCTG (MYOD1/MYOG). Consistent with distal regulation, MYOD1^L122R^ sites were only marked with H3K27ac but not H3K4me3 (Fig. 5e, Supplementary Fig. S11a). Single-cell ATAC seq confirmed that chromatin regions comprising the *THBS2* and *JUN* gene loci were also more open in MYOD1^L122R^ PDXs (Fig. 5d, e, Supplementary Fig. S11b, and Supplementary Data 2). We also examined the *UBC* genomic locus as a representative example of a gene bound by both MYOD1 and MYOD1^L122R^ at the TSS/promoter. This region was marked by both H3K27ac and H3K4me3 marks in cell lines and PDXs and enriched for the degenerative binding motif (CANNTG) but not MYOD1 (CAGCTG) or MYC (CACGTG) motifs (Fig. 5f and Supplementary Fig. S11b). In general, we observed that chromatin regions at MYOD1^L122R^ upregulated genes were more open and accessible in MYOD1^L122R^ PDXs when compared with MYOD1^WT^ FN-RMS (179 genes defined from RNA-seq, Fig. 5g). Transcription factor footprinting analysis from these same patient-derived xenografts further confirmed globally higher enrichment of the MYOD1^L122R^/MAX:MYC recognition motif within the open chromatin regions and overall lower enrichment of canonical MYOD1/MYOG motifs (Fig. 5h).

We next assessed if MYOD1^L122R^ alters the expression of c-MYC. RNA sequencing and Western blot analysis showed that c-MYC mRNA and protein expression are not differentially regulated between wildtype MYOD1 and MYOD1^L122R^ models (Supplementary Fig. S12a–f). In order to test whether c-MYC binds to MYOD1 or MYOD1^L122R^, we first performed proximity ligation assay (PLA), a technique that enables highly specific in situ detection of protein–protein interactions and conformational changes by using pairs of antibodies conjugated to oligonucleotides. When in close proximity ( < 40 nm), they undergo ligation and rolling circle amplification, generating a localized fluorescent signal that can be quantitatively imaged^29^. PLA confirmed known MYOD1:MYOD1 homodimerization, cooperative binding of MYOD1:MYOD1^L122R^ and c-MYC:MAX interactions; however, dimerization between c-MYC and either MYOD1 or MYOD1^L122R^ was not observed (Supplementary Fig. S12g, h). Control experiments confirmed, while single antibody experiments showed no PLA puncta.

We further performed co-immunoprecipitation experiments to test for cooperative binding of c-MYC with mutated MYOD1^L122R^. HEK293T cells were engineered to express either HA-MYOD1 or HA-MYOD1^L122R^ and FLAG-MYOD, and then co-immunoprecipitation performed using HA-conjugated beads. As expected, we confirmed MYOD1:MYOD1 homodimerization and cooperative binding of MYOD1:MYOD1^L122R^ (Supplementary Fig. S12i). By contrast, co-expression of HA-MYOD1 or HA-MYOD1^L122R^ along with FLAG-MYC followed by Western blot for antibodies for anti-FLAG or anti-HA (HA-MYOD or FLAG-MYC), demonstrated that MYOD1 or MYOD1^L122R^ do not bind to c-MYC, consistent with our PLA results (Supplementary Fig. S12j). Together, these findings suggest c-MYC is not altered in MYOD1^L122R^ cells nor associates with either MYOD1 or MYOD1^L122R^. Rather, our studies support that MYOD1^L122R^ alters genomic binding patterns by conferring a MYC-like binding preference and transcriptional regulation at distal enhancers.

### *ROR2* is a transcriptional target of MYOD1^L122R^ that elevates cancer stemness and therapy resistance

**Fig. 6:**
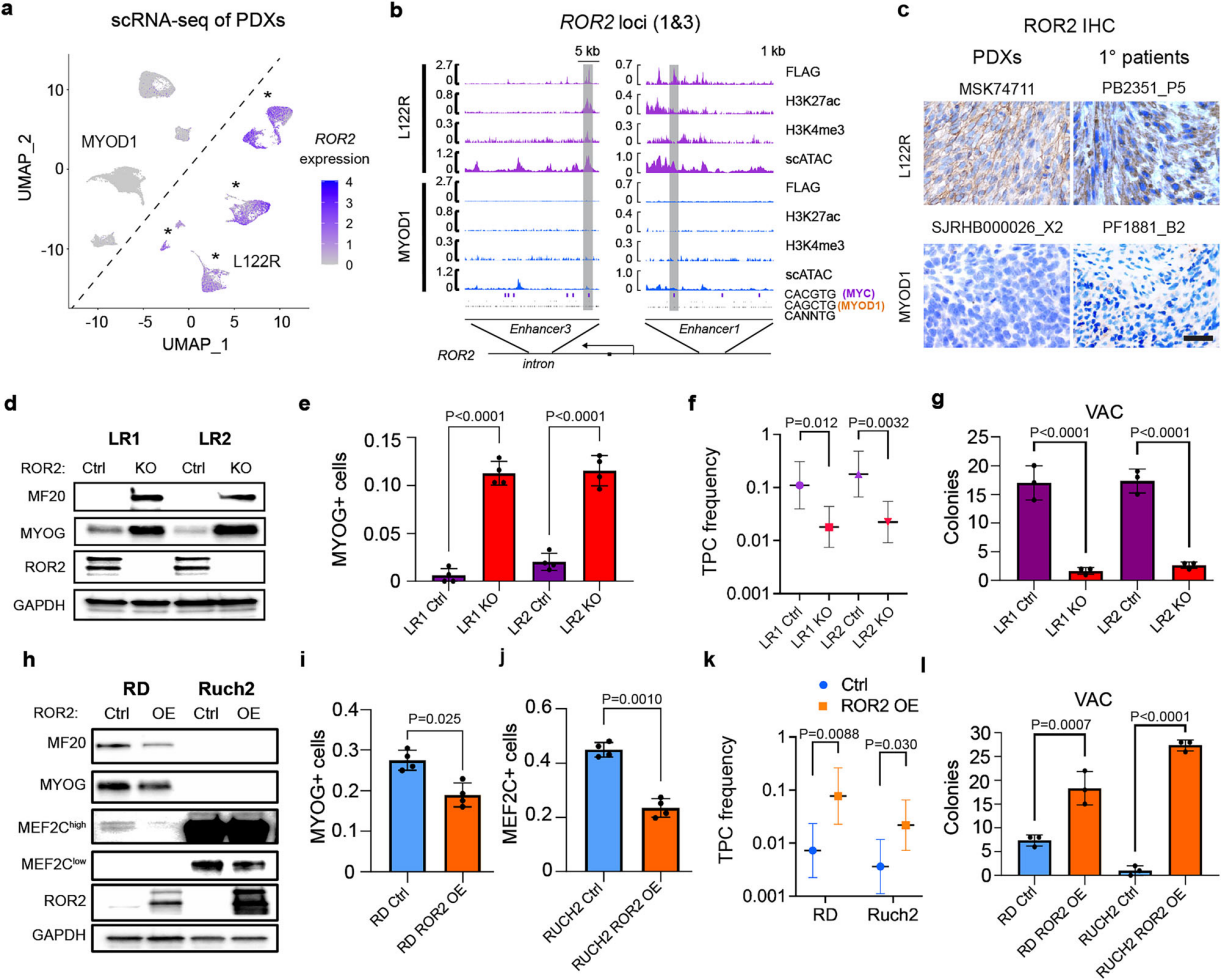
*ROR2* is a transcriptional target of MYOD1^L122R^ and regulates both cancer stemness and therapy-resistance. **a** Single-cell RNA-seq showing *ROR2* expression in four PDXs with MYOD1^L122R^ and four with wild-type MYOD. **b** DNA occupancy map showing the correlation of FLAG, H3K27ac, H3K4me3 in RD engineered models and juxtaposed with open chromatin regions identified by scATAC sequencing of PDXs. Motifs found in the genomic interval are noted at the bottom (MAX:MYC CACGTG in purple, MYOD1/MYOG CAGCTG in blue, and degenerated E-box CANNTG in grey). **c** Representative images of ROR2 immunohistochemistry (IHC) staining of patient-derived xenografts (PDX, left, *n* = 8), and primary patient samples (1°, right, *n* = 21), either with MYOD1^L122R^ (top), or MYOD1^WT^ (bottom), scale bar equals 50 µm. Additional IHC images and quantification are available in Supplementary Fig. 14. **d–g** Analysis of MYOD1^L122R^ HDR clones (LR1, LR2) following CRISPR/Cas9 inactivation of ROR2. Western blot analysis (**d**). Representative blots shown for two biological replicates with similar results. Quantification of immunofluorescence for differentiation marker MYOG in RD engineered models (**e**). Quantification of TPC frequency using limiting dilution tumorsphere assays and ELDA quantification (**f**). Clonogenic assays performed post-chemotherapy with VAC (**g**). **h–k** Analysis of differentiation markers (MYOG or MEF2C), stemness, TPCs, and response to VAC comparing parental cells RD and Ruch2 to those with ROR2 overexpression. Representative blots shown for two biological replicates with similar results (**h**). For IF experiments (**e**, **i**, **j**), there were four biological replicates; similar results were replicated in an independent experiment. For clonogenic assays (**g**, **l**), there were three biological replicates; similar results were observed twice in independent experiments. All tumor sphere assays (**f**, **k**) were conducted twice independently and similar results were observed (*n* = 6 biological replicates for **f** and *n* = 4 biological replicates for (**k**). One-way ANOVA followed by Tukey’s multiple comparison was used in (**e**, **g**). Student’s *t*-test was used for pairwise comparison in (**i**, **j**). mean±S.E.M. noted in **e**, **g**, **i**, **l**, or ELDA analysis in **f**, **k**, with markers note median and whisker plot lines ± 95% confidence interval. The samples derive from the same experiment but run on different gels for MYOD1, MF20, GAPDH, MEF2C, and ROR2 (**d**), with the exception of MF20 and MYOG (**h**) and ROR2 and GAPDH (**h**) that were run on the same gel. All samples were processed in parallel. *P* < 0.05 was considered statistically significant. Source data are provided as a Source data file.

We next selected genes that were upregulated and had a specific genomic peak associated with MYOD1^L122R^ neomorphic binding. Among them, we found *Receptor tyrosine kinase-like Orphan Receptor 2 (ROR2)* was (i) highly expressed in MYOD1^L122R^ patient and PDX RMS when assessed by bulk and single-cell RNA sequencing (Fig. 6a and Supplementary Fig. S13a–c), (ii) exclusively expressed in engineered MYOD1^L122R^ RMS models (Supplementary Fig. S13d), (iii) displayed open and accessible chromatin in MYOD1^L122R^ PDXs (Fig. 6b and Supplementary Fig. S13e–g), and (iv) was bound specifically by MYOD1^L122R^ at several loci in both cell line and PDX models (*ROR2* loci 1-3; Fig. 6b and Supplementary Fig. S6f, g). Confirmatory immunohistochemistry revealed that ROR2 protein was highly expressed and localized to the cell membrane of nearly all MYOD1^L122R^ mutated tumor cells in SS-RMS PDXs (*n* = 4 of 4) and primary patient samples (*n* = 13 of 13) (Fig. 6c and Supplementary Fig. S14). ROR2 positively regulates the non-canonical WNT/planar cell polarity (PCP) pathway in other developmental contexts^30,31^, and the WNT/PCP pathway has been shown to be dominant regulator of cancer stemness in FN-RMS^32^. Together, these findings nominated ROR2 as a top candidate to explore as a downstream and functional target of MYOD1^L122R^.

To test if *ROR2* has direct functional roles in regulating stemness, we next performed CRISPR/Cas9 knockout of *ROR2* in the RD HDR clones expressing mutant *MYOD1*^L122R^ (clones LR1 and LR2). Following *ROR2* knockout, we observed an increase in the differentiated muscle markers MYOG and MF20 by Western blot and an associated five-fold increase in the numbers of cells that express MYOG (*p* < 0.0001, Fig. 6d, e). *ROR2*-deficient cells also had reduced surface expression of CD44 and CD90 progenitor cell state markers (Supplementary Fig. S15a). Limiting dilution tumorsphere assays confirmed a significantly greater than five-fold reduction in the numbers of TPCs in *ROR2*-knockout MYOD1^L122R^ cells (Fig. 6f, *p* < 0.05). We then examined if these changes correlated to chemotherapy sensitivity using clonogenic assays. MYOD1^L122R^ models with intact *ROR2* signaling were resistant to both VAC and IVA (Fig. 6g and Supplementary Fig. S15c, g). In contrast, *ROR2* knockout cells could be effectively killed with both drug combinations, exhibiting a dramatic five-fold reduction in colony formation two weeks-post treatment (*p* < 0.001, Fig. 6g and Supplementary Fig. S15c, g). Gain-of-function experiments confirmed that *ROR2* overexpression in parental RD and Ruch2 cells resulted in the downregulation of differentiation markers (Fig. 6h–j), higher numbers of CD44+ CD90+ progenitors (Supplementary Fig. S15b), and greater than five-fold higher TPC frequency when assessed by limiting dilution tumor sphere assays (*p* < 0.05, Fig. 6k). Moreover, parental RD and Ruch2 cells that overexpressed *ROR2* were refractory to VAC and IVA treatment (*p* < 0.001, Fig. 6l, Supplementary Fig. S15d, h) and could not be efficiently killed by radiation, leading to robust numbers of colonies following 8 Gy radiation (Supplementary Fig. S15e, i). These data show that *ROR2* is a major downstream target of MYOD1^L122R^ that controls the overall numbers of CD44 + CD90+ progenitor cells, while simultaneously rendering tumors resistant to chemotherapy and radiation.

### A non-canonical WNT/PCP axis drives elevated self-renewal in MYOD1^L122R^ RMS

**Fig. 7:**
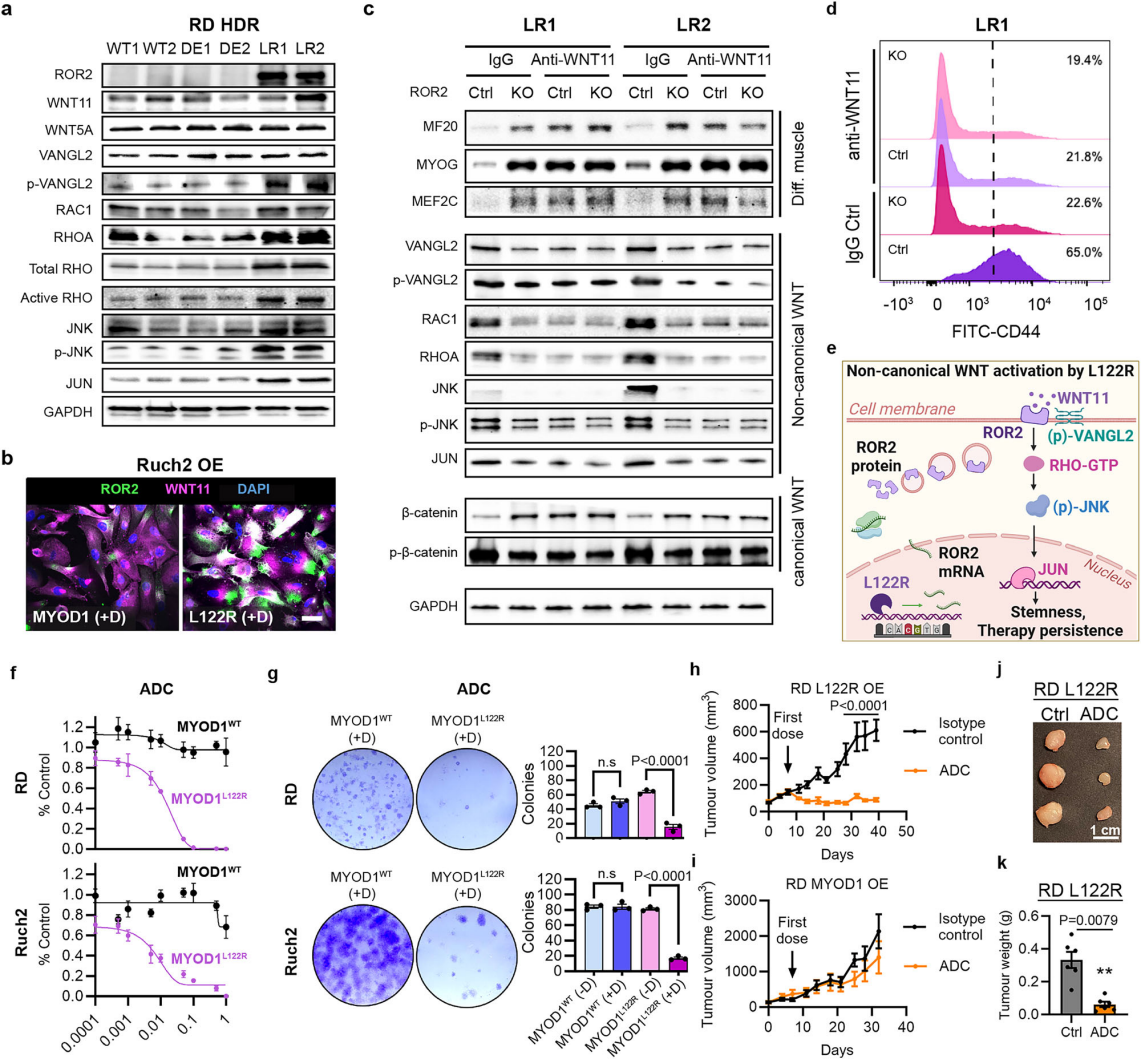
A therapeutically targetable non-canonical WNT/PCP pathway drives elevated cancer stemness and therapy persistence in MYOD1^L122R^ RMS. **a** Western blot analysis of HDR-modified cell models with known positive regulators of the non-canonical WNT/planar cell polarity pathway. Representative blots shown for two biological replicates with similar results. **b** Confocal images showing immunofluorescence co-localization of WNT11 (purple), ROR2 (green), and DAPI (blue) in Ruch2 cells that conditionally express MYOD1 or MYOD1^L122R^ after doxycycline-treatment (MYOD1 (+) and L122R (+), respectively). White notes pixel overlaps between WNT11 and ROR2, scale bar equals 10 µm. **c**, **d** WNT11 blocking antibodies inhibit ROR2 function and subsequently suppress the PCP pathway in MYOD1^L122R^ expressing cells. Engineered cells with knock-in of MYOD1^L122R^ (LR1) and intact or CRISPR/Cas9 depleted ROR2 were treated with control IgG or anti-WNT blocking antibody and assessed by Western blot (**c**) and flow cytometry for stemness marker CD44 for representative LR1 (**d**). Representative blots shown for two biological replicates with similar results. **e** Model for how MYOD1^L122R^ regulates the non-canonical WNT/planar cell polarity pathway to elevate cancer stemness and drive therapy-persistence in MYOD1^L122R^ mutated SS-RMS. Created in BioRender. Wei, Y. (2026) https://BioRender.com/f24v491. **f**, **g** ROR2 antibody-drug conjugate (ADC, Ozuriftamab vedotin) kills MYOD1^L122R^ expressing RMS. RD OE (top) and Ruch2 OE models (bottom). 48-hour growth assays after treatment (**f**) and clonogenic assays performed at 2-weeks post ADC treatment (**g**). For growth assays, data are mean ± STD from *n* = 3 biological replicates. For clonogenic assays, data are mean  ± SEM from *n* = 3 biological replicates. Both growth assays and clonogenic assays were replicated independently twice with similar results. **h–k** Ozuriftamab vedotin suppresses MYOD1^L122R^ tumor growth in xenografted NSG mice. Tumor size of mice engrafted with engineered RD models that express MYOD1^L122R^ (**h**) or MYOD1^WT^ (**i**) cells following doxycycline administration to the drinking water and four weeks of treatment with Ozuriftamab vedotin or control antibody. Images of resected tumors (3 of 6) at the end of studies. For these experiments, each model (WT or L122R) was engrafted into both flanks of three mice per group (isotype or ADC), for a total of *n* = 6 biological replicates. **j** Quantification of tumor weights (**k**, mean ± S.E.M.). Student’s *t*-test was used for pairwise comparison. The samples derive from the same corresponding experiment but different gels for ROR2, MF20, MYOG, MEF2C, WNT11, WNT5A, VANGL2, phospho-VANGL2, RAC1, RHOA, JNK, phospho-JNK, JUN, and GAPDH, and processed in parallel (**a**, **c**). Total RHOA/Active RHOA Western blots are derived from pulldown experiments from the same experiment (**a**, **c**). Not significant (ns). *P* < 0.05 was considered statistically significant. Source data are provided as a Source data file.

Our studies uncovered that MYOD1^L122R^ transcriptionally regulates *ROR2*, a well-known WNT-binding receptor that activates the non-canonical WNT/planar cell polarity (PCP) pathway^4^. Following the binding of WNT-ligands to ROR2, it promotes the phosphorylation and activation of VANGL2 (VANGL planar cell polarity protein 2) to induce the PCP pathway during morphogenesis^2,31^. Indeed, engineered and PDX MYOD1^L122R^ models activated the PCP pathway as exhibited by increased phosphorylation of VANGL2 (Fig. 7a and Supplementary Fig. S16a–c) and elevated levels of active RHOA (Fig. 7a and Supplementary Fig. S16a–c). As expected, JNK, phospho-JNK, and JUN, which are commonly activated in response to RHOA activation, were also upregulated in the MYOD1^L122R^ expressing cells (Fig. 7a and Supplementary Fig. S16a–c)^33–35^. These results are in keeping with studies from Hayes et al. showing the dominance of the VANGL2/RHOA pathway in driving cancer stemness in FN-RMS^32^ and show that ROR2 can further elevate this pathway, specifically in MYOD1^L122R^ mutated SS-RMS.

MYOD1^L122R^ expressing models had elevated transcription of non-canonical *WNT11* ligand, but not *WNT5A*. These changes in the *WNT11* transcript correlated to protein changes in the MYOD1^L122R^ engineered models and PDXs (Fig. 7a and Supplementary Fig. S16a–c). In addition, WNT11 and ROR2 co-localized at the cell surface in both engineered models that express MYOD1^L122R^ (Fig. 7b and Supplementary Fig. S16d–f) and PDXs models (Supplementary Fig. S16g). To test the hypothesis that WNT11 is a dominant regulator of the non-canonical WNT/PCP pathway in MYOD1^L122R^ RMS, we next used a recombinant antibody (anti-WNT11) to block its ligand activity and compared responses to cells treated with IgG control. MYOD1^L122R^ knock-in cells (LR1, LR2) were exquisitely sensitive to WNT11 inhibition, resulting in elevated expression of the differentiation markers MF20 and MYOG to comparable levels as ROR2 knockout cell line models (Fig. 7c and Supplementary Fig. S17a). In addition, WNT11 blocking also reduced the overall numbers of CD44+/CD90+ progenitors (Fig. 7d and Supplementary Fig. S17b). Western blot analysis confirmed that well-known non-canonical PCP signaling pathway components were also downregulated by WNT11 antibody blocking including VANGL2, RAC1, RHOA, JNK, JUN (Fig. 7c). The changes were also observed using flow cytometry, where CD44+ progenitor cells were reduced following incubation with anti-WNT11 (Fig. 7d). Co-immunoprecipitation experiments confirmed ROR2-VANGL2 associations, which could be enhanced by exogenous WNT11 or abrogated by anti-WNT blocking antibody (Supplementary Fig. S18a–c). Similar results were observed in ROR2 overexpression models where anti-WNT11 blocking antibody promoted the differentiation of cells and reduced stemness (Supplementary Fig. S17a, c–e).

### Therapeutic targeting of ROR2 in MYOD1^L122R^ mutated RMS

To evaluate ROR2 as a potential therapeutic target in RMS, we next assessed ROR2 in PDXs (*n* = 4) and primary patient samples (*n* = 13). Membrane ROR2 expression was detected nearly ubiquitously and membrane associated within all MYOD1^L122R^ tumor cells found in both PDXs and primary patient samples (Fig. 6c and Supplementary Fig. S14). Thus, we next assessed the efficacy of the ROR2-targeting antibody drug conjugate (ADC, Ozuriftamab vedotin) in killing MYOD1^L122R^ expressing RMS. This ADC delivers the tubulin inhibitor monomethyl auristatin E to ROR2-expressing tumors and is in phase II clinical trial evaluation for head and neck squamous cell tumors (NCT05271604). As expected, the naked Ozuriftamab antibody (anti-ROR2) was able to block ROR2 signaling and lead to a decrease in the overall numbers of CD44+/CD90+ cells, akin to the WNT11 blocking antibody (Supplementary Fig. S19a). Moreover, a single dose of Ozuriftamab vedotin effectively killed a wide array of MYOD1^L122R^-expressing RMS models, including engineered cell line models (Fig. 7f, g), a newly derived MYOD1^L122R^ patient cell line (JH-SRMS-7a, Supplementary Fig. S19b, c), and PDX spheres grown ex vivo (Supplementary Fig. S19d–f). In contrast, Ozuriftamab vedotin could not kill FN-RMS cell line models that lack ROR2 expression (Fig. 7f, g and Supplementary Fig. S19b–e).

We next tested whether ROR2-targeting Ozuriftamab vedotin affects RMS tumor growth in vivo. Specifically, we xenografted the RD dox-inducible models that express either wild-type MYOD1 or MYOD1^L122R^ into the flanks of NSG mice, allowed the tumors to engraft until palpable, and then treated mice with doxycycline added to the drinking water and weekly with Ozuriftamab vedotin. As expected, WT MYOD1 tumors that do not express ROR2 and were unaffected by the drug treatment. By contrast, MYOD1 ^L122R^ -expressing tumors regressed after treatment and did not grow back over the course of our studies (Fig. 7h–j). Moreover, at the culmination of the experiment, there was a significant five-fold reduction in tumor weight in comparing ADC treated MYOD1^L122R^ tumors to those that express wildtype MYOD1 (*p<0.0001* Fig. S7j, k and Supplementary Fig. S19h–j). Finally, we did not observe toxicity associated with ADC treatment as assessed by general health status and mouse weight measurements (Supplementary Fig. S19g). Taken together, our results show that targeting the ROR2 non-canonical WNT pathway using clinically available Ozuriftamab vedotin ADC is effective in preclinical mouse xenograft studies and likely warrants future clinical evaluation in MYOD1^L122R^ mutated SS-RMS.

## Discussion

Recurrent mutation of *MYOD1*^*L122R*^ in SS-RMS is associated with inferior prognosis irrespective of the patient’s age, sex, or other genetic mutations^15–17^. Consistent with clinical observations, MYOD1^L122R^ by itself failed to initiate tumors in zebrafish transgenic models, yet when combined with KRAS^G12D^, led to higher penetrance, worse survival, and elevated the overall frequency of tumor propagating cells. Activating mutations in RAS/PI3K or PTEN loss are all commonly found in MYOD1^L122R^ mutated ss-RMS^15,22^. In fact, a recent study found that 5 of 17 patients with MYOD1^L122R^ SS-RMS had RAS gene mutations, providing rationale for our approach to assess synergies between mutated MYOD1 and RAS in our zebrafish model. In total, our work suggests MYOD1^L122R^ is not sufficient to induce RMS, but is a modifying gene that elevates the overall aggression of tumors–rendering them therapy resistant and elevating the overall frequency of tumor propagating cancer stem cells.

Our studies went on to show that mutation of MYOD1^L122R^ alters binding specificity to MAX::MYC-like DNA recognition sites, consistent with previous reports showing a shift to this motif preference^22,36^. In keeping with DepMap studies showing that MYOD1 is a critical dependency in RMS, mutated MYOD1^L122R^ also binds to many of the same sites as wild-type MYOD1, ultimately turning on gene expression to maintain tumors in a transformed muscle-like state. Recurrent missense mutations in the DNA binding domains of transcription factors are rare in cancer compared to other driver mutations, yet have been described in some highly aggressive cancers. For example, KLF4^K409Q^ is found in secretory meningiomas^37^, GATA3^R276Q^ in T-ALL^38^, and IRF4^C99R^ in mutated B cell lymphoma^39^. These neomorphic transcription factors proport highly aggressive tumor subtypes, acquire new DNA binding site specificity and yet also retain a subset of wild-type gene function. Our work details the specific effects of MYOD1^L122R^ in RMS, suggesting that MYOD1^L122R^ is a representative example of this class of understudied and underappreciated cancer driver mutations.

The ROR2 non-canonical WNT-ligand receptor was identified as a neo-transcriptional target of MYOD1^L122R^, which activates the non-canonical planar cell polarity (PCP) WNT11-ROR2-VANGL2-RHOA signaling axis. The relationship between the PCP pathway and muscle cell development has been well-defined in animal models, but far less in malignancies, including SS-RMS. For example, skeletal muscle satellite stem cells self-renew after injury by activating the WNT7A-FZD7-VANGL2 PCP signaling pathway^3^. The elevated expression of WNT7A initiates satellite cell self-renewal and expansion of these cells, of which a subset differentiates and fuse to repair the injury. Indeed, ROR1—a related family member to ROR2 that also binds and activates VANGL2 to induce the PCP pathway—has been identified as a dominant regulator of satellite cell self-renewal and regeneration after injury^40^. Here, we find that MYOD1^L122R^ likely hyper-activates a similar PCP signaling axis to elevate CSC number and function in SS-RMS. These findings are in keeping with our prior work that defined roles for VANGL2 in controlling cancer stem cell pathways through the PCP pathway in FN-RMS, albeit without the need for ROR2 activation^32^. In the context of MYOD1 mutated SS-RMS, the non-canonical WNT11 ligand and the ROR2 receptor are both highly expressed and stimulate the ROR2-VANGL2-RHOA signaling complex to increase the overall number and self-renewal of CD44+/CD90+ cancer stem cells. Because these CD44+/CD90+ cells have been suggested to be inherently resistant to chemotherapy and radiation^7,8,11^, mutant MYOD1 associated SS-RMS would be predicted to be ineffectively killed by current clinical doses of therapy, likely accounting for why they are so aggressive and therapy-resistant in the clinical setting. Our results also support the planned clinical trial stratification of high-risk SS-RMS *MYOD1*-mutated patients into longer and more aggressive upfront therapy with VAC^41^. Our results also suggest differential sensitivities will extend to the setting of radiation and should be considered in these risk-based stratification approaches for modifying clinical course.

Our work uncovered the WNT11-ROR2-VANGL2-RHOA signaling pathway in MYOD1^L122R^ SS-RMS as a powerful regulator that simultaneously induces *both* cancer stem cell fate and therapy persistence. Indeed, recent work from our group and others has identified a progenitor cancer stem cell population in FN-RMS that is capable of remaking tumor following serum stress or xenograft implantation into immune-deficient mice^7–11^. This progenitor cell state was also correlated with therapy resistance in a small cohort of FN-RMS patients^11^, but to our knowledge, no studies have directly associated cell state changes with therapy response in either FN- or SS-RMS. Our finding that the WNT11-ROR2-VANGL2-RHOA signaling axis simultaneously increases stemness and therapy resistance supports a direct link between these processes in SS-RMS. Given the known presence of CD44+/CD90+ progenitor cells in FN-RMS, it will be important to directly link this cells state with therapy-persistence in patient tumors in the future.

ROR2 is highly expressed on the cell membrane in nearly all SS-RMS that harbor the MYOD1^L122R^ mutation, nominating it as a therapeutic target in this disease subset. Indeed, blocking antibodies to ROR2 suppressed pathway activation and led to tumors with lowered overall numbers of therapy-persistent CD44+/CD90+ cells. Moreover, antibody-drug conjugates that target ROR2 have been developed, including Ozuriftamab vedotin (CAB-ROR2-ADC; BA3021) that delivers Monomethyl auristatin E (MMAE) directly to tumor cells to inhibit cell division by blocking the polymerization of tubulin. Ozuriftamab vedotin has already passed phase I toxicity trials in adults and is being evaluated in Phase II clinical trials for aggressive head and neck squamous cell tumors (NCT05271604). Based on early successes in that trial, Ozuriftamab vedotin recently received FDA fast track designation for this use in that disease^42^ and suggests a path forward as a possible therapeutic opportunity for MYOD1^L122R^-mutated SS-RMS. Finally, ROR2 is also specifically expressed in osteosarcomas, breast cancer, leiomyosarcoma, and Ewing sarcomas, opening lines of investigation to assess the extent to which the ROR2-VANGL2-RHOA axis controls cancer stemness, therapy-resistance, and can be targeted therapeutically using ADCs in these tumor types^43^.

In summary, we have characterized the downstream effects of the neomorphic MYOD1^L122R^ transcription factor in RMS, which includes driving stem cell programs and blocking muscle differentiation programs. Moreover, our work has identified the MYOD1^L122R^ target ROR2 and non-canonical WNT signaling as a dominant self-renewal pathway in SS-RMS and identified ROR2-specific ADCs as a therapeutic opportunity in MYOD1^L122R^ mutated SS-RMS.

## Methods

### Human studies

Patient-derived xenograft models were obtained from Memorial Sloan Kettering Cancer Center (F.D.C), St. Jude Children’s Hospital (A.G.P)^7^, and Baylor College of Medicine (Dr. Nino Rainusso)^44^. For all patient samples, patient consents were signed and IRB protocols approved from these institutes. Consent to publish clinical information potentially identifying individuals was obtained, and samples analyzed under IRB protocols 2024P002814 (MGH) and XPD17-187 (St Jude). The mutant MYOD1 patient-derived cell line (JH-SRMS-7a) was obtained from John Hopkins (P.O and C.P) with patient consent and IRB protocols approved from that institution. Single-cell RNA-sequencing analysis used some previously published data sets (GSE195709 and GSE174376), see Supplementary Data 2 for details. Patient information was blinded to researchers in this study.

### Patient-derived xenograft studies

Mouse studies were approved by the MGH Institutional Animal Care and Use Committee (protocol 2013N000038). Mice were housed in the MGH CCM BCL2+ animal facility in the Charlestown Navy Yard (CNY)149 facility at a temperature of 21 °C (range between 18–24 °C), 30–70% humidity and lighting cycle on at 7 am and off at 7 p.m. No animals exhibited signs of distress such as weight loss, lack of movement or lethargy/weakness, inability to eat/drink, signs of severe pain, or labored breathing. In total, 1–5 × 10^5^ frozen, viable PDX cells were transplanted subcutaneously along with Matrigel into the flanks of three 6-week-old female NSG mice (100 μl). Engrafted tumors were monitored twice weekly by palpitation. Tumors were collected when they were less than 2 cm in diameter (4189 mm^3^ volume; maximal tumor size burden). Mice were euthanized by exsanguination under isoflurane anesthesia. A portion of the tumor was fixed in 4% PFA, and the remaining tissue used to isolate single cells. Specifically, tumors were macerated in RPMI medium supplemented with dissociation enzymes (Miltenyi Biotec, cat. no. 130- 095-929) and incubated at 37 °C for ~30 min. Single tumor cell suspensions were prepared and analyzed by scRNA-seq and scATAC-seq essentially as described^8^. Tumor cell preparation included dead cell and mouse cell removal^8^. All PDX tumors were analyzed for mycoplasma contamination (Lonza MycoAlert Mycoplasma Detection Kit) and by short tandem repeat (STR) analysis to validate identity (ATCC Human STR Profiling Cell Authentication Service). Models were also independently verified for MYOD1 mutation status using PCR and Sanger sequencing or direct analysis of mutations within the scRNA or bulk-RNA sequencing.

### Creation of transgenic zebrafish rhabdomyosarcoma

Zebrafish experiments were approved by the MGH Institutional Animal Care and Use Committee (Protocol 2011N000127). Microinjection of linearized plasmid constructs included *rag2:kRAS*^G12D^ and *rag2:GFP* DNA^45^. The transgenic constructs for *rag2:MYOD-T2A-tdTomato*, *rag2:MYOD1*^L122R^*-T2A-tdTomato, mcad:MYOD1*^L122R^*-T2A-tdTomato, myog:MYOD1*^L122R^*-T2A-tdTomato, and mylz2:MYOD1*^L122R^*-T2A-tdTomato* were generated by Gateway cloning using the zebrafish promoters^46^ and utilized the human MYOD1 open-reading frame or a T to G mutation encoding MYOD1^L122R^. Plasmid DNA was linearized using NotI and/or XhoI restriction enzymes (New England Biolabs), run on a 1% agarose gel, gel extracted, and re-suspended in 0.5x Tris EDTA + 0.1 M KCl. Concentration of microinjected DNA differed based on genetic background of zebrafish (2-4 pg for CG1 fish or 20–40 pg for Tu/AB fish, DNA was injected into one-cell stage embryos). Tumors were detected using epifluorescence microscopy starting at 15 days of life (Olympus MVX10). Differences in tumor onset and survival were assessed using the method of Kaplan-Meier (Graphpad Prism Survival).

### Cell transplantation of zebrafish tumors

Tumor cells were harvested, prepared, and transplanted by intraperitoneal injection into 8–10 syngeneic ~2-month old CG1-strain zebrafish (2 × 10^4^ cells/fish, 10 microliters/fish) and animals followed for tumor engraftment as previously described^32,47^. When recipient animals had engrafted tumors with sufficient tumor size, they were sacrificed, and tumor cells isolated by maceration and filtered over a 40-micron filter. Tumor cell suspensions were suspended in 0.9x PBS + 5% FBS, stained with DAPI to exclude dead cells, and sorted twice using a Laser BD FACSAria II Cell Sorter. Sort purity and viability were assessed after two rounds of sorting. Fluorescent tumor cells were injected at limiting dilution with each sample supplemented with 1 × 10^4^ RBC carrier cells. Secondary transplant fish were monitored for tumor engraftment under a fluorescent dissecting microscope from 10 to 120 days post-transplantation^32,47^. Tumor-propagating cell frequency was quantified using the Extreme Limiting Dilution Analysis (http://bioinf.wehi.edu.au/software/elda/).

### Histology and immunohistochemistry

Zebrafish and mouse xenografted tumors were fixed in 4% PFA, processed, and embedded in paraffin. Histological sections were made and stained with either Hematoxylin and Eosin or specific antibodies from the core panel at the MGH and BWH DF/HCC Research Pathology Cores, including cleaved caspase 3, phosphorylated histone 3, desmin (DES), and myogenin (MYOG). IHC of ROR2 were performed on FFPE PDXs, or primary patient samples with ABcam ab64264 HRP/DAB detection IHC kit. See Supplemental Table 7 for detailed antibody information. Pathology review and staging were completed by a board-certified sarcoma pathologist (G.P.N.).

### Creation of MYOD1L122R knock-in model human cell lines

Guide RNAs predicted by ChopChop^48^ to show high specificity and efficiency in proximity to 365 T nucleotide were purchased from Invitrogen. In total four guide RNAs were cloned into pSpCas9n (BB, Addgene #48139) backbone using the protocol previously described^24^. To assess CRISPR efficiency, we performed electroporation transfection with the gRNA and CAS9 vectors, and cells were grown for 3 days under puromycin selection, after which genomic DNA extracted, PCRed (forward primer – 5’-TCCTGAAACCCGAAGAGCAC-3’; reverse primer – 5’-GCTGGTTTGGATTGCTCGAC-3’), purified and analyzed by CRISPR sequencing (MGH DNA core) and CRISPResso2 (http://crispresso.pinellolab.org/submission)^49^. One guide RNA with the best efficiency was selected for downstream CRISPR-HDR (97.3% efficiency 5’-GCGCCGCTCGCGCATGGTGG-3’). A 92 bp homology double-stand DNA was designed to span the 365T>G (5’-CGC*CGACCGCCGCAAGGCGGCCACCATGCGCGAGCGGCGCCGCCGGAGCAAAGTAAATGAGGCCTTTGAGACACTCAAGCGCTGCAC*GTC-3’. Neon electroporation was then used to transiently introduce Cas9, guide RNA, and the homology-directed repair template into RD cells. Puromycin selection was used for 48 h after transfection to select Cas9-expressing cells. Single-cells were isolated by FACS and grown in 96-well plates. Following expansion, a portion of cells was used for genomic DNA extraction, and the genomic DNA sequence amplified using forward primer (5’-TCCTGAAACCCGAAGAGCAC-3’) and reverse primer (5’-GCTGGTTTGGATTGCTCGAC-3’). DNA amplicons were resolved on a 1% agarose gel, gel extracted (Gel extraction kit, QIAGEN), and sent for Sanger sequencing using the same forward and reverse primers.

### Creation of patient-derived mutant MYOD1 RMS cell line (JH-SRMS-7a)

The patient-derived RMS cell line JH-SRMS-7a was generated at Johns Hopkins University from a biospecimen collected during surgical resection from a pediatric patient with sclerosing spindle cell rhabdomyosarcoma with a mutation to MYOD1. Material was collected under an institutional review board (IRB)-approved protocol (IRB00486245), and the patient’s family provided written informed consent. JH-SRMS-7a was established as an in vitro cell culture without requiring passage through the mouse. The STR profile is provided as Supplementary Data 6.

### Lentiviral transduction to overexpress or knock out specific genes

Lentiviral constructs used in our work included: (1) dox-inducible plasmids to exogenously express wildtype MYOD1 (pInducer20-MYOD1-3xHA, Addgene #78328; pInducer20-Flag-MYOD1, Addgene #78330) or mutant MYOD1 which used site-directed mutagenesis to introduce the L122R mutation into these two plasmids, (2) stable overexpression of ROR2 using pLenti6.3-ROR2-V5 (DNASU Clone #HsCD00830884), and (3) validated CRISPR/Cas9 knockout plasmids from GeneScript (pLenti-CRISPR v2 guides targeting ROR2 (5’-AGCCGCGGCGGATCATCATC-3’, 5’-GGCCCGATTCCAACTCTGAA-3’, 5’-ATGAAGACCATTACCGCCAC-3’, 5’-CACTGAGAGCAGAAGCGCGG-3’). Each of these plasmids was transfected with 2nd generation lentiviral packaging plasmid (psPAX2, Addgene #12260 pMD.2 G, Addgene #12259) into HEK293T cells. Viruses were collected within 72 hours and used to infect RMS cells accordingly. Cells were virally-transduced and treated in bulk with Geneticin, Blasticidin, or Puromycin for 2 weeks to generate the stably infected cell lines. Western blot analysis was performed to validate overexpression or knockout in all experiments, and were shown in figures.

### Cell proliferation and viability assay

For cell proliferation experiments (corresponds to Fig. 3b), cells were initially seeded at a confluence of 10% into 96-well black-wall plates supplemented with DMEM/10% FBS/pen strep (RD) or RPMI/20% FBS/pen strep (RUCH2). After replating, cells were allowed to adhere for 2 h and treated with or without doxycycline (2 μg/mL for RD and 0.1 μg/mL for RUCH2). Overall viability was assessed at specified times using CellTiter-Glo Luminescent Assay (Promega Catalog # G7570, four replicate wells of each experimental group). Data was normalized to the day, 1 non-treated experimental group.

For IVA/VAC drug-response experiments (corresponds to Fig. 3b), 2 × 10^3^ cells were seeded into 96-well black-wall plates supplemented with DMEM/10% FBS/pen strep (RD) or RPMI/20% FBS/pen strep (RUCH2 and JH-SRMS-7a) (corresponds to Fig. 3b). Engineered cell lines (RD and RUCH2 expressing dox-inducible mutant MYOD1 or WT MYOD1) received doxycycline for 1 day after seeding. After this step, cells were treated with VAC or IVA at doses across a logarithmic range for 24 h. Cells were then put in fresh new media and overall viability was assessed after 4 days through the CellTiter-Glo Luminescent Assay (Promega Catlog # G7570). Plate reader was also used here to detect luminescence and collect data. Data is normalized to the untreated group.

### Tumorsphere assays

Cells were seeded onto 6-well low-attachment plates, Corning, Lot # 03824010 containing DMEM/F12 media Gibco catalog # 11320033 and supplemented with B27, EGF, bFGF. Media was replenished every three days, essentially as described^8,50^. Tumorspheres were counted between 20 and 30 days post seeding. ELDA was used to calculate TPCs (http://bioinf.wehi.edu.au/software/elda/).

### Clonogenic assays

A total of 200 or 300 RMS cells were seeded onto regular 6-well plates in RPMI/10%FBS/ PenStrep. After attachment for 24 h, colonies were treated either with 4.8 µM VAC (vincristine, actinomycin D, and cyclophosphamide) or 4.8 µM IVA (I-ifosfamide) prepared according to previous protocols^51^. A subset of experiments used 8 Gy of irradiation administered 24 h after seeding. VAC and IVA were administered for 24 h, and then fresh media added that did not contain drugs. After, media was changed every 3 days for a period of 14 days (chemo-) or 21 days (irradiation). At the end of each experiment, cells were fixed in methanol, stained with Crystal Violet (0.2% w/v in Acetic Acid, 30 min, Aqua Solutions, Inc, catalog # RPP013), and colonies manually counted. Each experiment group has at least 3 replicates, as indicated in figures.

### WNT11 and ROR2 antibody blocking experiments

The WNT11 blocking antibody was previously reported as having efficacy in studies using embryonic stem cell differentiation^52^ (Invitrogen; Catalog# PA5-21712). In our system, RD and Ruch2 cells were grown in 6-well plates and incubated with either 2.5 ng/mL of Rabbit IgG isotype control (Invitrogen; Catalog# 02-6102), or 2.5 ng/mL of rabbit polyclonal anti-WNT11 (Invitrogen; Catalog# PA5-21712), or naked human IgG1 Kappa anti-ROR2 antibody Oruriftamab (SelleckChem; Catalog# A3162) for 48 h. After 48 h of incubation, cells were washed twice with PBS and prepped for downstream analysis, including: flow cytometry (resuspended in 1% BSA containing PBS), immunostaining (fixed in 4% PFA for 20 min), or Western blot analysis (RIPA lysis for 30 min).

### ROR2 ADC Ozuriftamab vedotin treatments

To test the efficacy of antibody drug conjugates targeting ROR2, we used Ozuriftamab vedotin (Selleckchem; Catalog# D4033). For these experiments, RD and Ruch2 cells were used in both clonogenic assays and cell viability assays. For the clonogenic assay, the cells were initially treated with cell culture media containing 0.05 µg/mL of ADC for 24 h. Then, the media was swapped every 3 days and fresh media was added to the wells, over the course of a 2 or 3-week period. For the cell viability assay, 2 × 10^3^ RD, Ruch2, and JH-SRMS-7a cells were treated for 24 h with the ADC at varying concentrations (logarithmic scale) and swapped into fresh media for 96 h, after which, Cell-Titer Glo was used to measure cell viability. For the tumorsphere assays using PDX models, 1 × 10^4^ cells were seeded into 6- well low-attachment plates (Corning, Lot # 03824010) and spheres were grown for 2 weeks in DMEM/F12 media (Gibco catalog # 11320033) supplemented with B27, EGF, bFGF. Media was replenished every three days. After PDX spheres formed, wells were treated with 0.05 µg/mL of ADC or the isotype control over 7 days. After this, spheres were imaged and counted. After imaging, spheres were collected and disassociated, and then Cell-Titre Glo was used to measure viability from collected spheres. PBS treated spheres were used for normalization. Graphpad was used to generate response curves.

### Mouse xenograft and ADC testing

Doxycycline-inducible cell line models were transplanted subcutaneously into the flanks of 7-week-old female NOD PrkdcscidIl2rgtm1Wjl/SzJ mice (1 × 10^6^ RD-pInd20-MYOD1 or RD-pInd20-MYOD1^L122R^, 100 μl (cell resuspension in PBS), 3 mice per group, 2 tumors per animal). Mice were reared in a BCL2 facility as previously described^32^. Tumor volume was measured twice a week using caliper measurement^53^. When the tumor volume reached 100–150 mm^3^, mice were administered 1 mg/ml doxycycline-infused water at labium until the end of the experiment. After 3 days on doxycycline treatment, mice were injected by tail vein with ADC (Ozuriftamab vedotin, 1 mg/kg) or isotype control once weekly for four cycles. The experiment was terminated before the tumor diameter reached 20 mm, in accordance with institutional animal care guidelines. Necropsy was performed to collect tumors. Body weights were measured at least once a week. RD cell line was analyzed for mycoplasma contamination (MycoAlert Mycoplasma Detection Kit, Lonza) and by short tandem repeat (STR) analysis (Human STR Profiling Cell Authentication Service, ATCC) to confirm identify of the cell line.

### Co-immunoprecipitation (Co-IP)

The protocol for co-immunoprecipitation of VANGL2 and ROR2 was modified from literature^54^. The major deviation from the method included use of a modified lysis buffer that contained 200 mM NaCl, for a final composition of 20 mm Tris-HCl (pH 7.4), 200 mm NaCl, and 0.5% Nonidet P-40. RD cells overexpressing V5-tagged ROR2 were used for the pulldown experiments as described in lentiviral transduction section. Cells were grown in 10 cm dishes and transfected with 5 µg of MYC-tagged VANGL2 (cDNA purchased from Origene; Cat#RC212173) at 80% confluency using Lipofectamine 3000. Lysates were then prepped fresh and incubated with the different ChromoTek agarose beads conjugated to antibody (ChromoTek V5-Trap® Agarose; ChromoTek MYC-Trap® Agarose; ChromoTek Binding Control Agarose) for 4 h. The rest of the Co-IP was completed according to the manufacturer’s instructions (ChromoTek).

For assessing co-immunoprecipitation of MYOD1 and MYC, we transiently transfected HEK293T cells grown on 10 cm dishes in DMEM and 10% FBS (transfected at 80% confluency) using Lipofectamine 3000. Cells were co-transfected with either 4 µg of pInd20-MYOD1^WT^ or pInd20-MYOD1^L122R^ along with either 4 µg of pCMV4a-Flag-c-Myc (Addgene: #102625) or pCMV-FLAG-hMYOD1 (Addgene: #78329). Cells were swapped into fresh media the next day. After, HEK293T cells were grown in the presence of doxycycline (1 µg/mL) for 48 h to allow for expression of HA-MYOD1 vectors. Lysates were prepped fresh (2 days after dox-induction) and incubated with the ChromoTek HA-Trap Magnetic Agarose beads for 4 h. The rest of the Co-IP was completed according to the manufacturer’s instructions (ChromoTek).

### Proximity ligation assay

The dox-inducible HA-MYOD1 cells (RD-pInd20-MYOD1 or RD-pInd20-MYOD1^L122R^) were plated at a confluency of 1.5 × 10^5^ cells on coverslips and allowed to grow overnight in fresh media. The next day, cells were treated with 2 µg/mL of doxycycline for 3 days. Afterwards, the cells were fixed in PFA, permeabilized in 0.1% Triton X-100, and then the proximity ligation assay^29^ was performed using the Duolink® Proximity Ligation Assay kit (Millipore Sigma) according to the manufacturers protocol. Antibodies were first tested in a standard ICC experiment to determine optimal concentrations. For the different combinations, the antibodies used and dilutions for each antibody are specified in Supplementary Data 7.

### Chemical treatment to assess mechanisms of VANGL2 degradation

RD cells were plated on 10 cm dish at a confluency of 6 × 10^6^ cells and allowed to grow overnight in fresh media. The next day, cells were treated with either 600 nM bafilomycin A1 for 6 h (SelleckChem) or 3 µM MG-132 (MedChemExpress) for 12 h, then harvested and prepped by RIPA lysis for 30 min.

### Immunofluorescence staining, confocal imaging, and quantification

Cells were seeded in Matsumi glass bottom dish, fixed in 4% PFA for 15 min, followed by blocking (PBS supplemented with 2% goat serum, 0.2% TritonX-100) for one hour. Primary antibodies targeting MEF2C, ROR2, WNT11, and MYOG (see Supplementary Data 7 for antibody information) were incubated at 4 °C overnight, and secondary antibodies at room temperature for two hours. Both primary and secondary antibodies were suspended in 1x PBS that contained 1% goat serum and 0.1% TritonX-100. DAPI was used to stain nuclei of cells. A NIKON W1-SoRa spinning disk confocal microscope was used for imaging. Z-stack images with 0.5–1 µm per slice were taken for all images presented in the manuscript. Image J was used to process images, present data, and quantify results.

### Flow cytometry and Aldefluor assay for human engineered models

Cells were collected fresh for the flow analysis. Antibodies included PE-CD90 (BioLegend #328109) and FITC-CD44 (BioLegend #338803), used at the dilution of 1:200 in flow buffer (PBS, with 1% FBS and 1% NaN_3_). Aldefluor kit from StemCell technologies (Catalog# 01700) was used to detect ALDH activity in freshly collected cells. DEAB reagent was used to inhibit ALDH activity from the same kit to control for background fluorescence. DAPI was used to counter-select dead cells. The SORP 5-Laser BD LSRFortessa X-20 was used to perform flow analysis. FlowJo (v.10.10.0) was used for data analysis and presentation.

### Western blot analysis

Cultured cells were collected and lysed in either 2% SDS Buffer or RIPA Lysis Buffer, supplemented with protease inhibitors cocktail and phosphatase inhibitors. Protein concentration analysis was conducted using BCA protein assay. Protein concentrations ranged between 10 μg and 40 μg per well of protein and were combined with 4x Laemmli Buffer and lysis buffer. Samples were boiled at 100 °C for 10 minutes, quickly centrifuged, and then loaded on Mini-PROTEAN® TGX 4-20% pre-cast gel. Gels were transferred using Trans-blot Turbo Mini 0.2 µM PVDF Transfer. Membranes were blocked in either 5% Milk in TBST for 1 h at Room Temperature, or EveryBlot Blocking Buffer for 20 min. Blots were washed in between blocking and primary antibody incubation 3x with TBST. Primary antibody incubation was done with either 5 mL of 2% milk in TBST or 5 mL EveryBlot Buffer with antibody diluted to 1:1000 or up to 1:500 overnight at 4 °C, see Supplementary Data 7 for the list of antibodies used. Secondary antibody was incubated for 1 h at room temperature with fresh 5 mL of 2% milk in TBST or 5 mL EveryBlot Buffer after washing 3x with TBST. Membranes were developed using the enhanced chemiluminescent substrates Pierce ECL Western Blotting Substrate and/or SuperSignal West Femto Maximum Sensitivity Substrate. Developed films were imaged using ChemiDoc XRS+ Imaging System.

### RHO activity assay

The Cell Signaling Rho-Detection Kit (#8820) was used to assess RHO activity largely in accordance with the manufacture protocol. Briefly, adherent cultured cells were collected in 0.5 mL 1x Lysis/Wash Buffer + 1 mM PMSF per 10 cm petri dish. Protein concentration was measured using BCA protein assay (BioRad, Catalog # 5000113, 5000114, 5000115). 500 µg of cell lysates per reaction was used for positive and negative controls, as well as experimental samples. Lysates were incubated with either 0.1 mM GTPγS, 100 mM GDP, or without anything, supplemented with 10 mM EDTA, at 30 °C for 15 min with agitation. The reaction was terminated with 60 mM MgCl_2_. Then, reaction mixtures were added to spin columns containing a 50% glutathione resin and 400 µg GST-Rhotekin-RBD, followed by an incubation at 4 °C for 1 h with agitation. After incubation, samples were centrifuged at 300 × *g* for 30 s and washed with 400 µL 1x wash buffer 3 times. 50 µL of 2x reducing buffer (1:20 β-mercaptoethanol to 2x reducing buffer) was added to the spin column and incubated for 5 min at 100 °C, followed by elution via centrifugation at 300 × *g* for 30 s. Downstream analysis was done via Western Blot analysis.

### RNA- and ChIP-sequencing library preparation, quality check, and sequencing

RNA-seq samples were collected from 10^5^ cells using TRIzol (Invitrogen, catalog # 15596026) and then RNA extracted from cells using QIAGEN RNeasy kit according to the manufacture’s protocol (QIAGEN, catalog # 74104). Genomic DNA was removed using DNase I (Invitrogen, catalog # 18068015) for 1 h at 37 °C. TruSeq Stranded mRNA Library Prep kit (96 samples) was used to build mRNA libraries (Illumina # 20020595), and ChIP and Library prep Kits (Catalog #9005) from Cell Signaling Technology were used to prepare ChIP libraries, following the manufacture’s protocol. ChIP DNA libraries were then indexed with Dual Index 7 Primers and 5 Primers from Multiplex Oligos (CST# 47538) compatible with Illumina. AMPure XP and SPRI Select beads were used to clean up amplified DNA.

Qubit DNA assay was used to determine DNA concentration for libraries built from mRNA and from ChIP samples. Agilent 2100 Electrophoresis Bioanalyzer was used to check mRNA library size (~260 bp for RNA libraries, and ~150–900 bp, ladder pattern for ChIP libraries) and quality before sequencing run. NextSeq 2000, along with P3 reagents (100 cycles), was used for the sequencing run (Illumina # 20040559). On average, >20 M reads per sample were acquired, covering 75 bp from both ends for both RNA- and ChIP-seq libraries.

### Analysis of RNA sequencing

Initial FASTQ files were quality evaluated using FASTQC (v. 0.11.9)^55^. And all data were summarized using the ngsReports package (v. 2.0.3)^56^ in R (v. 4.2.2). Raw reads were trimmed based on quality and the presence of adapters using the trimmomatic tool (v. 0.39)^57^. Thus, for the elimination of the sequences of the adapters, the palindromic mode was used, considering a maximum mismatch of two nucleotides with a palindrome clip score threshold of 30 and a simple clip score threshold of 10. Additionally, quality trimming was performed using a window of 4 bp and an average quality score of 20. Very low-quality bases (Phred <3) were also removed from the beginning and the end of the reads. Reads with a length of less than 18 nucleotides were discarded. The surviving paired reads were aligned with the STAR aligner (v. 2.7.10b)^58^ against the human genome (GRCh38 version, Ensembl) using as gene reference a gene transfer format (gtf) file from Ensembl (v. 107). No filter for a minimum number of matched bases required for alignment (--outFilterScoreMinOverLread and --outFilterMatchNminOverLread were set to 0), so all alignments were included in the final BAM file. Read counting at the gene level was carried out using the htseq tool (v. 1.99.2)^59^ in its union mode for a reverse-stranded sequencing protocol. Count data were normalized using the DESeq2 (v. 1.38.3) package^60^ using the size factor method. Genes with low expression were removed in all analyses (at least five reads in four samples for contrast involving groups of six samples, five reads in three samples for contrast involving groups of five samples). Unsupervised hierarchical principal component analysis (PCA) and dendrogram were performed in R using the FactoMineR (v. 2.10) and factoextra (v.1.0.7) R Package Version 1.0.7. packages. Differential gene expression analysis was performed using DESeq2 through the default Wald test for two group comparisons. Gene expression differences were considered significant when Benjamini-Hochberg-adjusted p-values were below 0.05, and their log2 fold change was >1 between the MYOD1^L122R^ vs. MYOD1 wildtype conditions. In comparisons involving the parental cell groups, these thresholds were slightly modified, and genes were considered significant if their Benjamini-Hochberg-adjusted *p*-values were below 0.05 and their log2 fold change was over 0.58. Volcano plots obtained after this analysis were carried out with the EnhancedVolcano package (v. 1.16.0)^61^ R package version 1.16.0,. Ensembl gene IDs were transformed to HGNC IDs using the org.Hs.eg.db package (v. 3.16.0)^62^. Venn diagrams depicted to show the relationship between the significant genes were performed through the ggvenn package (v. 0.1.10)^63^. The heatmap representing the genes common to the three lists was carried out using the ComplexHeatmap (v. 2.18.0)^64^. We used the clusterProfiler (v. 4.6.2) package^65^ to perform gene set enrichment analysis (GSEA). Human Cancer Hallmarks from the msigdb gene set^66^ in its version 7.5.1 (released January 2022) obtained through the msigdbr package (v. 7.5.1) were used as pathway source. A custom database comprising genes involved in differentiation, proliferation, and progenitor genes was also used. Enriched pathways with a Benjamini-Hochberg adjusted p-value below 0.05 were considered significant. GSEA graphs were made using a modified version of the gseaplot2 function of the enrichplot package (v. 1.18.4), R package version 1.22.0,. Ridgeplots for Cancer Hallmarks were made using the ggridges package (v. 0.5.6). Individual gene box plots were generated using ggplot2 (v. 3.5.0).

### Analysis of ChIP-sequencing

The quality of the ChIP-seq reads was evaluated using the FASTQC tool. Reads were trimmed for quality and adapters removed using trimgalore (v.0.4.3) (https://www.bioinformatics.babraham.ac.uk/projects/trim_galore/). Cutadapt removes adapter sequences from high-throughput sequencing reads using default settings^67^. Alignment against the human genome (version hg38, Ensembl) was carried out using the BWA-MEM algorithm (v. 0.7.15)^68^, and the resulting SAM files were transformed to BAM, sorted by coordinates, and indexed using the samtools tool (v.1.4.1)^69^. Samtools was also used for the removal of the mitochondrial chromosome and unscaffolded regions. The cDNA-detector (v. 0.1.0)^70^ program was used to remove cDNA contamination in the BAM files. Duplicate marking was carried out as the sambamba tool (v. 1.0.1)^71^. Low-quality reads were removed with the FilterBAM tool (v. 2) by setting a Phred score of 30 as the minimum alignment quality. The determination of the ChIP-seq peaks for each of the histone marks was carried out with the macs2 (v. 2.0.10) program^72^ using the “callpeak” option and input samples as a control. Difficult to map regions were obtained from ENCODE (version GRCh38)^73^ and removed using the “subtract” command of bedtools (v. 2.30.0)^74^. The differential peaks between MYOD1^L122R^ and MYOD1 for the RD and Ruch2 cell lines, FLAG ChIP-seq, were obtained from the consensus between two analytical approaches. The first of these approaches was carried out using Macs2. First, the average of the fragment sizes was obtained using the “predictd” option on the different samples to be contrasted. Next, the pooling of the samples of the same condition (MYOD1^L122R^ or MYOD1) was carried out with the “callpeak” option, using the average fragment size obtained in the previous step and the input samples as a control. As a result, we obtained the effective sequencing depth for each of the conditions. Finally, the “bdgdiff” option of macs2 was used, specifying both the effective sequencing depth of each group (“--d2” option), the minimum distance to be considered between two significant peaks (60 bases in the case of the FLAG epitope, “-g” option) and the minimum length of the significant peaks (120 bases for FLAG, “-l” option). Peaks that obtained a q-value lower than 0.05 were considered significant. In the second approximation, differential peaks were called using the R package DiffBind (v. 3.8.4)^75^ together with edgeR (v. 3.40.2)^76^.The MACS2-TMM method was used as a method of normalization of the peaks, and the determination of the differential peaks between the different conditions was carried out by the adjustment of quasi-likelihood negative binomial generalized log-linear model. Peaks with a Benjamini-Hochberg-adjusted p-value lower than 0.05 were considered significant. To determine the consensus peaks between the two detection methods, we calculated the intersection of the peaks obtained from the two approaches using the bedtools tool in “intersect” mode. All selected peaks were annotated using the “annotatePeaks” function of HOMER (v.4.10)^28^. This approach allowed us to establish three groups of peaks for the FLAG epitope in the two cell lines: the exclusive peaks of MYOD1^L122R^ (selected in the macs2 approach as exclusive of “condition1”, and significantly enriched in the DiffBind approach [adj.*p* < 0.05 and Fold > 0]) the exclusive peaks of MYOD1 (selected in the macs2 approach as exclusive of “condition2”, and significantly enriched in the DiffBind approach [adj.*p* < 0.05 and Fold <0]) and the peaks shared between the two conditions (selected in the macs2 approach as “common” to the two conditions and not significant in the DiffBind approach [adj. *p* > 0.05]). Heatmap graphs were depicted following two approximations. In the first, the replicates for the same condition and histone marks were merged using the samtools “merge” option. In the second approach, each replicate was used independently. In both cases, bigwig files were generated using the “bamCoverage” option of the deeptools tool (v.3.1.2)^77^, with a bin size of 10 bp and applying a counts per million (CPM)-like normalization along with the “–centerReads” and “–ignoreDuplicates” options. The scores for each genomic region were calculated using the “computeMatrix” option also from deeptools, using an upstream-to-downstream distance of 1500 bp from the center of the interval. Finally, the “plotHeatmap” function was used to make the “tornado” graphs. Graphs focused on peaks located on specific genes, also integrating the scATAC-seq open chromatin regions, were made in R using the trackplot package (v. 1.5.01)^78,79^. The initial motif enrichment analysis of the exclusive peaks of the two conditions and the shared peaks was performed using HOMER in de novo discovery mode using a size of the region for motif finding of 200 bp.In a second step, we specifically tested enrichment of the discovered de novo motifs in shared and exclusive peak sets. This approach enabled us to interrogate the same set of motifs for peaks unique to each condition as well as those common to both, allowing us to assess their enrichment in the studied conditions. The final enrichment results were visualized using a scatter plot through ggplot2 in R. Finally, we proceeded to integrate the three peak groups with the corresponding gene expression data, considering the contrasts between the MYOD1^L122R^ and MYOD1 groups against the parental group. To achieve this, the lists of genes associated with peaks in each group were intersected with the differential expression data, only selecting protein-coding genes and removing duplicates. This process considered the log2 fold change and adjusted p-value as thresholds, as mentioned earlier. The result of this integration was visualized in the form of a stacked plot using ggplot2.

### Single-cell RNA-sequencing analysis

Single-cell RNA sequencing (scRNA-seq) raw base call (BCL) files retrieved from Illumina Basespace underwent demultiplexing and conversion into FASTQ files using the BCL Convert software (v2.0.0) provided by Illumina. Subsequently, the 10x Genomics Cell Ranger 7.1.0 pipeline was employed to conduct sequence alignment, basic read quality filtering, and counting of cell barcodes and unique molecular identifiers (UMIs). This process utilized both the human (GRCh38) and mouse (mm10) reference genomes downloaded from 10x Genomics website for the analysis of human patient-derived xenograft (PDX) samples. The pipeline generated outputs consisting of filtered cell barcodes and transcript identifiers, as well as read counts per cell and gene, which were utilized for subsequent downstream analysis.

The scRNA-seq matrices underwent analysis utilizing the Seurat v4 pipeline^80^. Each sample was subject to individual analysis. Since the PDX samples might contain mouse cells, we excluded the cells having >5% mouse reads for further analysis. Additionally, we selected the cells with the number of features ranging from 1000 to 8000, and the maximum allowed fraction of mitochondrial genes per cell was 20%. After the preprocessing step, log normalization was performed, and the top 2000 highly variable genes were identified using method vst with default settings. To avoid the domination of highly expressed genes, we scaled the data while regressing out confounding variables, including the number of UMIs, the number of genes, the percentage of ribosome and mitochondria genes, and a fraction of mouse reads. Next, we performed dimensionality reduction using principal component analysis (PCA). The first 20 principal components (PCs) were chosen to construct the K-nearest neighbor (KNN) graph with default settings. Louvain clustering was performed to identify clusters with resolution 0.5 (resolution = 0.5), and clusters were identified for each individual sample. We leveraged Uniform Manifold Approximation and Projection (UMAP) to visualize the scRNA-seq clustering results and meta-data information. To mitigate the possibility of doublets within the dataset, we employed the DoubleFinder algorithm^81^, utilizing optimized sample-specific pK numbers.

To determine cell identities for each cluster, we first identified differentially expressed genes (DEGs) for each individual cluster from each sample using FindAllMarkers function in the Seurat package with following parameters: only.pos = TRUE, min.pct = 0.25, logfc.threshold = 0.25, test.use = “wilcox”. Next, we annotated cell identities by comparing cluster specific DEGs with published canonical marker genes.

### Integration of multiple single-cell RNA-sequencing datasets

To merge scRNA-seq data originating from both MYOD1 and MYOD1^L122R^ samples, we aggregated all high-quality cells that met our predetermined quality control standards, as outlined in the single-cell RNA sequencing analysis section. This comprised 4 scRNA-seq datasets from MYOD1 FN-RMS samples and 4 from MYOD1^L122R^ samples (See Supplementary Data 2 for more details). Specifically, we combined raw count matrices utilizing the merge function available in Seurat v4^80^. This resulted in a total of *n* = 55,500 cells from *n* = 8 PDX samples.

After merging, log normalization was performed, and the top 2000 highly variable genes were identified using method vst with default settings. Subsequently, the data were scaled, and PCA was applied for dimensionality reduction. Based on the elbow plot, we selected the top *n* = 30 PCs to construct a KNN graph with 20 nearest neighbors (k.param = 20). The Louvain algorithm was then employed to identify clusters, with a resolution parameter set to 0.5 (resolution = 0.5). To visualize the cells and clusters on a two-dimensional embedding, we reduced the dimensionality using UMAP.

To mitigate batch effects across diverse samples, we aligned all 8 datasets employing Seurat’s integration pipeline, leveraging reciprocal PCA (RPCA) approach for the large datasets. This methodology, briefly outlined in the Seurat integration protocol, entails anchor identification between dataset pairs. Each dataset is projected into the PCA space of the other, and anchor constraints are enforced based on a shared mutual neighborhood criterion. We first conducted normalization and feature selection, retaining *n* = 2000 variable features for subsequent integration from each dataset. Next, data scaling PCA was conducted on each individual object. Anchors were identified using the FindIntegrationAnchors function with the following parameters: reduction = “rpca”, dims = 1:30. We generated the integrated object by utilizing the IntegrateData function, incorporating the top *n* = 30 PCs. We then scaled and centered the gene expression data, followed by PCA. We determined top *n* = 20 PCs for further analysis. A KNN graph was constructed, followed by clustering using the Louvain algorithm with a resolution parameter set to 0.2. UMAP was leveraged for visualization. To identify DEGs within each individual cluster, we utilized FindAllMarkers function available in the Seurat package, employing the following parameters: only.pos = TRUE, min.pct = 0.25, logfc.threshold = 0.25, test.use = “wilcox”. We annotated cell identities by comparing cluster specific DEGs with published canonical marker genes and gene set enrichment analysis.

### Gene signatures enrichment analysis

AUCell^82^ was employed to identify cells exhibiting active gene signatures at a single-cell resolution. Briefly, AUCell, a ranking-based method, uses the area under the curve to determine the gene set enrichment in individual cells. Having identified gene signatures from ChIP-seq data, we initially ranked all genes for each cell through the utilization of the AUCell_buildRanking function with default settings. Subsequently, the AUCell_calcAUC function was applied to compute the area under the curve for each gene signature across individual cells, with the top 5% of genes in the ranking being used. Similarly, we utilized the AUCell analytical framework to compute cell-state specific gene signature scores, including progenitor, proliferative, and differentiated cell states. These AUCell enrichment scores were leveraged for ternary plots. To evaluate the stemness of individual cells, we established a progenitor score following the methodology outlined^11^.

### Single-cell ATAC-sequencing analysis

Single-cell ATAC sequencing (scATAC-seq) raw base call (BCL) files retrieved from Illumina Basespace underwent demultiplexing and conversion into FASTQ files using the BCL Convert software (v2.0.0) provided by Illumina. Subsequently, the 10x Genomics Cell Ranger ATAC 2.1.0 pipeline was employed to preprocess the raw data. This process utilized both the human (GRCh38) and mouse (mm10) reference genomes for the analysis of human PDX samples. The pipeline generated outputs consisting of filtered cell barcodes and peak coordinates, as well as read counts per cell and peak, which were utilized for subsequent downstream analysis.

The scATAC-seq matrices were utilized for downstream analysis using the Signac pipeline^83^, with each sample analyzed individually. Due to the potential for mouse cell contamination in the PDX samples, features associated with mice were omitted, and any mouse cells identified through the 10x Genomics cellranger-atac pipeline were subsequently excluded. To assess the quality of the scATAC-seq data, various quality control metrics were computed. These metrics included nucleosome signal score, transcriptional start site (TSS) enrichment score, the total number of fragments within peaks, the fraction of fragments within peaks, and the ratio of reads in genomic blacklist regions. Subsequently, the scATAC-seq data underwent a stringent filtering process to retain only high-quality cells. Specifically, cells meeting the following criteria were subjected to further analysis: peak region fragments between 1500 and 10,000, a blacklist fraction below 0.05, at least 40% of reads within peaks, nucleosome signal below 2, and TSS enrichment above 2. After QC, normalization was conducted using the RunTFIDF function with default parameters. A subset of the top *n*% of peaks was selected using the FindTopFeatures function with a minimum cutoff of q0. Dimensionality reduction was then performed via singular value decomposition (SVD) using the RunSVD function. It was observed that the first latent variable from SVD primarily reflected sequencing depth, indicating technical rather than biological variability. Therefore, the first component was excluded from further downstream analysis. To embed the cells into a low-dimensional space, we utilized graph-based clustering and non-linear dimensionality reduction for visualization with the following parameters: reduction = “lsi”, dims = 2:20.

To assess the activity of individual genes within the genome based on their associated chromatin accessibility, we calculated a gene activity matrix for each individual samples using GeneActivity function. Briefly, this function counts the fragments attributed to each cell, which map to regions corresponding to gene coordinates, extending to encompass the 2 kb upstream region. We utilized the gene activity matrix derived from scATAC-seq data and integrated it with the corresponding scRNA-seq data from the sample through cross-modality integration and label transfer approaches. Specifically, we identified anchors between the scATAC-seq dataset and the scRNA-seq dataset using the FindTransferAnchors function with reduction = “cca”.

### Single-cell ATAC-sequencing analysis from published data

To compare the four MYOD1^L122R^ PDX samples with the FN- RMSMYOD1 samples, we utilized four publicly available scATAC-seq datasets (SJRHB010927_X1, SJRHB010928_X1, SJRHB013758_X1, and SJRHB013758_X2) sourced from the St. Jude Childhood Solid Tumor Network^7^. We retrieved publicly available FASTQ files from GEO accession: GSE174376 and implemented the same 10x Genomics Cell Ranger ATAC 2.1.0 pipeline used for our PDX samples.

Similarly, the cellranger-atac output matrices were subjected to downstream analysis using the Signac pipeline, with each MYOD1 sample analyzed individually. Due to the potential for mouse cell contamination in the PDX samples, features associated with mice were omitted, and any mouse cells identified through the 10x Genomics cellranger-atac pipeline were subsequently excluded. Before conducting dimensionality reduction, cells meeting the following criteria were subjected to further analysis: peak region fragments between 1000 and 20000, a blacklist fraction below 0.05, at least 20% of reads within peaks, nucleosome signal below 1, and TSS enrichment above 2. Following QC steps, we implemented the Signac pipeline with uniform parameters for dimensionality reduction and gene activity calculation, maintaining consistency with those applied to our PDX samples.

### Transcription factor footprinting

Footprints are generated by TFs bound to DNA, which inhibits the Tn5 transposase from cleaving DNA within nucleosome-free regions. The HINT-ATAC algorithm^84^, a Hidden Markov Model (HMM)-based approach, was utilized to discern TF binding sites associated with footprints.

In order to delineate transcription factor (TF) footprints within individual samples employing HINT-ATAC, we initially prepared indexed BAM files and peak files derived from the aggregation of cells originating from each respective sample. Peak files were generated using the MACS2 callpeak function with the following parameters: -g hs -q 0.01 --nomodel --shift -100 --extsize 200 -B --SPMR --call-summits. Subsequently, we initiated footprints identification by running the function rgt-hint footprinting specifying parameters including: --atac-seq --paired-end --organism=hg38. Following footprint calling, TF-associated footprints within each sample were discerned through the detection of motifs sourced from the JASPAR vertebrates database that overlapped with the predicted footprints, facilitated by the rgt-motifanalysis matching function. Finally, to ascertain average single-cell ATAC sequencing (scATAC-seq) profiles surrounding the binding sites of each TF across individual samples, we applied the rgt-hint differential function with the following parameters: --organism=hg38 --bc --nc 32.

To identify the differences in transcription factor (TF) activities between MYOD1 and MYOD1^L122R^ samples, we utilized publicly available MYOD1 scATAC-seq datasets from the St. Jude Childhood Solid Tumor Network. Specifically, we generated an indexed BAM file for the MYOD1^L122R^ samples by aggregating data from MSK93202, MSK74711, MAST161, and ST67 using samtools merge function^69^. Similarly, we merged all MYOD1 samples, encompassing SJRHB010927_X1, SJRHB010928_X1, SJRHB013758_X1, and SJRHB013758_X2, to generate another indexed BAM file. Subsequently, we generated corresponding peak files utilizing the MACS2^72^ callpeak function. We employed the same analytical HINT-ATAC workflow and predicted TF footprints across both MYOD1 and MYOD1^L122R^ samples.

### AlphaFold2 prediction

The amino acid sequence of MYOD1 and its corresponding DNA sequence were obtained from RCSB PDB using code 1MDY^85^. Residues 101-169 were used in these models. DNA sequences used in the WT and CG swap models were TCA ACA GCT GTT GA and TCA ACA CGT GTT GA, respectively. The amino acid sequences were entered into the Colab notebook version of AlphaFold v2.3.2^86,87^ with the link https://colab.research.google.com/github/deepmind/alphafold/blob/main/notebooks/AlphaFold.ipynb, and GitHub codes https://github.com/sokrypton/ColabFold. Generated models were validated using PROCHECK via the UCLA-DOE lab server^88^, https://saves.mbi.ucla.edu/. DNA models were built using PyMOL 2.5.4. Four different MyoD-DNA complex models were generated using the Huang Laboratory’s HDOCK server^89–93^, with the link http://hdock.phys.hust.edu.cn/. MYOD-DNA complex surface models were rendered using the PyMOL rendering plugin (https://pymolwiki.org/index.php/Rendering_plugin). The models were analyzed, animated, and imaged using PyMOL 2.5.4.

### Statistics and reproducibility

Data shown in figures with error bars and statistics represent analysis from biological replicates and are annotated in more detail in the Source Data files. Technical replicates were not included in the analysis for the paper. Independent replication of experiments is noted at the end of each legend. Mean/median/average are noted each figure legend, and exact *p*-values embedded within each figure. Single-cell RNA-seq and ATAC-seq were only performed in one patient-derived sample, considering the availability of the patient samples and the cost of the experiment, with 5000–10,000 cells analyzed per experiment. ChIP-seq experiments were performed in duplicate, and RNA-seq was performed in at least triplicate for individual experimental groups. For AlphaFold 2 prediction, analysis was performed twice, independently by A.D.W, who was blinded from this research findings the time of running the experiment. No experimental results were excluded.

### Reporting summary

Further information on research design is available in the Nature Portfolio Reporting Summary linked to this article.

## Supplementary information

Supplementary Information

Reporting Summary

Description of Additional Supplementary Files

Supplementary Data 1-7

Transparent Peer Review file

## Source data

Source Data

## Data Availability

The raw and processed RNA-seq and ChIP-seq data generated in this study from rhabdomyosarcoma cell lines have been deposited in the NIH Gene Expression Omnibus (GEO) database under accession code GSE274640. The scRNA-seq data generated in this study include three mutant MYOD1^L122R^ patient-derived xenograft (PDX) samples: MSK93202, SJRHB015720_X1, and ST67. These data have also been deposited under GEO accession code GSE274640. Additional scRNA-seq data from four MYOD1 PDX samples, SJRHB000026_X1, SJRHB000026_X2, SJRHB013758_X2, and SJRHB013758_X1, as well as one MYOD1^L122R^ PDX sample, MSK74711, were obtained from a previously published dataset under GEO accession code GSE195709^8^. The scATAC-seq data generated in this study include four mutant MYOD1 ^L122R^ PDX samples: MSK93202, MSK74711, SJRHB015720_X1, and ST67. These data have been deposited under GEO accession code GSE274640. Additional scATAC-seq data from four MYOD1 PDX samples, SJRHB010927_X1, SJRHB010928_X1, SJRHB013758_X1, and SJRHB013758_X2, were obtained from a previously published dataset under GEO accession code GSE174376^7^. All previously published datasets of the PDXs used in this study are also described in Supplementary Data Table 2. Source data are provided with this paper.

## Code availability

The codes used in the study are deposited in the GitHub database under accession code [https://github.com/rheinbaylab/Wei_MYOD_RMS]. Code is also available under the zenodo 10.5281/zenodo.18882117.
