# Supplementary material for: MYOD1 mutation drives cancer stem cell pathways and therapy-resistance in spindle cell/sclerosing rhabdomyosarcoma
Source: Nat Commun. 2026 Jun 3;17:7129. doi: 10.1038/s41467-026-73546-7 (PMC13396186; doi:10.1038/s41467-026-73546-7)
Supplement: Supplementary file 1 — Supplementary Information [file 41467_2026_73546_MOESM1_ESM.pdf]

SUPPLEMENTARY INFORMATION

MYOD1 Mutation drives Cancer Stem Cell pathways and Therapy-resistance in Spindle cell/Sclerosing Rhabdomyosarcoma

Yun Wei[1,2,3†], Luis Antonio Corchete Sánchez[2,4†], Sabateeshan Mathavarajah[1,2,3†], Diego Antelo[1,2], Shuze Wang[1,4], Jihee Lee[1,2,3], Alexander Daiki Weissman[1,2], Devika D. Kannambadi[1,2], Qian Qin[2,4], Sara G. Danielli[4,5], Elisa J. Quantin[1,2], Tiffany C. Eng[1,2,3], Alexandra Veloso[1,2,3], Yueyang Wang[1,2,3], Gunnlaugur P. Neilsen[6], Chuan Yan[7], Valerie Shiwen Yang[7,8,9], Anand G. Patel[10,11], Selene C. Koo[12], Patience Odeniyide[13], Christine A. Pratilas[13], Miguel N. Rivera[1,2], Esther Rheinbay[1,2,4*], David M. Langenau[1,2,3*]

[1]Molecular Pathology Unit, Massachusetts General Research Institute, Harvard Medical School, Charlestown, MA 02129.
[2]Krantz Family Center for Cancer Research, Massachusetts General Hospital, Charlestown, MA 02129.
[3]Harvard Stem Cell Institute, Harvard Medical School, Cambridge, MA 02139.
[4]Broad Institute of MIT and Harvard, Cambridge, MA 02142.
[5]Department of Pediatric Oncology, Dana-Farber Boston Children's Cancer and Blood Disorders Center, Boston, MA 02215.
[6]Center for Sarcoma and Connective Tissue Oncology, Department of Orthopedic Surgery, Massachusetts General Hospital, Boston, MA, 02114.
[7]Institute of Molecular and Cell Biology (IMCB), Agency for Science, Technology and Research (A*STAR), Singapore, 138673
[8]Division of Medical Oncology, National Cancer Centre Singapore, Singapore 168583
[9]Oncology Academic Clinical Program, Duke-NUS Medical School, Singapore 169857
[10]Department of Developmental Neurobiology, St. Jude Children's Research Hospital, Memphis, TN 38105, USA.
[11]Department of Oncology, St. Jude Children's Research Hospital, Memphis, TN 38105, USA.
[12]Department of Pathology, St. Jude Children's Research Hospital, Memphis, TN 38105, USA.
[13]Division of Pediatric Oncology, The Sidney Kimmel Comprehensive Cancer Center, Johns Hopkins University School of Medicine, Baltimore, MD 21287, USA
Correspondence to:

David M. Langenau, Ph.D.
Professor of Pathology, Harvard Medical School
dlangenau@mgh.harvard.edu

Esther Rheinbay, Ph.D.
Assistant Professor, Harvard Medical School
Associate Member, Broad Institute
erheinbay@mgh.harvard.edu

† These authors contributed equally. * These authors jointly supervised this work.

Supplementary Information

Supplemental Materials and Description

Supplementary Figures 1-19, with legends.

Supplementary Tables

Supplementary Table 1. $MYOD1^{L122R}$ alone is not oncogenic in zebrafish models.

Supplementary Table 2. Zebrafish with $MYOD1^{L122R}$ tumors have lower median survival.

Supplementary Data 1. Genes that are differentially regulated (DEGs) by $MYOD1^{L122R}$ based on bulk RNA-sequencing of engineered cell models, log2 Fold Change (log2FC) for magnitude (positive = up, negative = down) and adjusted p-values (padj) for significance, cutoffs, log2FC > 1 and padj < 0.05 to identify Differentially Expressed Genes (DEGs), statistical method used is DESeq2.

Supplementary Data 2. PDX and patient samples used in our work.

Supplementary Data 3. ChIP-seq binding sites of flag-tagged wildtype or mutant MYOD1 in RD cells.

Supplementary Data 4. ChIP-seq binding sites of flag-tagged wildtype or mutant MYOD1 in Ruch2 cells.

Supplementary Data 5. Progenitor genes that are specifically bound by mutant $MYOD1^{L122R}$.

Supplementary Data 6. STR profile and identified mutations for patient-derived spindle cell/sclerosing RMS cell line JH-SRMS-7a.

Supplementary Data 7. Antibodies used for Western blot, immunofluorescence, flow analysis and/or ChIP-seq.

Source data tables for individual figure panels.

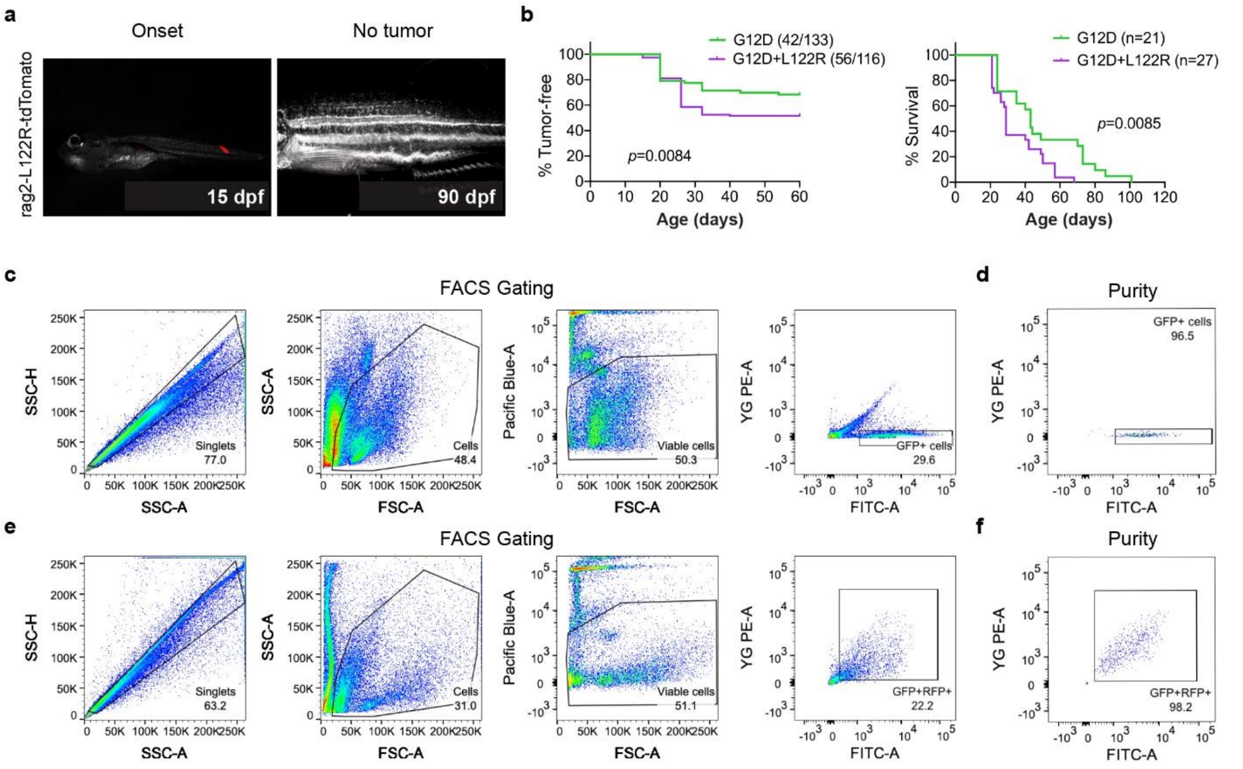

Supplementary Figure 1. MYOD1^{L122R} alone does not drive oncogenic transformation when expressed in transgenic zebrafish. a) Representative merged brightfield and fluorescent image of a Tu/AB (Tuebingen/AB) fish injected with *rag2:MYOD^{L122R}-T2A-tdTomato* and assessed at 15 days (onset) and at 90 days post days post fertilization (dpf, No tumor). See also Supplementary Table 1. **b**) Tumor onset (left) and tumor survival (right) for CG1-strain zebrafish injected with *rag2:KRAS^{G12D}* + *rag2:GFP* (G12D) or with *rag2:KRAS^{G12D}* + *rag2:GFP* + *rag2:MYOD^{L122R}-T2A-tdTomato* (G12D+L122R). **c,e**) Representative example of FACS gating strategy used for sorting GFP+ (c, FITC-A) and GFP+tdTomato+ (e, FITC-A and YG PE-A) tumor cells. **d, f**) Flow cytometry analysis of cells after sorting to assess overall purity. Source data are provided as a Source Data file. $P < 0.05$ was considered statistically significant.

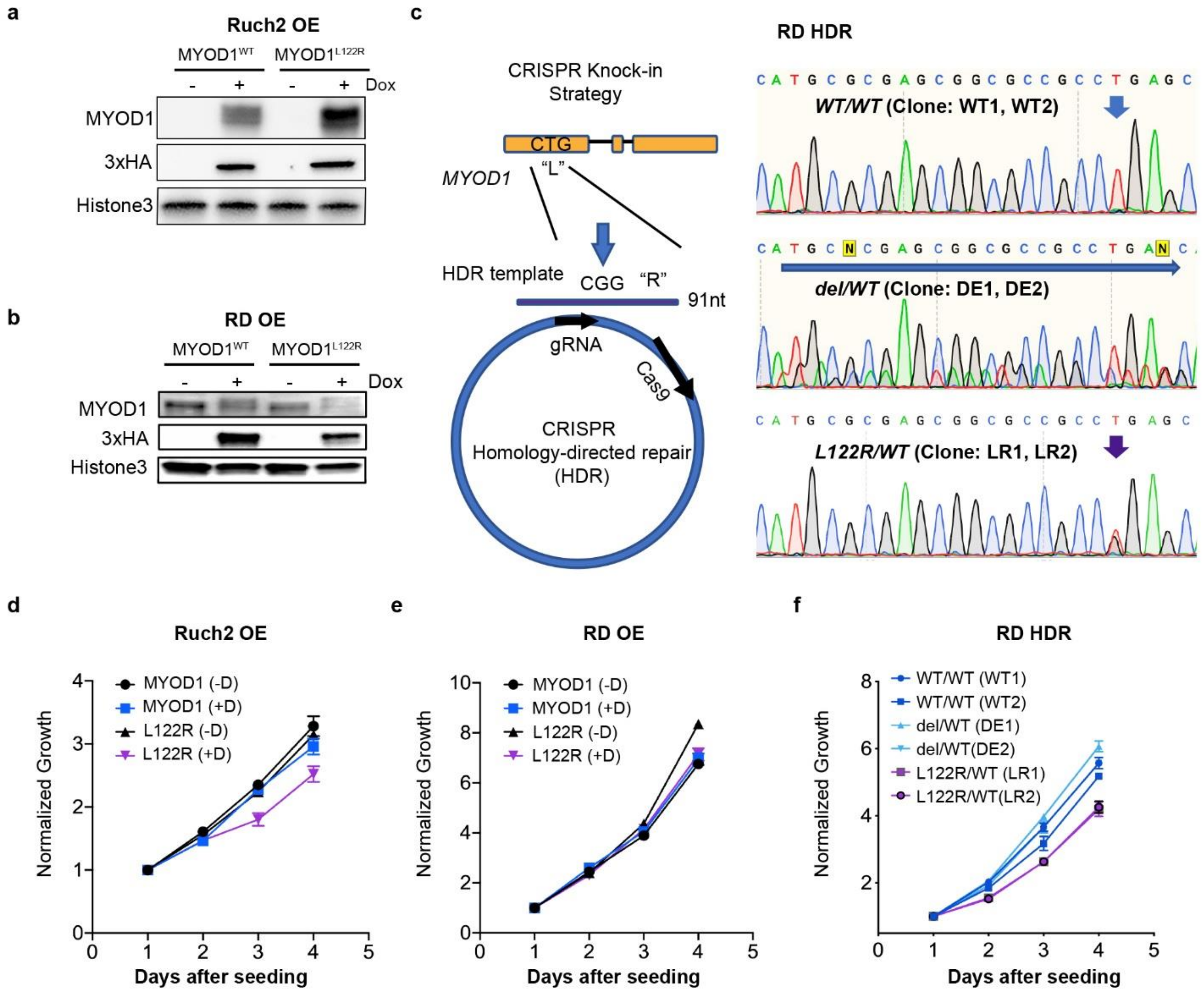

Supplementary Figure 2. Generation of engineered, isogenic human cell line models. **a,b**) Western blot analysis validating overexpression (OE) of 3xHA tagged proteins before and after 72 hours of doxycycline-treatment (Ruch2, a and RD, b). Same blots are rendered from Figure 1a and 1b for Western blots validating OE models. CRISPR-knock in approaches using homology-directed-repair (HDR) to create isogenic RD models (left) and representative Sanger sequencing tracks for clones isolated from single cells that contain 1) two wild-type alleles of MYOD1 (WT1, WT2), 2) one allele deletion of MYOD1 (DE1, DE2) and 3) heterozygous mutation of a T to G mutation that creates the MYOD1^{L122R} mutation (see arrow, LR1, LR2). . Representative blots (a-b) shown for three biological replicates with similar results. **d-f**) Engineered cell models grow similarly when assessed by CellTiter-Glo. Doxycycline inducible

models (Ruch2 overexpression, Ruch2 OE), RD overexpression (RD OE) and RD models generated using homology directed repair (RD HDR). Three biological replicates were used for each datapoint in d-f, mean±S.E.M. The experiment was repeated twice with similar results being observed. The samples derive from the same corresponding experiment but different gels for MYOD1, 3xHA, and Histone 3, and were processed in parallel (a and b). Source data are provided as a Source Data file.

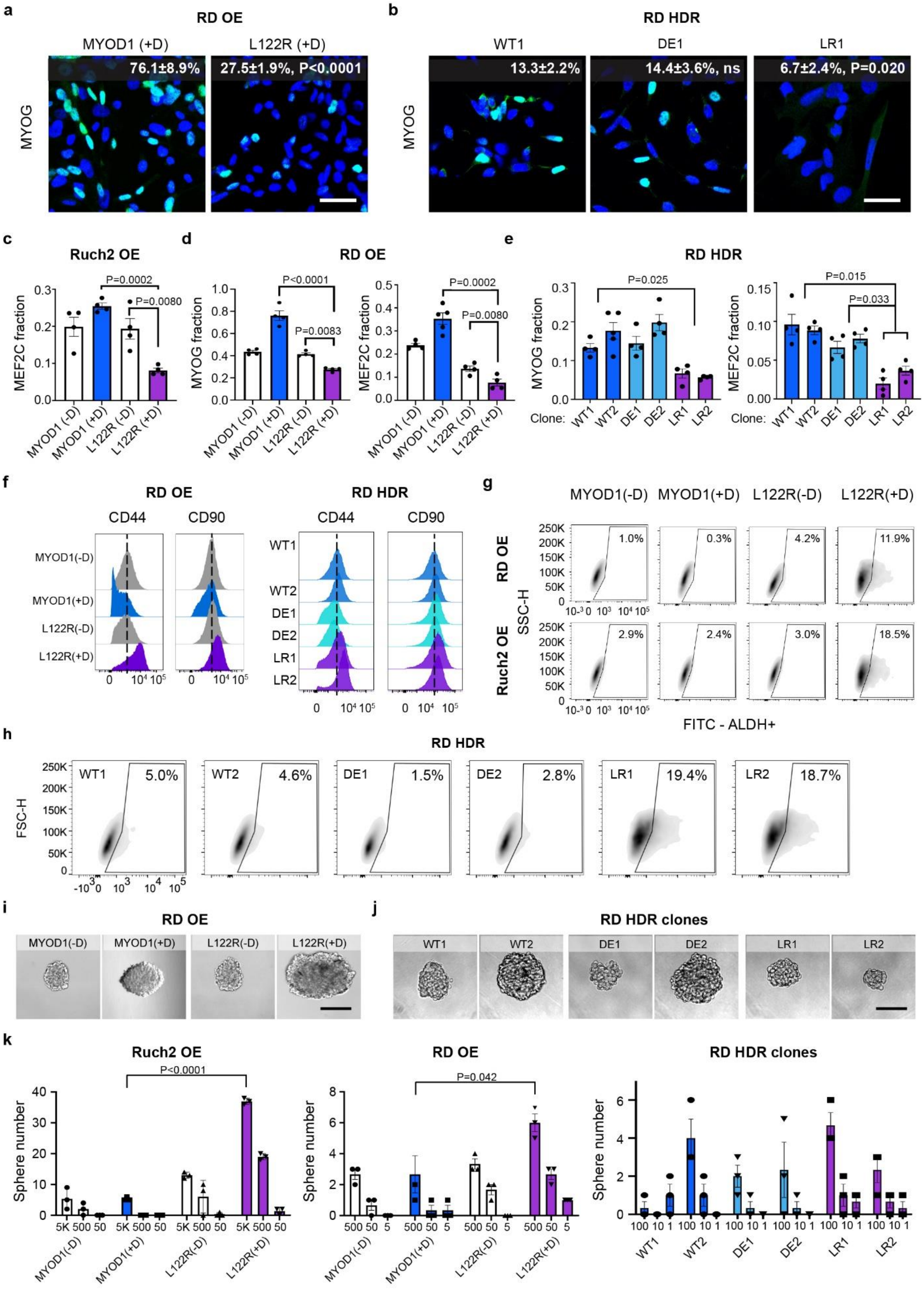

a
RD OE
MYOD1 (+D)
L122R (+D)
76.1±8.9%
27.5±1.9%, P<0.0001
MYOG
b
RD HDR
WT1
DE1
LR1
13.3±2.2%
14.4±3.6%, ns
6.7±2.4%, P=0.020
c
Ruch2 OE
MEF2C fraction
P=0.0002
P=0.0080
d
RD OE
MYOG fraction
P<0.0001
P=0.0083
MEF2C fraction
P=0.0002
P=0.0080
e
RD HDR
MYOG fraction
P=0.025
MEF2C fraction
P=0.015
P=0.033
Clone:
f
RD OE
CD44
CD90
RD HDR
g
MYOD1(-D)
MYOD1(+D)
L122R(-D)
L122R(+D)
RD OE
Ruch2 OE
SSC-H
FITC - ALDH+
h
RD HDR
FSC-H
WT1 5.0%
WT2 4.6%
DE1 1.5%
DE2 2.8%
LR1 19.4%
LR2 18.7%
i
RD OE
j
RD HDR clones
k
Ruch2 OE
P<0.0001
Sphere number
RD OE
P=0.042
RD HDR clones

Supplementary Figure 3. **Engineered, isogenic human cell line models with MYOD1^{L122R} are less differentiated and have overall higher stemness**. **a,b**) Immunofluorescence images and quantification of the numbers of differentiated MYOG+ cells (green) and counterstained with DAPI nuclei stain (blue) in doxycyline-induced models (+D) or representative HDR clones. Mean $\pm$ S.T.D. noted and statistical significance assigned by One-way ANOVA followed by Tukey's multiple comparison. **c-e**) Quantification of fraction of MYOG+ or MEF2C+ differentiated cells across engineered cell models. For immunofluorescence, data obtained from individual fields of view for each condition (n=4-5 biological replicates) with mean$\pm$S.E.M. noted. One-way ANOVA followed by multiple comparison correction was used for statistical analysis. **f**) Flow cytometry analysis for stemness markers CD44 and CD90. **g-h**) Representative flow cytometry quantifying the number of aldehyde dehydrogenase-positive RMS cells ($ALDH^+$, Aldeflour assay) in doxycycline inducible models (g) and RD HDR clones (h). For all flow experiments (f-h), there were three biological replicates and experiment was repeated independently twice. **i-k**) Tumorsphere assays to determine the tumor propagating cell (TPC) frequency in engineered RMS models. Representative images of tumor spheres from RD OE cells (i) and RD HDR clones (j). Quantification of tumorsphere numbers from each dilution condition and experimental group (k). Tumorsphere assay (i-k) was conducted independently twice with similar observed results (n= 3 biological replicates). Not significant (ns). $P < 0.05$ was considered statistically significant. Scale bar equals 20µm (a,b) and 100µm (i, j). Source data are provided as a Source Data file.

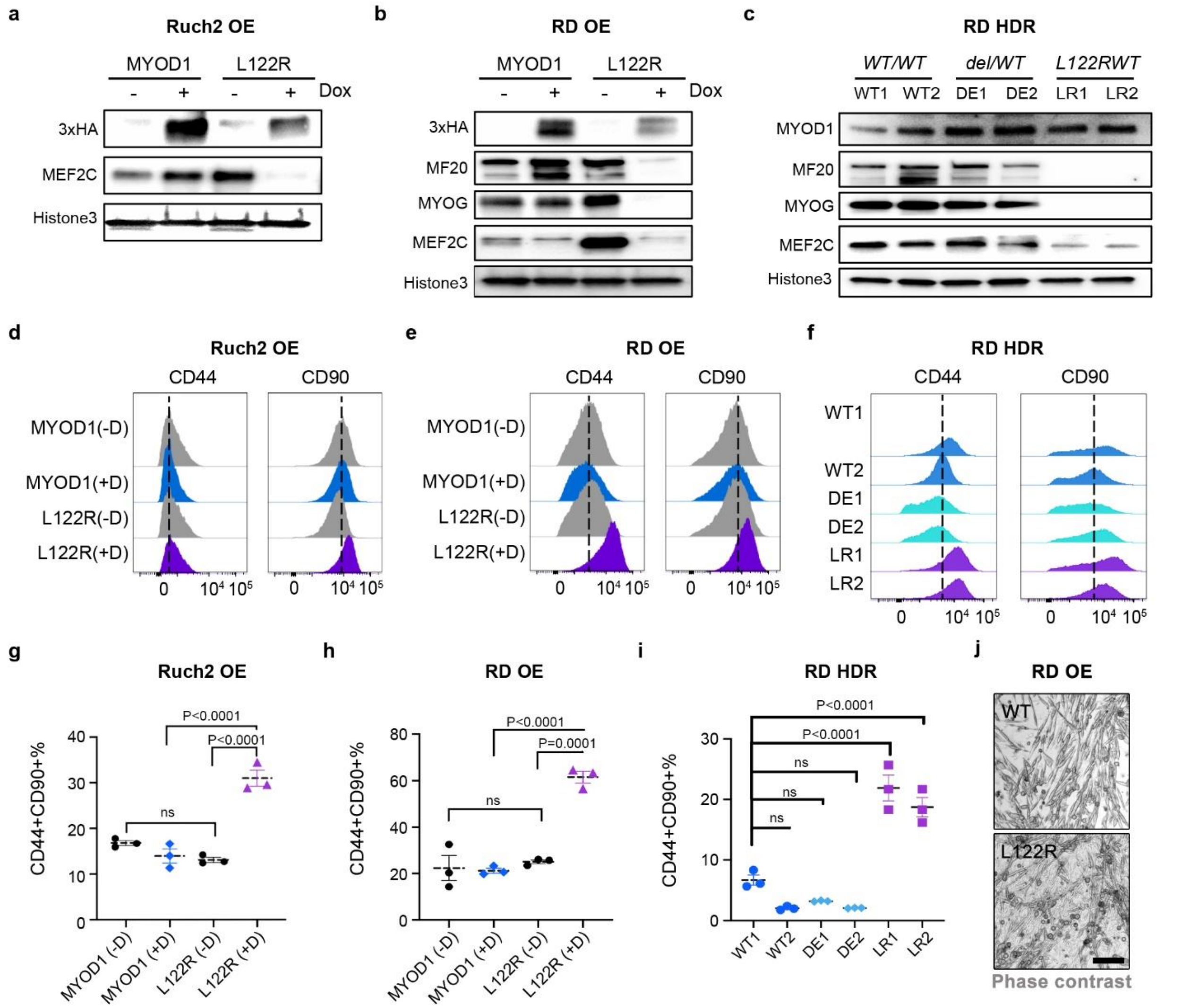

Supplementary Figure 4. *MYOD1^{L122R}* suppresses muscle differentiation and elevates cancer stemness even when grown in differentiation media. Engineered cell line models grown in differentiation medium for 4 days (DMEM-F12 + 2% horse serum). **a-c**) Western blot analysis of doxycycline-inducible (over expression, OE) or CrispR/CAS9 knock-in models generated by homology directed repair (HDR) that express MYOD1 and/or MYOD1^{L122R}. Addition of doxycycline (dox) noted by + signs in a-b. Representative blots (a-c) shown for three biological replicates with similar results. Blots in panels a and b are replicated from Supplementary Figure 2. **d-f**) Flow cytometry analysis for stemness markers CD44 and CD90. **g-i**) Quantification of flow

data. For all flow experiments (d-f), there were three biological replicates and experiment was repeated independently twice. mean±S.E.M. noted. One-way ANOVA followed by Tukey's multiple comparison was performed for statistical analysis. Not significant (ns). $P < 0.05$ was considered statistically significant. **j**) Phase contrast images of engineered cell line models grown in differentiation medium for 7 days. The samples derive from the same corresponding experiment but different gels for 3xHA, MYOG, MF20, MEF2C, and Histone 3, and were processed in parallel (a-c). Source data are provided as a Source Data file.

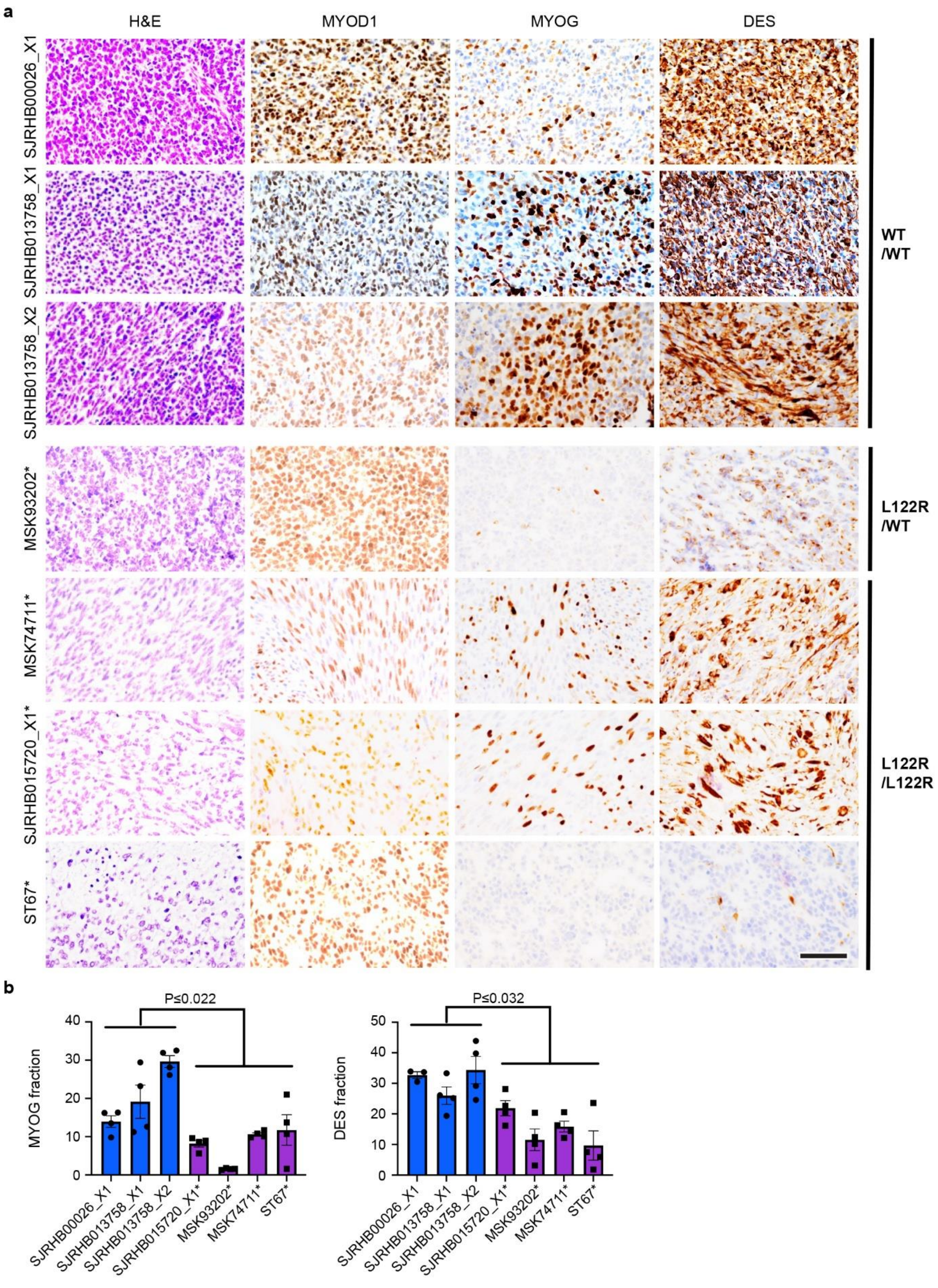

a
H&E
MYOD1
MYOG
DES
SJRHB00026_X1
SJRHB013758_X1
SJRHB013758_X2
MSK93202*
MSK74711*
SJRHB015720_X1*
ST67*
WT /WT
L122R /WT
L122R /L122R
b
P≤0.022
MYOG fraction
P≤0.032
DES fraction
SJRHB00026_X1
SJRHB013758_X1
SJRHB013758_X2
SJRHB015720_X1*
MSK93202*
MSK74711*
ST67*

Supplementary Figure 5. **Patient-derived xenografts (PDXs) with mutant MYOD1^{L122R} contained lower numbers of MYOG+ and Desmin+ cells when compared with FN-RMS PDXs that have wildtype MYOD1. a**) Histopathological analysis of PDX tumors grown in mice. Representative images of hematoxylin and eosin staining (H&E) and immunohistohemistry for MYOD1, MYOG and DES (desmin). Genotype of MYOD shown to right. Scale bar equals 50µM. **b**) Quantification of PDX cells that contain MYOG or DES (n=4 tumors analyzed per PDX model, mean±S.E.M. noted), blue, PDXs with wildtype MYOD1, purple, PDXs with MYOD1^{L122R}. Data was collected from four different fields of view from tumour PDX tissue. Statistics show ANOVA followed by Tukey's multiple comparison. Not significant (ns). $P < 0.05$ was considered statistically significant. Source data are provided as a Source Data file.

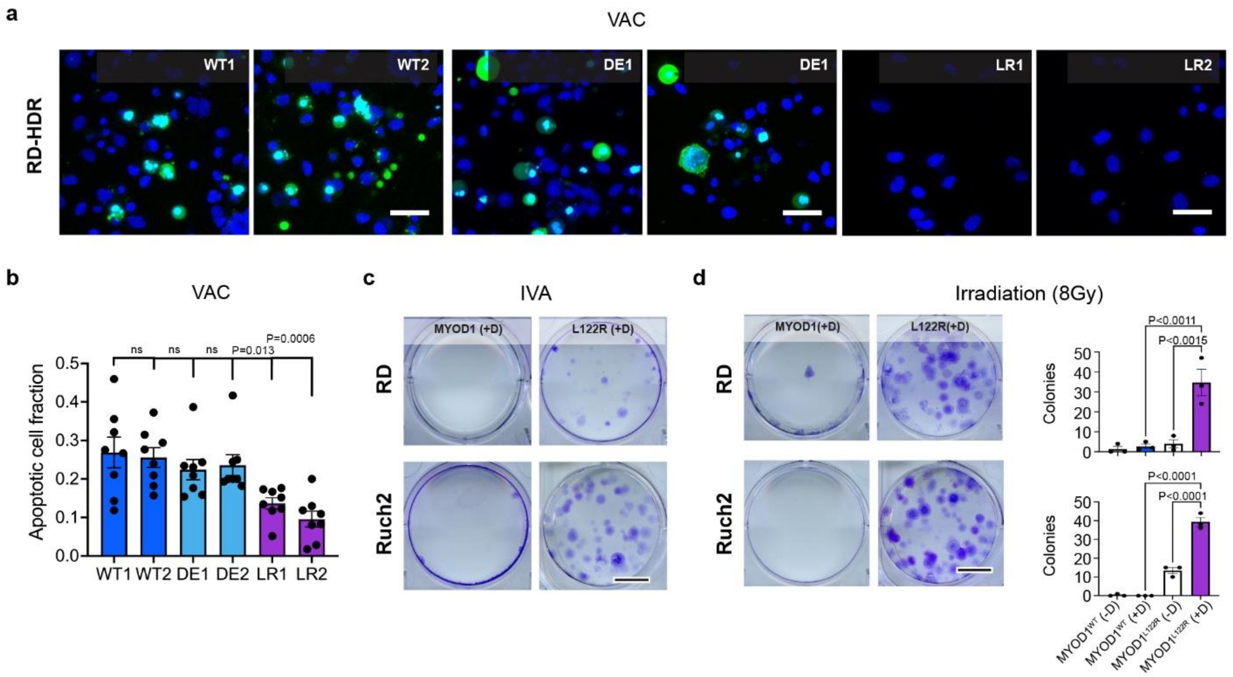

Supplementary Figure 6. ***MYOD1***^{L122R} **induces chemotherapy- and irradiation-resistance. a)** Representative confocal images of engineered cell models treated 4.8µM VAC (vincristine, actinomycin-D and cyclophosphamide) and imaged at 48 hours with the apoptotic reporter Cell Event Caspase-3/7 Green (green) and DAPI nuclei stain (blue, left panels), scale bar equals 20µm, **b**) Quantification. Data obtained from individual fields of view for each condition across multiple wells (n=8 biological reps). Experiment was replicated independently twice with similar results, mean±S.E.M. noted. **c**) Representative image of colonies grown 14 days following treatment with IVA 4.8uM. **d**) Analysis of clonogenic tumor growth following treatment with irradiation (8Gy) and then plated at 300 cells/well in media. Representative images of crystal-violet staining of RMS cells after 21 days, with quantification noted to the right. For clonogenic assays (c, d), there were three biological replicates; similar results were observed twice in independent experiments. mean±S.E.M. noted. One-way ANOVA followed by Tukey's multiple comparison was performed for statistical analysis (b,d). $P < 0.05$ was considered statistically significant. Source data are provided as a Source Data file.

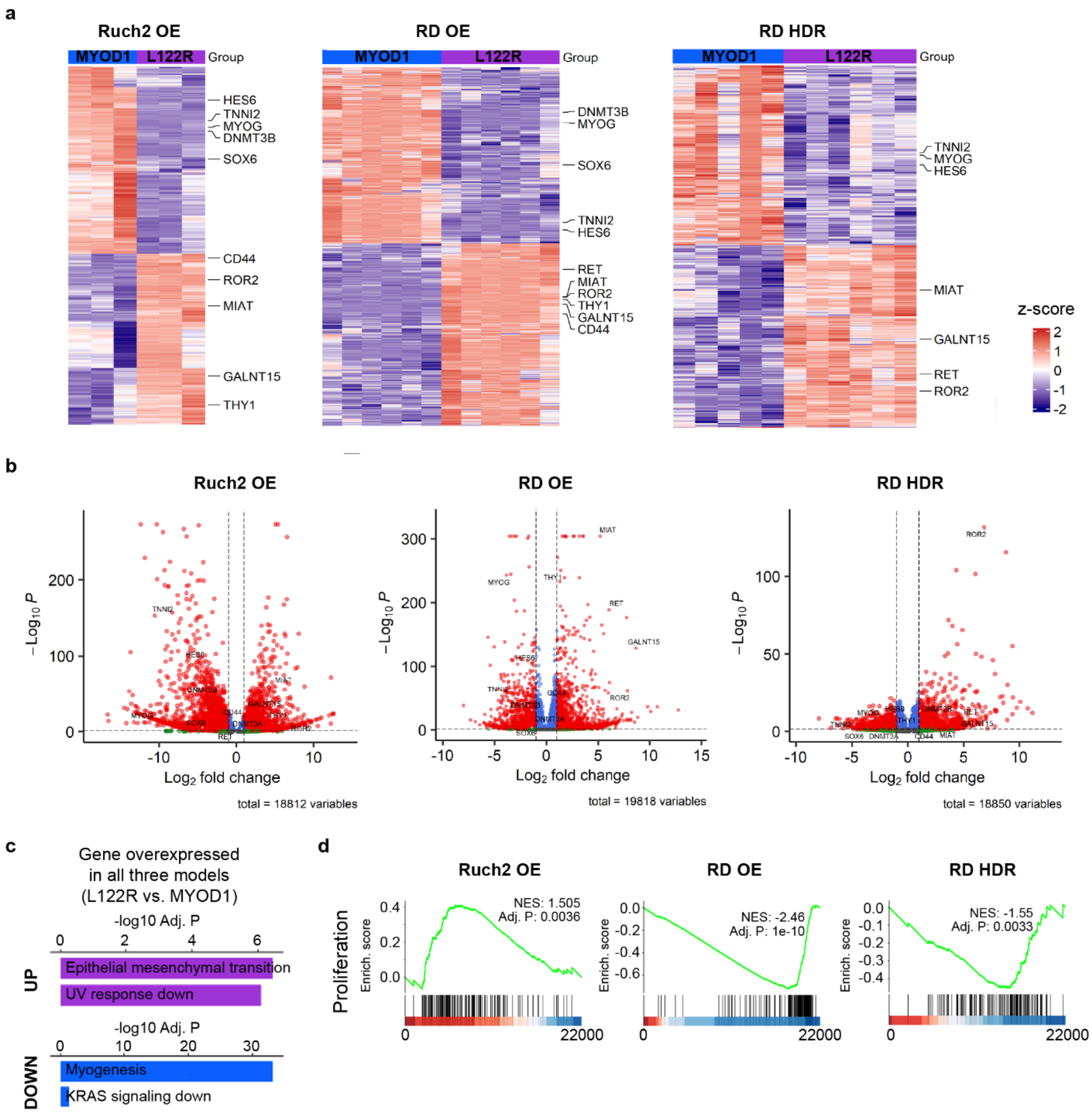

Supplementary Figure 7. **Bulk-RNA sequencing analysis of isogenic, engineered RMS models**. **a**) Heatmaps showing upregulated or downregulated genes in each model (log2 fold>2, *p*.adj.< 0.05). These gene lists were used to generate Venn diagrams shown in in Figure 4a. **b**) Volcano plots showing gene expression in each model. **c**) Over-representation analysis using the high confidence up- and down-regulated gene signatures identified in Figure 4a,b. **d**) GSEA for

the RMS proliferation signature that was defined by the single cell RMS atlas and shows no common expression patterns across models[1].

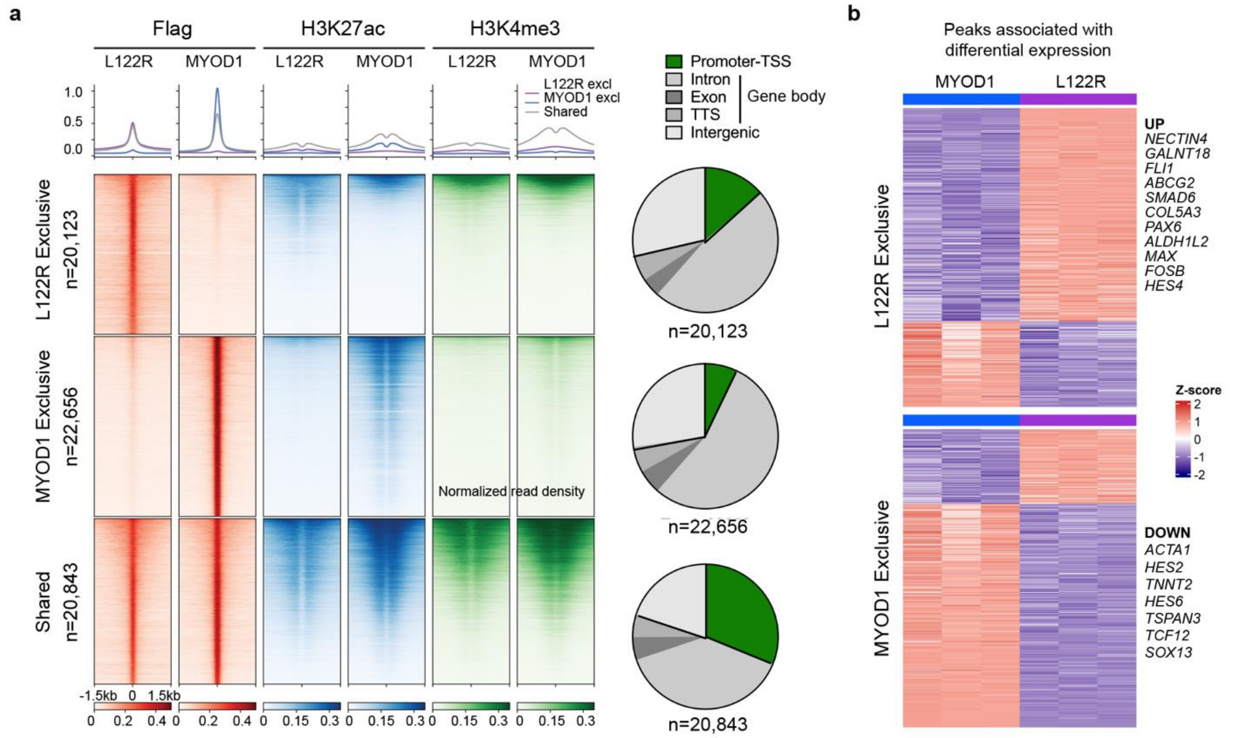

Supplementary Figure 8. MYOD1^{L122R} alters DNA binding specificity, changes histone modification landscape and activates novel gene expression in Ruch2 cells. a) ChIP-Seq from engineered Ruch2 cells that express flag-tagged MYOD1 or MYOD1^{L122R}. Tornado plots comparing Flag, H3K27ac, and H3K4me3 binding across the genome (left). Pie charts detailing the class of genomic regions occupied exclusively by MYOD1^{L122R} (top, right), MYOD1 (middle, right), and co-bound by both (bottom, right). Promoter/TSS (transcription start site) is defined as spanning the transcription starting site and/or with -500bp upstream (green shading). **b)** Heat map showing bulk RNA expression of genes predicted to be bound to and presumably regulated by MYOD1^{L122R} and/or MYOD1. Source data are provided as a Source Data file.

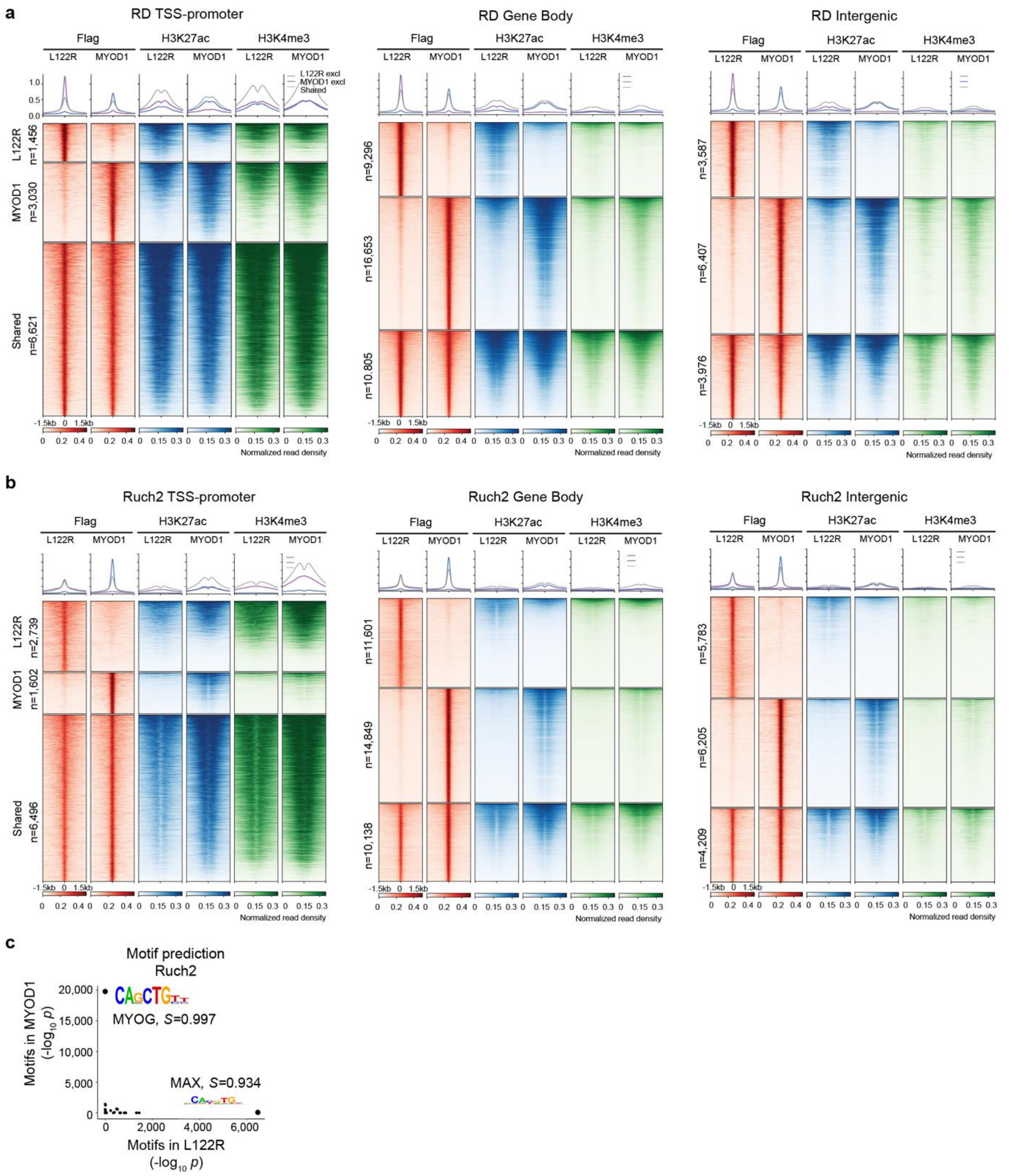

Supplementary Figure 9. **MYOD1^{L122R} alters the histone modification landscape of isogenic engineered RMS models. a,b)** Tornado plots comparing Flag, H3K27ac, and H3K4me3 binding across the genome for RD (a) and RUCH2 (b) engineered models. Classes of genomic DNA elements are demarcated. **c**) Graph denoting differences in binding affinity for MYOD1 verses

MYOD1^{L122R} based on analysis of exclusive binding sites in the genome of Ruch2 engineered models and *de novo* motif prediction using HOMER. Similarly to top predicted, known motifs are noted and similarity score indicated (S).

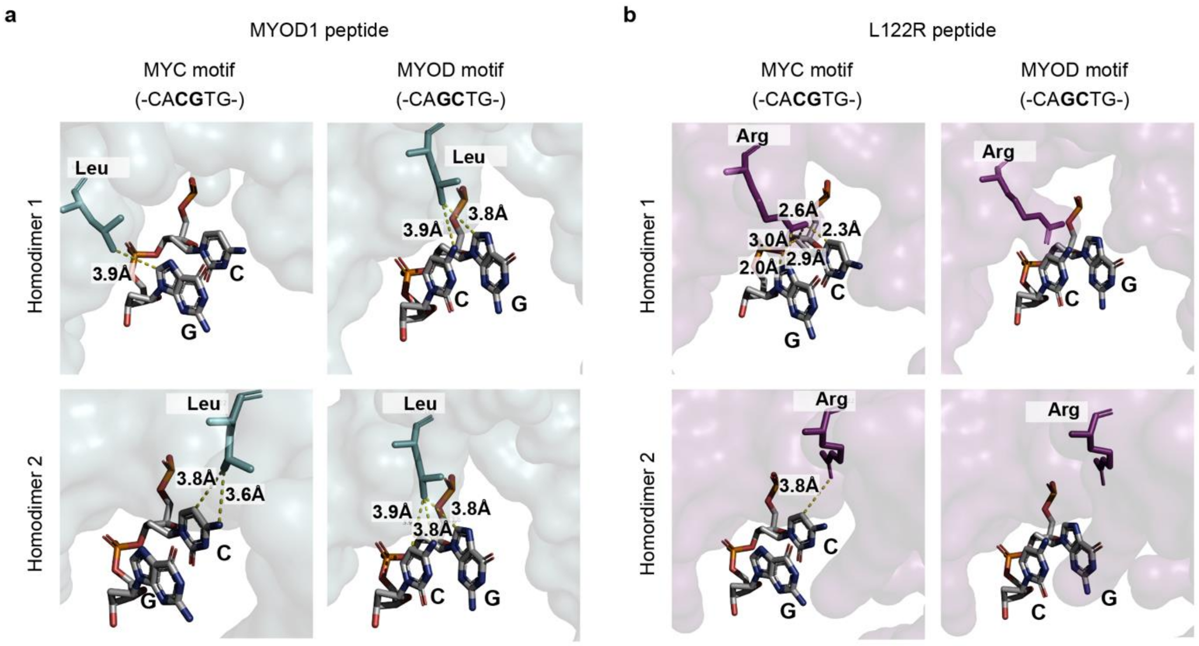

Supplementary Figure 10. AlphaFold2 prediction showing binding of MYOD1 and MYOD1^{L122R} with DNA. AlphaFold2 prediction using the homodimer bHLH regions of wildtype MYOD1 (**a**) or mutant MYOD1^{L122R} (**b**) and simulation of binding to double-stranded DNA: CACGTC (MAX: MYC motif,left) or CAGCTG (the conventional MYOD1/MYOG motif, right). The side chain of the 122Leucine or 122Arginine are noted and predicted interactions between the protein and DNA represented by dotted lines and distance between molecules shown as Ångströms (Å). The Cytosine (C) or Guanine (G) basepairs found in the middle of the palindroms are noted (CA**CG**TC within the MYC:MAX motif or CA**GC**TG within the conventional MYOD1/MYOG motif). Wildtype MYOD1 has high affinity for the conventional MYOD1/MYOG motif (CAGCTG) but still binds MYC:MAX motifs with lower affinity; whereas mutant MYOD1^{L122R} prefers binding with high affinity to MYC:MAX motifs.

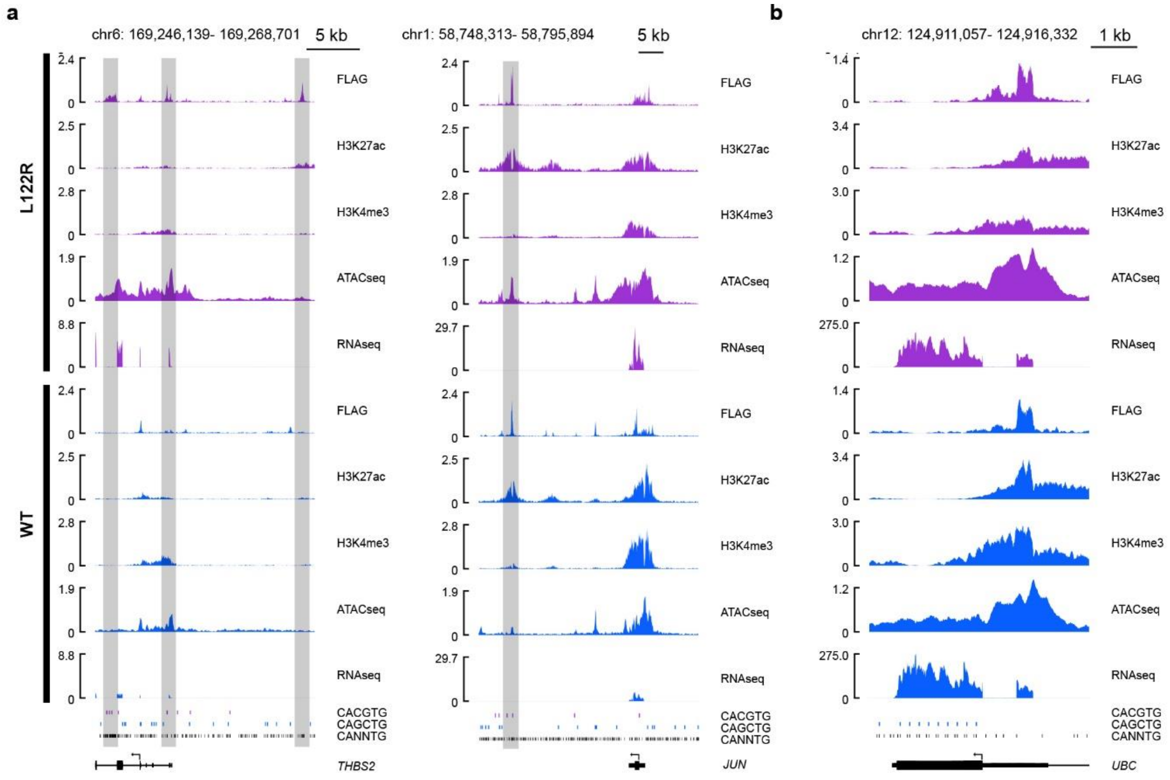

Supplementary Figure 11. Examples of exclusive and shared binding peaks by MYOD1^{L122R} and wildtype MYOD1, along with histone marks. a-b) DNA occupancy map showing the correlation of FLAG, H3K27ac, H3K4me3 in RD cells and open chromatin regions identified by scATAC sequencing of PDX models for *THBS2* and *JUN* that are bound exclusively by MYOD1^{L122R} (a) or *UBC* that are bound both by MYOD1^{WT} and MYOD1^{L122R} (b). RNAseq tracks are also included. Motifs found in the genomic interval are noted at the bottom (MAX:MYC CACGTG in purple, and MYOG CAGCTG and degenerated E-box: CANNTG in grey).

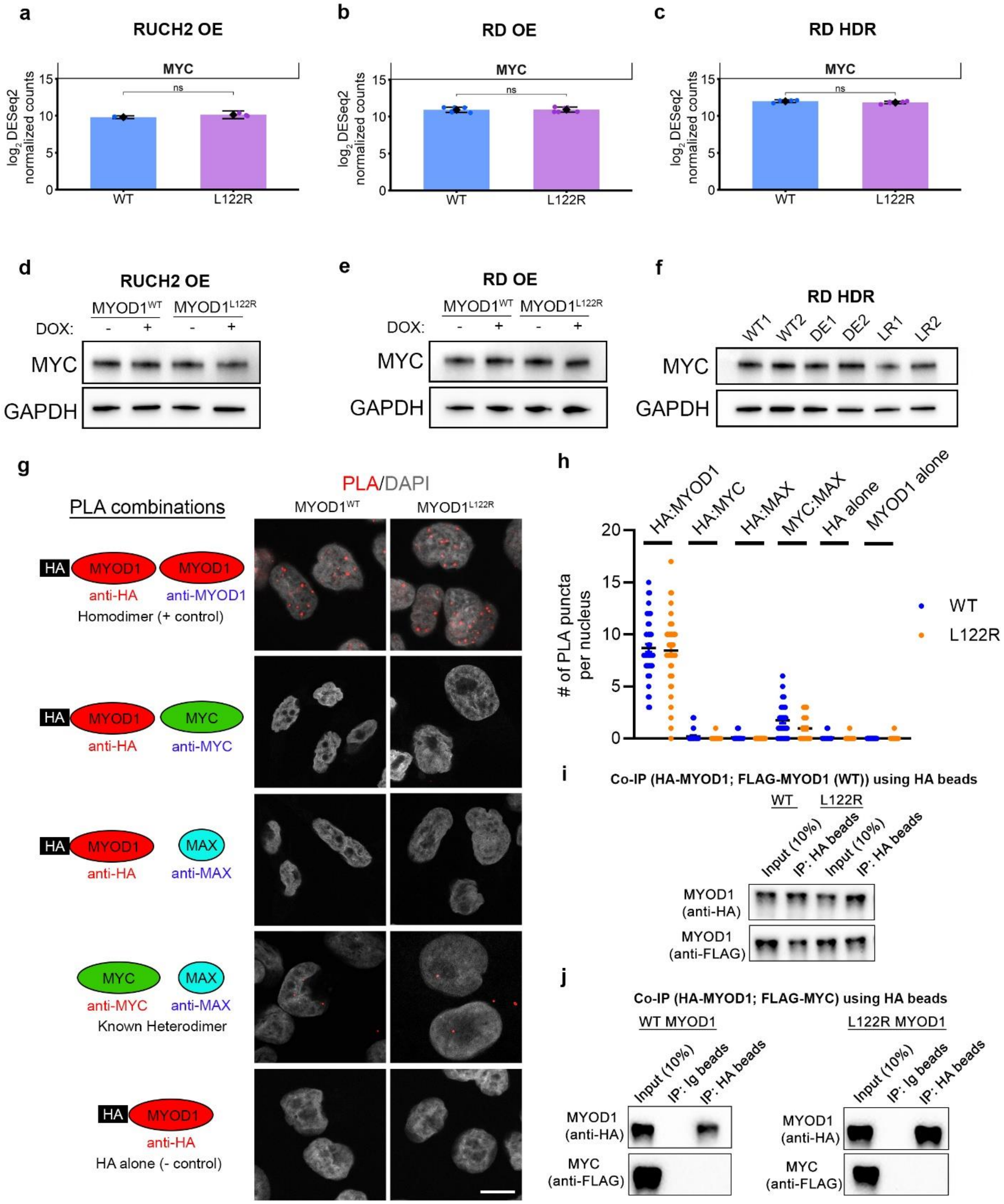

Supplementary Figure 12. cMYC is not differentially regulated by mutant MYOD1^{L122R} nor do they physically interact or bind to similar locations in the genome. **a-c**) Bulk RNA

sequencing analyis confirms that cMYC is not differentially regulated in MYOD1^{L122R} models. **d-f)** Western blot analysis comparing c-MYC expression across models. Representative blots (a-c) shown for two independent experiments where similar results were observed. **g-j**) Proximity-ligation assay shows that c-MYC does not commonly physically interact with MYOD1^{L122R} at locations in the genome. Representative confocal images. Scale bar equals 10µM (g). Quantification of nuclear PLA puncta across experiments (h). **i**) Control co-immunopreciptation experiments confirm MYOD1:MYOD1 homodimerazation and interactions of MYOD1:MYOD1^{L122R}. HEK293T cells were co-transfected with HA-MYOD1 or HA-MYOD1^{L122R} (in the presence of dox for 48 hours) along with FLAG-MYOD1. Immunoprecipitation was performed using anti-HA followed by Western blot analysis. **j**) MYOD1 and MYOD1^{L122R} do not bind with cMYC when assessed in co-immunopreciptation expreiments. HEK293T cells transiently co-transfected with HA-MYOD1 or HA-MYOD1^{L122R} (in the presence of dox for 48 hours) along with FLAG-c-MYC. Immunoprecipitation was performed using anti-HA followed by Western blot analysis. Representative blots (i-j) shown for two independent experiments where similar results were observed. All experiments shown in d-j were replicated three times with similar results. The samples derive from the same corresponding experiment but different gels for MYC, MYOD1 GAPDH, and processed in parallel (d-e, i and j). Source data are provided as a Source Data file.

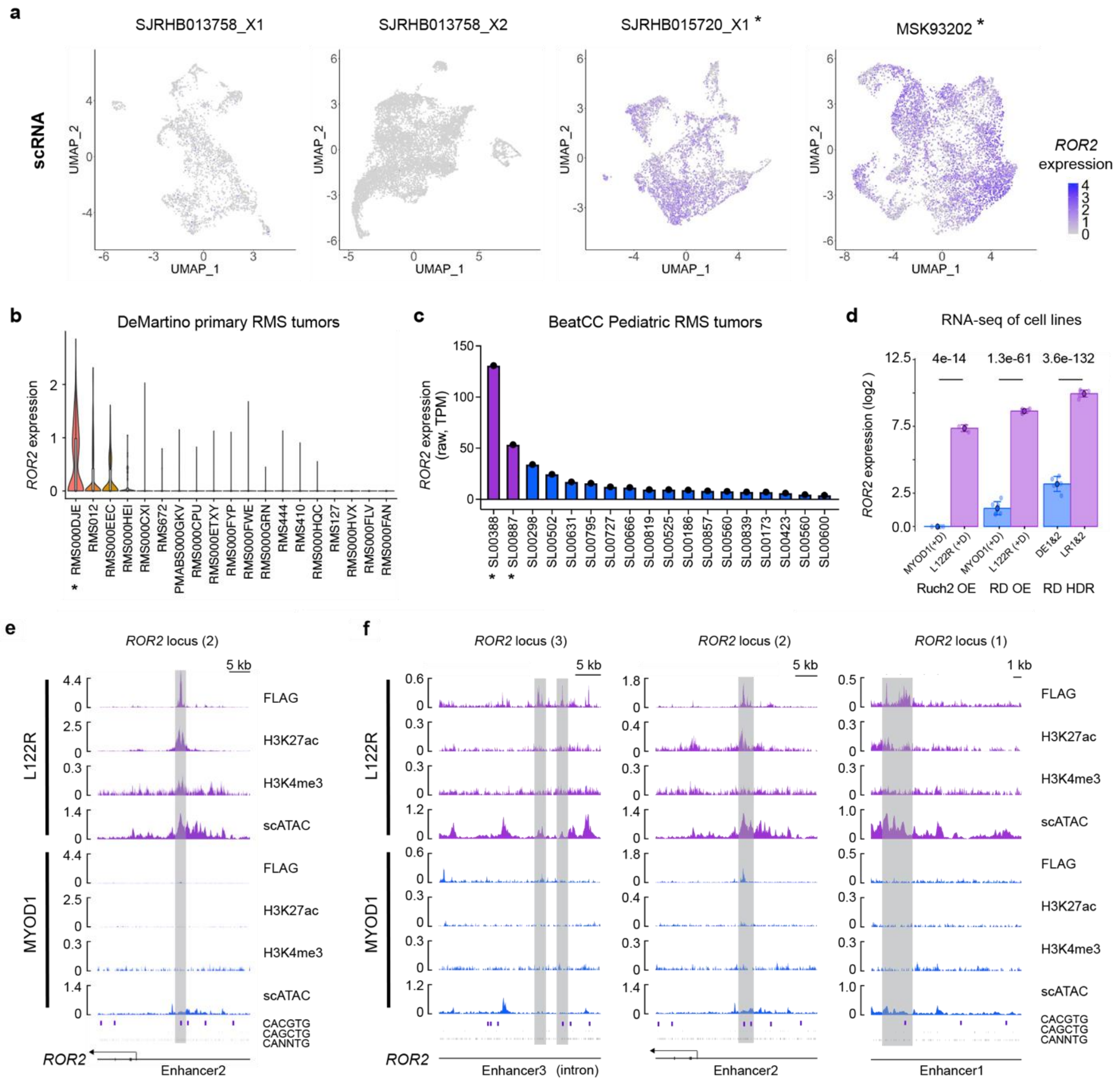

Supplementary Figure 13. *ROR2* is epigenetically and transcriptionally regulated by MYOD1^{L122R}. a) Single-cell RNA-sequencing analysis showing *ROR2* expression in representative FN-RMS that have wild-type MYOD1 (left two panels) and compared with PDX models that contain MYOD1^{L122R} mutations (right two panels). **b)** Single-cell RNA sequencing analysis of 19 RMS patients from DeMartino et al. paper[2] indicated that the patient that has the highest *ROR2* expression harbors a MYOD1^{L122R} mutation. **c)** Bulk RNA sequencing of human RMS obtained from the publicly available NCI cliniomics repository showing that the two samples

with highest *ROR2* expression also harbor MYOD1^{L122R} mutations. Samples with MYOD1^{L122R} mutation are noted by asterisks in panels a-d. **d)** *ROR2* is upregulated in engineered MYOD1^{L122R} cells when assessed by bulk RNA sequencing. Comparison of doxycycline-induced models that express MYOD1^{L122R} or MYOD1 (Ruch2 OE and RD OE) and HDR clones that harbor the MYOD1^{L122R} mutation (LR1-2) or one copy of MYOD1 (DE1-2). Adjusted p values indicated. **f)** DNA occupancy map showing the correlation of FLAG, H3K27ac, H3K4me3 in RD engineered models and juxtaposed with open chromatin regions identified by scATAC sequencing of PDXs. Ooccupancy for enhancers 1 and 3 are shown in main Figure 6B. **g)** DNA occupancy map showing all three potential ROR2 enhancers bound by MYOD1^{L122R} in Ruch2 cells. Motifs found in the genomic intervals are noted at the bottom of panels f and e (MAX:MYC CACGTG in purple, and MYOG CAGCTG in blue, and degenerated E-box: CANNTG, grey). Source data are provided as a Source Data file.

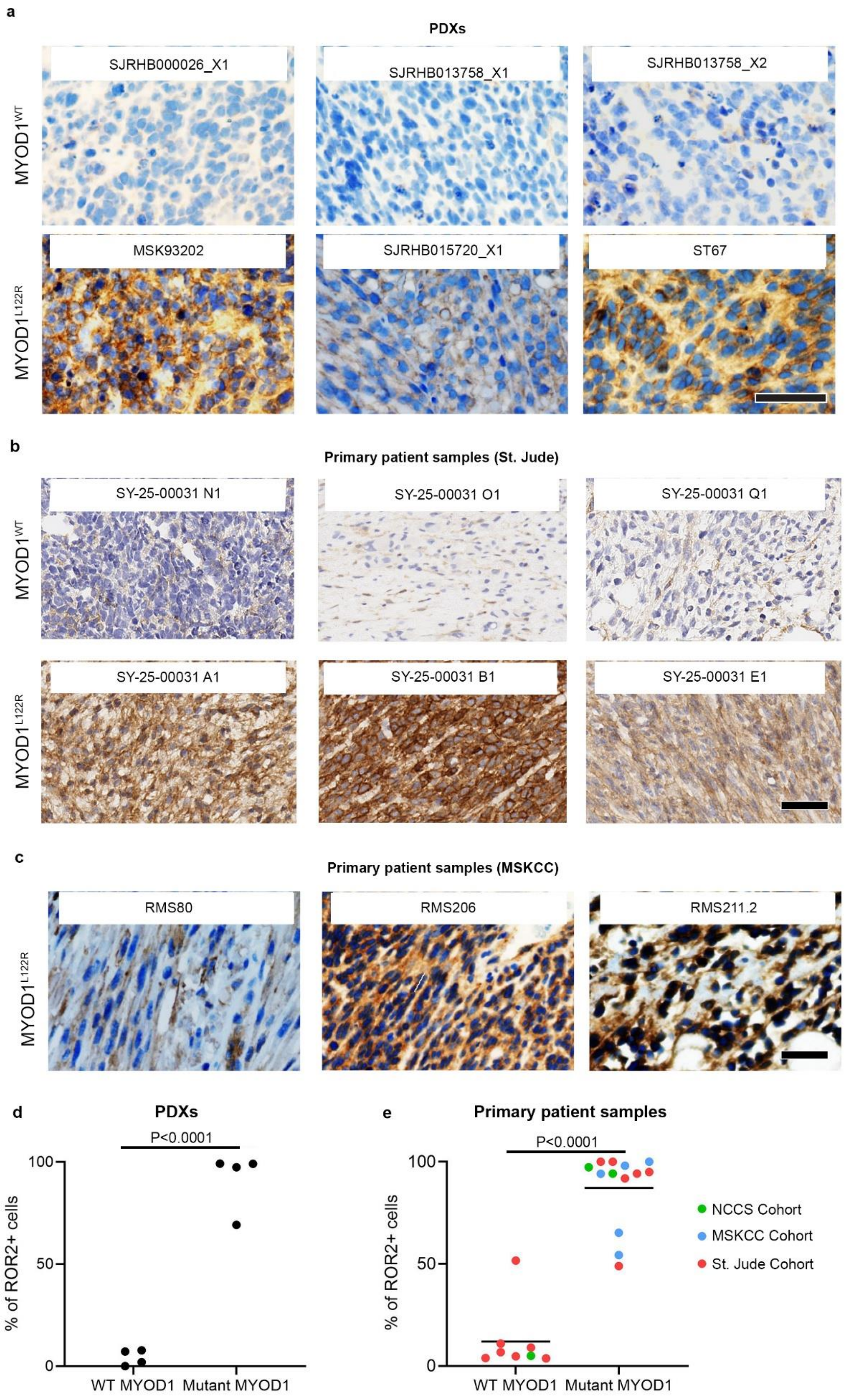
a
PDXs
SJRHB000026_X1
SJRHB013758_X1
SJRHB013758_X2
MYOD1WT
MSK93202
SJRHB015720_X1
ST67
MYOD1L122R
b
Primary patient samples (St. Jude)
SY-25-00031 N1
SY-25-00031 O1
SY-25-00031 Q1
MYOD1WT
SY-25-00031 A1
SY-25-00031 B1
SY-25-00031 E1
MYOD1L122R
c
Primary patient samples (MSKCC)
RMS80
RMS206
RMS211.2
MYOD1L122R
d
PDXs
P<0.0001
% of ROR2+ cells
100
50
0
WT MYOD1
Mutant MYOD1
e
Primary patient samples
P<0.0001
% of ROR2+ cells
100
50
0
WT MYOD1
Mutant MYOD1
NCCS Cohort
MSKCC Cohort
St. Jude Cohort

Supplementary Figure 14. ROR2 is upregulated in MYOD1^{L122R} mutated PDXs and patient samples. a-c) Immunohistolochemistry of ROR2 staining in RMS FFPE samples with either MYOD1^{WT} or MYOD1^{L122R}. Brown indicates ROR2 staining, with nuclei counterstained with hematoxylin (blue). MYOD1 genotype shown to left. Scale bar equals 50μM. Representative images of PDX (a) and patient samples (b-c), **d-e)** Quantification denoting the overall fraction of ROR2-expressing cells when assessed by IHC in PDXs (n=8) (d) and patient samples (n=21) (e). Post-imaging and scanning, three random fields of view were selected and quantified (n>100 tumor cells per sample). Patient samples were from three different cohorts (National Cancer Centre Singapore (NCCS), Memorial Sloan Kettering Cancer Center (MSKCC), and St. Jude Children's Research Hospital (St.Jude). IHC staining was independently done between two institutions by different operators (NCCS and MSKCC samples at MGH and St.Jude samples independently at St.Jude). Student's t-test was performed, and median was shown as lines, . Not significant (ns). $P < 0.05$ was considered statistically significant. Source data are provided as a Source Data file.

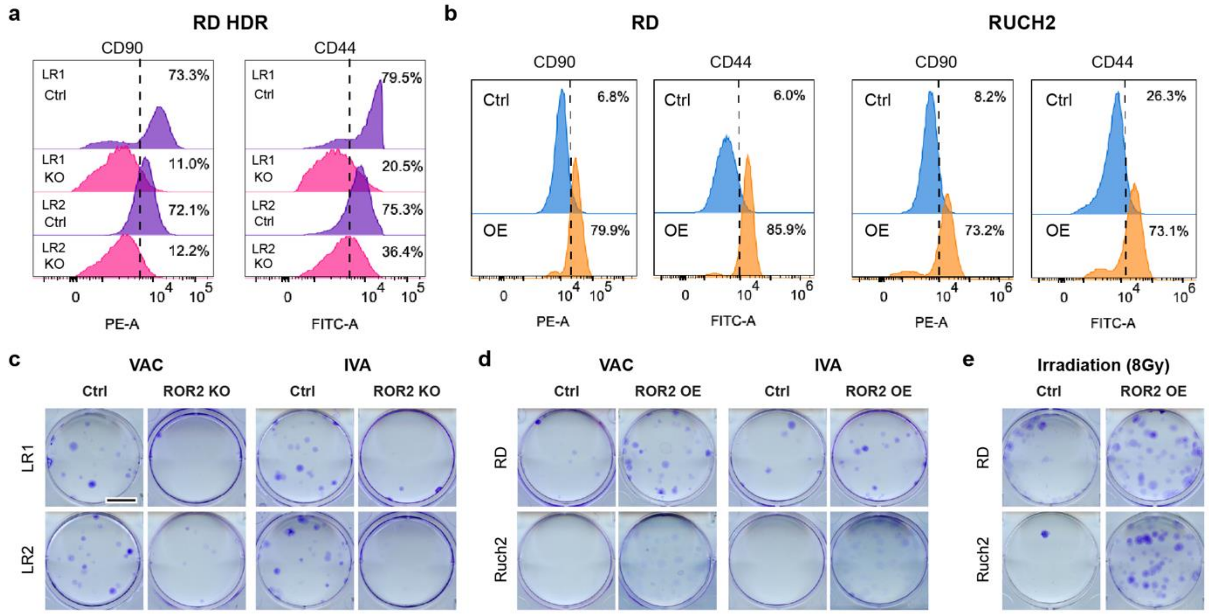

Supplementary Figure 15. **Loss- and gain-of-function experiments confirm that ROR2 positively regulates stemness and therapy resistance**. **a**) Flow cytometric analysis of CD90 (left) and CD44 (right) in engineered MYOD1^{L122R} HDR clones that have intact ROR2 (control, Ctrl) or ROR2 knockout (KO). **b**) Flow cytometric analysis of CD90 (left) and CD44 (right) in RD and RUCH2 cells that overexpress ROR2 (OE). For all flow experiments (a-b), there were three biological replicates and experiment was repeated independently twice. **c-e**) Representative images of clonogenic assays performed post-chemotherapy with VAC or IVA (c, d), or after 8 Gy irradiation (e), scale bar equals 1cm. For clonogenic assays (c, e), there were three biological replicates; similar results were observed twice in independent experiments.

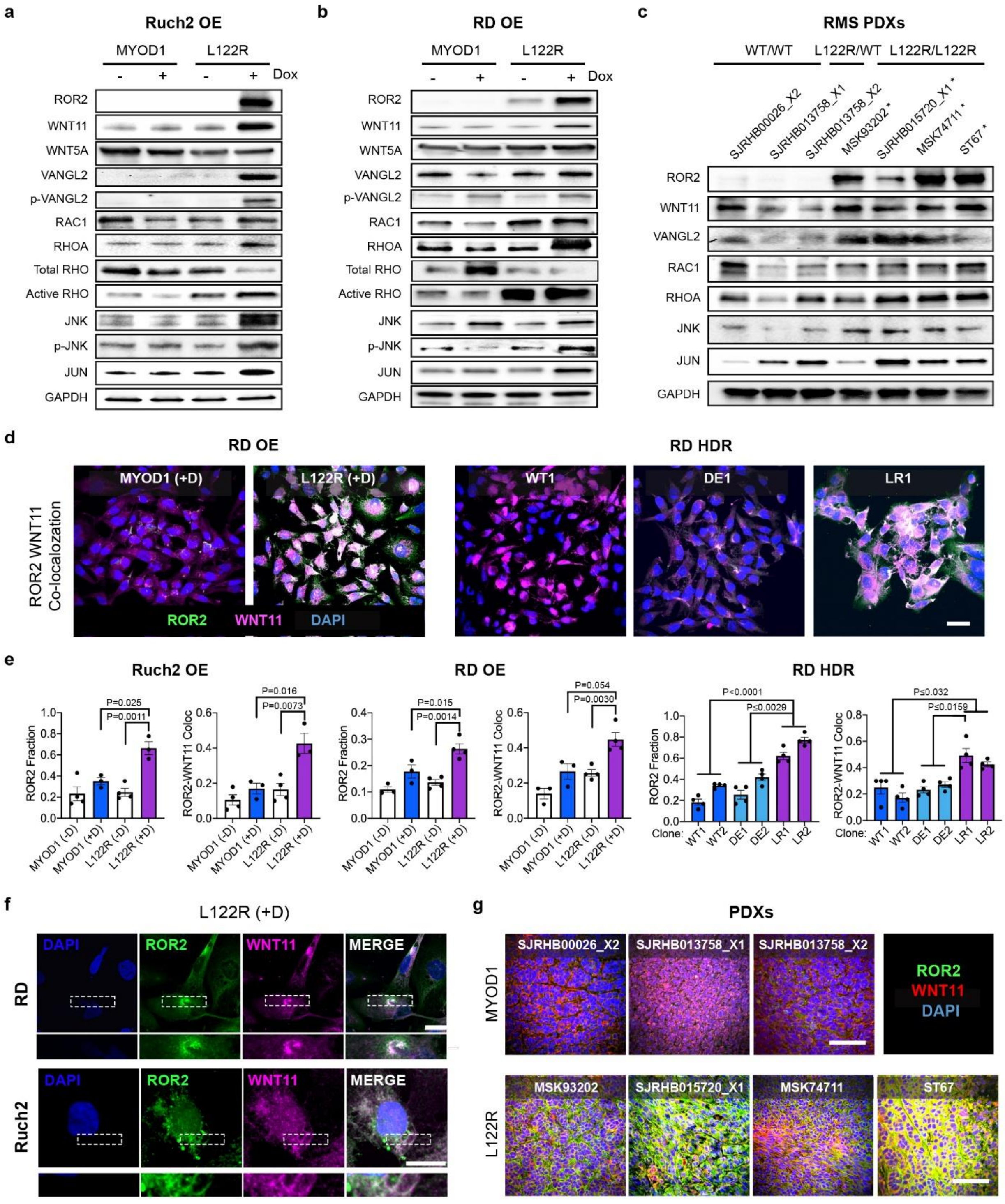

Supplementary Figure 16. MYOD1^{L122R} activates the non-canonical WNT/planar cell polarity pathway through the WNT11-ROR2-VANGL2-RHOA axis. a-b) Western blot analysis showing

expression of non-canonical WNT/planar cell polarity pathway factors in engineered Ruch2 (a) and RD (b) models that have doxycycline-inducible over expression (OE) of either MYOD1 and/or MYOD1^{L122R}, + and – indicates the presence or absence of doxycycline. **c**) Western blot analysis of PDXs with genotype noted. Representative blots (a-c) shown for two independent experiments where similar results were observed. **d**) Confocal images showing immunofluorscence co-localization of WNT11 (purple), ROR2 (green), and DAPI nuclei stain (blue) in engineered models. White notes pixel overlap between WNT11 and ROR2. **e**) Quantification of the fraction of ROR2+ cells and co-localization of WNT11 with ROR2 in all engineered models. For immunofluorescence, data obtained from individual fields of view for each condition (n=4 biological replicates) with mean$\pm$S.E.M. shown. One-way ANOVA followed by Tukey's multiple comparison. Not significant (ns). **f**) High magnification images showing co-localization of WNT11 and ROR2 on the cell membrane of doxycycline-inducible cell line models that express MYOD1^{L122R} (L122R (+D)). White notes pixel overlap between WNT11 and ROR2. **g**) WNT11 and ROR2 are highly expressed in PDXs with MYOD1^{L122R} when assessed by immunofluorescence for WNT11 (purple), ROR2 (green), and DAPI nuclei stain (blue). Scale bars equal 10µm in d, 5µm in f, and 50µm in g. The samples derive from the same experiment but different gels for ROR2, WNT11, WNT5A, VANGL2, phospho-VANGL2, RAC1, RHOA, JNK, phospho-JNK, JUN and GAPDH, and were processed in parallel (a-c). Total RHOA/Active RHOA Western blots are derived from pulldown experiments from the same experiment (a-c). Source data are provided as a Source Data file.

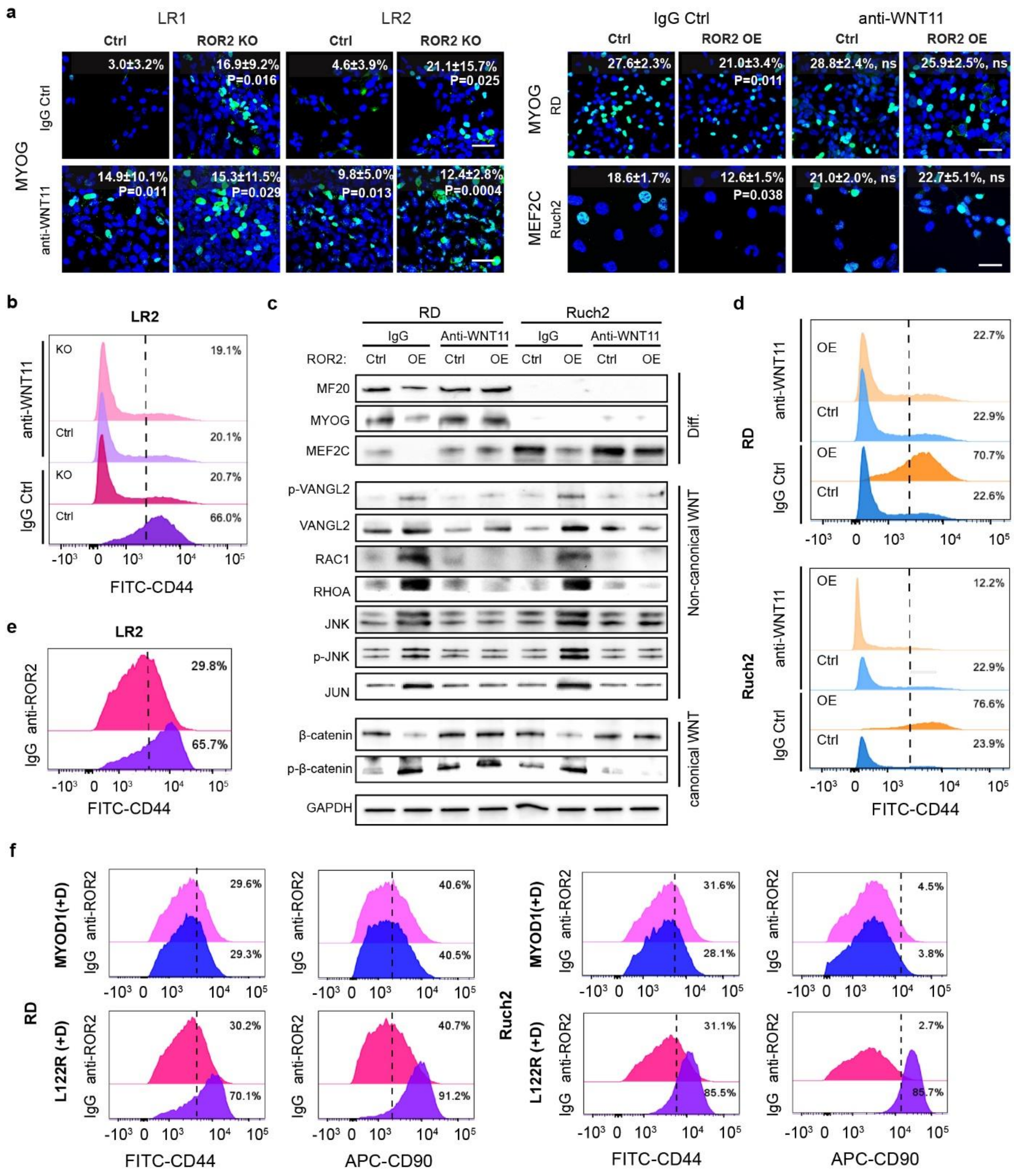

Supplementary Figure 17. WNT11 and ROR2 blocking antibody (anti-WNT11 or anti-ROR2) suppresses expression of the cancer stem cell markers and iduces differentation. a-b) Engineered RD cells with knock-in of MYOD1^{L122R} (LR1 and LR2) and intact or CRISPR/Cas9 depleted ROR2 or RD/RUCH2 ROR2 overexpression models, were treated with control IgG or

anti-WNT blocking antibody and assessed by Immunofluorescence staining of MYOG/MEF2C (a), or flow analysis by CD44-FITC (b). MYOG (green) and DAPI (blue). Also shown are immunofluorescence images noting the mean percentage of MEF2C+ cells +/- S.T.D. MEF2C (green) and DAPI (blue). mean +/- S.T.D. Treatment with IgG or anti-WNT11 antibody noted. A rep-matched one-way ANOVA was performed followed by a Dunnet's test for multiple comparisons. n=4 replicate images analyzed per condition. The experiment was independently replicated twice and similar results were observed. Scale bar equals 20 µm. **b**) flow cytometry for stemness marker CD44 for representative LR1. **c**) Western blot of parental RD and Ruch2 cells (Ctrl) and those that inducibly express ROR2 (overexpression, OE). Treatment included incubation of cells with IgG control antibody or anti-WNT11 blocking antibody. Representative blots (a-c) shown for two independent experiments where similar results were observed. **d**) Flow analysis of stem cell marker CD44 in CRISPR knockin RD cells with MYOD1^{L122R} (LR2) with IgG control or antibody blocking WNT11. **e**) Flow cytometric analysis of CD44 cancer stem cell marker analyzed in CRISPR engineered RD with MYOD1^{L22R} exposed to IgG control or antibody blocking ROR2. **f**) Flow cytometric analysis of CD44 cancer stem cell marker analyzed in engineered RD-OE (left) and Ruch2-OE (right) models that express either MYOD1^{WT} or MYOD1^{L22R} and exposed to IgG control or antibody blocking ROR2. For all flow experiments (b, d-f), there were three biological replicates and experiment was repeated independently twice. Not significant (ns). $P < 0.05$ was considered statistically significant. The samples derive from the same experiment but different gels for ROR2, WNT11, WNT5A, VANGL2, phospho-VANGL2, RAC1, RHOA, MYOG, MF20, MEF2C, JNK, phospho-JNK, JUN, beta-catenin, phospho-beta catenin, and GAPDH, and were processed in parallel (c). Source data are provided as a Source Data file.

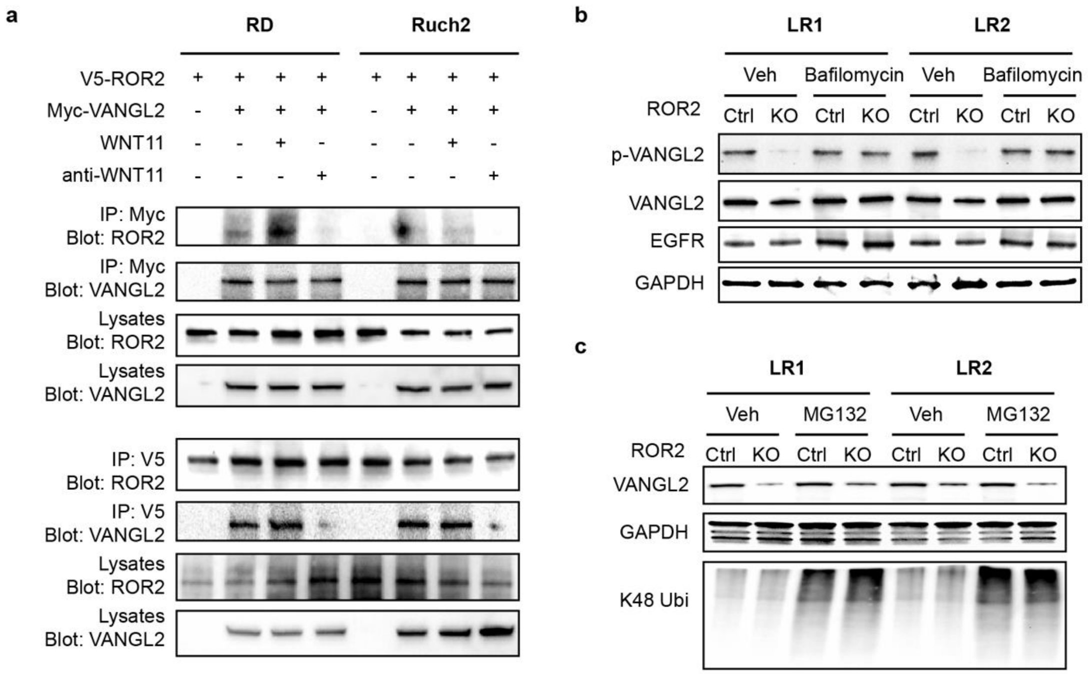

Supplementary Figure 18. ROR2 interacts with and can be stablized by VANGL2 on the cell membrane through direct binding. **a**) Co-immunoprecipitation shows that VANGL2 and ROR2 bind to each other and that this interaction can be inhibited by anti-WNT11 blocking antibody. **b-c**) Western blot analysis comparing MYOD1^{L122R} knockin models that have intact ROR2 signaling (control, Ctrl) or ROR2 knockout out (KO) treated with vehicle or inhibitors for endocytosis and lysosome degradation (bafilomycin), or inhibitors of proteosome degradation (MG132). Representative blots shown for two biological replicates with similar results (a-c). The samples derive from the same corresponding experiment but different gels for ROR2, VANGL2 blots which were from Co-IP pulldown material or lysate material for the same experiment and were processed in parallel (a). The samples derive from the same corresponding experiment but different gels for ROR2, phospho-VANGL2, EGFR, K48 ubiquitin and GAPDH, and were processed in parallel (b and c).

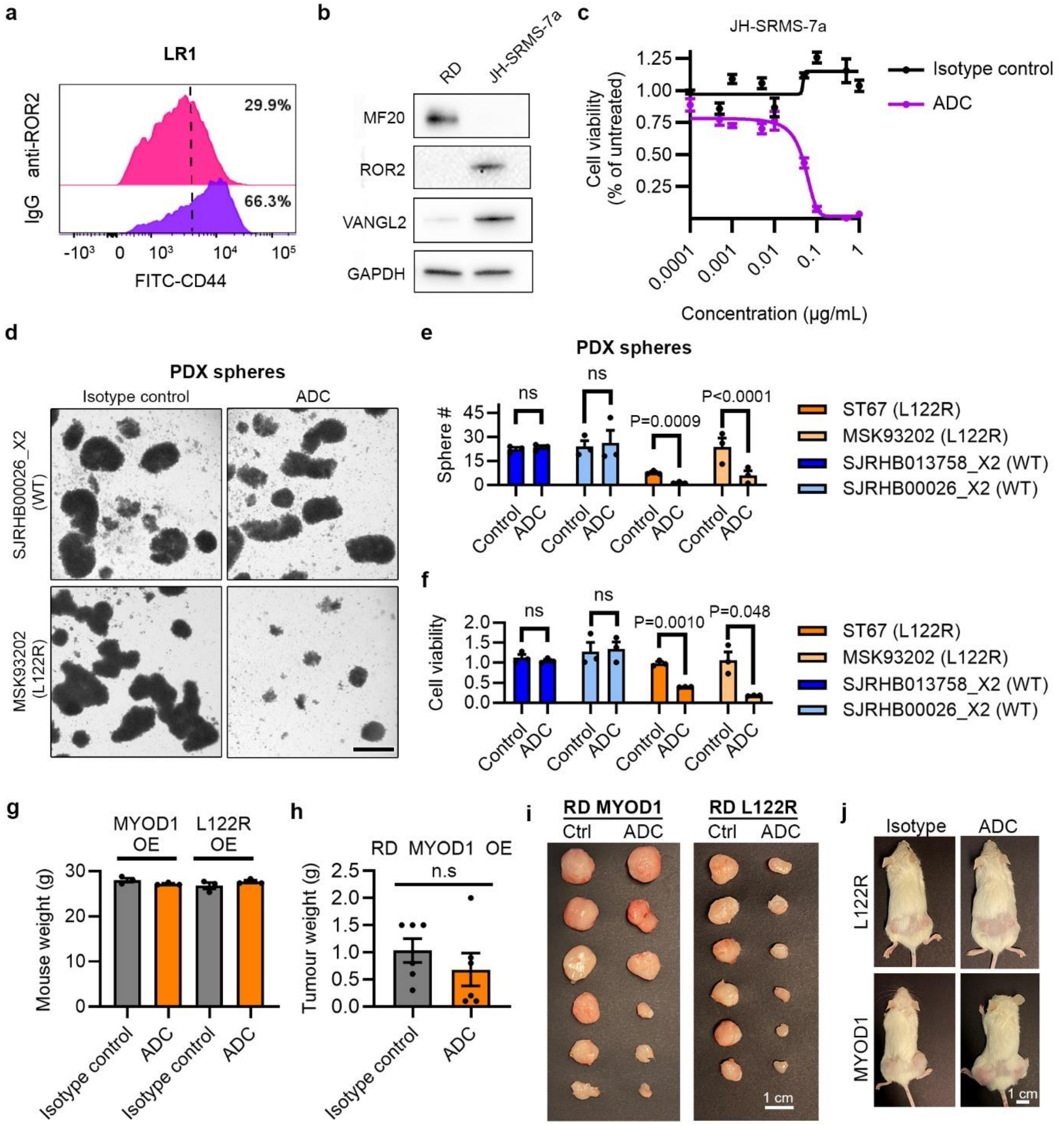

Supplementary Figure 19. ROR2 can be therapeutically targeted in MYOD1^{L122R} mutated RMS. a) Treatment of RD HDR clone LR1 with anti-ROR2 for 4 days reduces the fraction of CD44+ progenitors. **b**) Western blot analysis of patient derived MYOD1^{L122R} mutated SS-RMS cell line (JH-SRMS-7a). Representative blots (a-c) shown for two independent experiments where similar results were observed. **c)** JH-SRMS-7a is efficiently killed after 4 days of treatment with Ozuriftamab vedotin ADC (ADC) while isotype control antibody had no effect on viability when

assessed by Cell-Titer Glo. Statistical significance was determined using a Two-Way ANOVA followed by Šídák's multiple comparisons test comparing doxycycline-treated MYOD1 and MYOD1^{L122R} cells. mean±STD. **d-f**) PDXs with mutant MYOD1^{L122R} are sensitive to Ozuriftamab vedotin when grown *ex vivo* as 3D spheres. PDXs were grown as spheres for two weeks and then treated with 0.05 µg/mL of ADC for 7 days. Quantification of sphere number (e) and cell viability after disassociation using Cell-Titer Glo (f), mean±S.E.M. noted. Tumor sphere viability assays were conducted independently twice with similar observed results (n=3 biological replicates for sphere and CellTitre-Glo). Student's t-test was performed for pairwise comparison between Isotype control and ADC for each PDX. Not significant (ns). $P < 0.05$ was considered statistically significant. **g**) Mouse weight at the end of xenograft experiments and following excision of tumor. **h**) Tumor weight as assessed at necropsy. Not significant (ns) by Student-t test (n=6 mice per group, mean±S.E.M. noted). **i**) Images of mouse xenograft tumours at necropsy. **j**) Images of mice at the end of the experiment carrying mutant MYOD1 engrafted and WT MYOD1 engrafted tumours for each treatment arm. The samples derive from the same experiment but different gels for MF20, ROR2, VANGL2, and GAPDH, and were processed in parallel (b). Source data are provided as a Source Data file.

Supplementary Table 1. MYOD1^{L122R} alone is not oncogenic in zebrafish models.

Microinjected plasmids	# fish followed	RFP+ fish @15 dpf	Tumors atfer 2 years
rag2:MYODL122R-tdTomato	70	15	0
mcad:MYODL122R-tdTomato	80	17	0
myog:MYODL122R-tdTomato	68	19	0
mylz2:MYODL122R-tdTomato	25	15	0

days post fertilization (dpf)

Supplementary Table 2. Zebrafish with MYOD1^{L122R} tumors showed lower median survival.

	G12D	G12D+MYOD1	G12D+L122R
**Median survival (dpf)**	39.5	56.5	28

days post fertilization (dpf)

References:

1 Danielli, S. G. *et al.* Single cell transcriptomic profiling identifies tumor-acquired and therapy-resistant cell states in pediatric rhabdomyosarcoma. *Nat Commun* **15**, 6307 (2024). https://doi.org/10.1038/s41467-024-50527-2

2 DeMartino, J. *et al.* Single-cell transcriptomics reveals immune suppression and cell states predictive of patient outcomes in rhabdomyosarcoma. *Nat Commun* **14**, 3074 (2023). https://doi.org/10.1038/s41467-023-38886-8
