# Supplementary material for: MYOD1 mutation drives cancer stem cell pathways and therapy-resistance in spindle cell/sclerosing rhabdomyosarcoma
Source: Nat Commun. 2026 Jun 3;17:7129. doi: 10.1038/s41467-026-73546-7 (PMC13396186; doi:10.1038/s41467-026-73546-7)
Supplement: Supplementary file 2 — Reporting Summary [file 41467_2026_73546_MOESM2_ESM.pdf]

nature portfolio

Corresponding author(s): David M. Langenau, Esther Rheinbay

Last updated by author(s): Oct 20, 2025

Reporting Summary

Nature Portfolio wishes to improve the reproducibility of the work that we publish. This form provides structure for consistency and transparency in reporting. For further information on Nature Portfolio policies, see our Editorial Policies and the Editorial Policy Checklist.

Statistics

For all statistical analyses, confirm that the following items are present in the figure legend, table legend, main text, or Methods section.

n/a	Confirmed	
☐	☒	The exact sample size (*n*) for each experimental group/condition, given as a discrete number and unit of measurement
☐	☒	A statement on whether measurements were taken from distinct samples or whether the same sample was measured repeatedly
☐	☒	The statistical test(s) used AND whether they are one- or two-sided<br>*Only common tests should be described solely by name; describe more complex techniques in the Methods section.*
☐	☒	A description of all covariates tested
☐	☒	A description of any assumptions or corrections, such as tests of normality and adjustment for multiple comparisons
☐	☒	A full description of the statistical parameters including central tendency (e.g. means) or other basic estimates (e.g. regression coefficient) AND variation (e.g. standard deviation) or associated estimates of uncertainty (e.g. confidence intervals)
☐	☒	For null hypothesis testing, the test statistic (e.g. *F*, *t*, *r*) with confidence intervals, effect sizes, degrees of freedom and *P* value noted<br>*Give P values as exact values whenever suitable.*
☐	☒	For Bayesian analysis, information on the choice of priors and Markov chain Monte Carlo settings
☒	☐	For hierarchical and complex designs, identification of the appropriate level for tests and full reporting of outcomes
☒	☐	Estimates of effect sizes (e.g. Cohen's *d*, Pearson's *r*), indicating how they were calculated

Our web collection on statistics for biologists contains articles on many of the points above.

Software and code

Policy information about availability of computer code

Data collection

RNA- and ChIP-sequencing library preparation, quality check, and sequencing
RNA-seq samples were collected from 105 cells using TRIzol (Invitrogen, catalog # 15596026) and then RNA extracted from cells using QIAGEN RNeasy kit according to the manufacture's protocol (QIAGEN, catalog # 74104). Genomic DNA was removed using DNase I (Invitrogen, catalog # 18068015) for 1 hour at 37C. TruSeq Stranded mRNA Library Prep kit (96 samples) was used to build mRNA libraries (Illumina # 20020595) and ChIP and Library prep Kits (Catalog #9005) from Cell Signaling Technology were used to prepare ChIP libraries, following the manufacture's protocol. ChIP DNA libraries were then indexed with Dual Index 7 Primers and 5 Primers from Multiplex Oligos (CST# 47538) compatible with Illumina. AMPure XP and SPRI Select beads were used to clean up amplified DNA.
Qubit DNA assay was used to determine DNA concentration for libraries built from mRNA, and from ChIP samples. Agilent 2100 Electrophoresis Bioanalyzer was used to check mRNA library size (~260bp for RNA libraries, and ~150-900bp, ladder pattern for ChIP libraries) and quality before sequencing run. NextSeq 2000, along with P3 reagents (100 cycles) was used for the sequencing run (Illumina # 20040559). On average, >20M reads per sample were acquired, covering 75bp from both ends for both RNA- and ChIP-seq libraries.
AlphaFold2 prediction
The amino acid sequence of MYOD1 and its corresponding DNA sequence were obtained from RCSB PDB using code 1MDY. Residues 101-169 were used in these models. DNA sequences used in the WT and CG swap models were TCA ACA GCT GTT GA and TCA ACA CGT GTT GA, respectively. The amino acid sequences were entered into the Colab notebook version of AlphaFold v2.3.2 with the link https://colab.research.google.com/github/deepmind/alphafold/blob/main/notebooks/AlphaFold.ipynb, and Github codes https://github.com/sokrypton/ColabFold. Generated models were validated using PROCHECK via the UCLA-DOE lab server86, https://saves.mbi.ucla.edu/. DNA models were built using PyMOL 2.5.4. Four different MyoD-DNA complex models were generated using the Huang Laboratory's HDOCK server, with the link http://hdock.phys.hust.edu.cn/. MYOD-DNA complex surface models were rendered using the PyMOL rendering plugin (https://pymolwiki.org/index.php/Rendering_plugin). The models were analyzed, animated, and imaged using PyMOL 2.5.4.

Data analysis

Analysis of RNA sequencing

Initial FASTQ files were quality evaluated using FASTQC (v. 0.11.9). And all data were summarized using the ngsReports package (v. 2.0.3) in R (v. 4.2.2). Raw reads were trimmed based on quality and the presence of adapters using the trimmomatic tool (v. 0.39). Thus, for the elimination of the sequences of the adapters, the palindromic mode was used, considering a maximum mismatch of two nucleotides with a palindrome clip score threshold of 30 and a simple clip score threshold of 10. Additionally, quality trimming was performed using a window of 4 bp and an average quality score of 20. Very low-quality bases (Phred < 3) were also removed from the beginning and the end of the reads. Reads with a length of less than 18 nucleotides were discarded. The surviving paired reads were aligned with the STAR aligner (v. 2.7.10b) against the human genome (GRCh38 version, Ensembl) using as gene reference a gene transfer format (gtf) file from Ensembl (v. 107). No filter for a minimum number of matched bases required for alignment (--outFilterScoreMinOverLread and --outFilterMatchNminOverLread were set to 0), so all alignments were included in the final BAM file. Read counting at the gene level was carried out using the htseq tool (v. 1.99.2) in its union mode for a reverse-stranded sequencing protocol. Count data were normalized using the DESeq2 (v. 1.38.3) package58 using the size factor method. Genes with low expression were removed in all analyses (at least five reads in four samples for contrast involving groups of six samples, five reads in three samples for contrast involving groups of five samples). Unsupervised hierarchical principal component analysis (PCA) and dendrogram were performed in R using the FactoMineR (v. 2.10) and factoextra (v.1.0.7) R Package Version 1.0.7. packages. Differential gene expression analysis was performed using DESeq2 through the default Wald test for two group comparisons. Gene expression differences were considered significant when Benjamini-Hochberg-adjusted p-values were below 0.05, and their log2 fold change were > 1 between the MYOD1L122R vs. MYOD1 wildtype conditions. In comparisons involving the parental cells groups, these thresholds were slightly modified, and genes were considered significant if their Benjamini-Hochberg-adjusted p-values were below 0.05 and their |log2 fold change| were over 0.58. Volcano plots obtained after this analysis were carried out with the EnhancedVolcano package (v. 1.16.0) R package version 1.16.0,. Ensembl gene IDs were transformed to HGNC IDs using the org.Hs.eg.db package (v. 3.16.0). Venn diagrams depicted to show the relationship between the significant genes were performed through the ggvenn package (v. 0.1.10)). The heatmap representing the genes common to the three lists was carried out using the ComplexHeatmap (v. 2.18.0). We used the clusterProfiler (v. 4.6.2) package to perform gene set enrichment analysis (GSEA). Human Cancer Hallmarks from the msigdb gene set in its version 7.5.1 (released January 2022) obtained through the msigdbr package (v. 7.5.1) were used as pathway source. A custom database comprising genes involved in differentiation, proliferation, and progenitor genes was also used. Enriched pathways with a Benjamini-Hochberg adjusted p-value below 0.05 were considered significant. GSEA graphs were made using a modified version of the gseaplot2 function of the enrichplot package (v. 1.18.4), R package version 1.22.0,. Ridgeplots for Cancer Hallmarks were made using the ggridges package (v. 0.5.6). Individual gene box plots were generated using ggplot2 (v. 3.5.0).

Analysis of ChIP-sequencing

The quality of the ChIP-seq reads was evaluated using the FASTQC tool. Reads were trimmed for quality and adapters removed using trimgalore (v.0.4.3) (https://www.bioinformatics.babraham.ac.uk/projects/trim_galore/). Cutadapt removes adapter sequences from high-throughput sequencing reads using default settings. Alignment against the human genome (version hg38, Ensembl) was carried out using the BWA-MEM algorithm (v. 0.7.15) and the resulting SAM files were transformed to BAM, sorted by coordinates and indexed using the samtools tool (v.1.4.1). Samtools was also used for the removal of the mitochondrial chromosome and unscaffolded regions. The cDNA-detector (v. 0.1.0) program was used to remove cDNA contamination in the BAM files. Duplicate marking was carried out as the sambamba tool (v. 1.0.1). Low-quality reads were removed with the FilterBAM tool (v. 2) by setting a Phred score of 30 as the minimum alignment quality. The determination of the ChIP-seq peaks for each of the histone marks was carried out with the macs2 (v. 2.0.10) program using the "callpeak" option and input samples as a control. Difficult to map regions were obtained from ENCODE (version GRCh38) and removed using the "subtract" command of bedtools (v. 2.30.0). The differential peaks between MYOD1L122R and MYOD1 for the RD and Ruch2 cell lines FLAG ChIP-seq were obtained from the consensus between two analytical approaches. The first of these approaches was carried out using macs2. First, the average of the fragment sizes was obtained using the "predictd" option on the different samples to be contrasted. Next, the pooling of the samples of the same condition (MYOD1L122R or MYOD1) was carried out with the "callpeak" option, using the average fragment size obtained in the previous step and the input samples as a control. As a result, we obtained the effective sequencing depth for each of the conditions. Finally, the "bdgdiff" option of macs2 was used, specifying both the effective sequencing depth of each group ("--d2" option), the minimum distance to be considered between two significant peaks (60 bases in the case of the FLAG epitope, "-g" option) and the minimum length of the significant peaks (120 bases for FLAG, "-l" option). Peaks that obtained a q-value lower than 0.05 were considered significant. In the second approximation, differential peaks were called using the R package DiffBind (v. 3.8.4) together with edgeR (v. 3.40.2). The MACS2-TMM method was used as a method of normalization of the peaks and the determination of the differential peaks between the different conditions was carried out by the adjustment of quasi-likelihood negative binomial generalized log-linear model. Peaks with a Benjamini-Hochberg-adjusted p-value lower than 0.05 were considered significant. To determine the consensus peaks between the two detection methods, we calculated the intersection of the peaks obtained from the two approaches using the bedtools tool in "intersect" mode. All selected peaks were annotated using the "annotatePeaks" function of HOMER (v.4.10). This approach allowed us to establish three groups of peaks for the FLAG epitope in the two cell lines: the exclusive peaks of MYOD1L122R (selected in the macs2 approach as exclusive of "condition1", and significantly enriched in the DiffBind approach [adj.p < 0.05 and Fold > 0]) the exclusive peaks of MYOD1 (selected in the macs2 approach as exclusive of "condition2", and significantly enriched in the DiffBind approach [adj.p < 0.05 and Fold < 0]) and the peaks shared between the two conditions (selected in the macs2 approach as "common" to the two conditions and not significant in the DiffBind approach [adj.p > 0.05]). Heatmap graphs were depicted following two approximations. In the first, the replicates for the same condition and histone marks were merged using the samtools "merge" option. In the second approach, each replicate was used independently. In both cases, bigwig files were generated using the "bamCoverage" option of the deeptools tool (v.3.1.2)75, with a bin size of 10 bp and applying a counts per million (CPM)-like normalization along with the "–centerReads" and "–ignoreDuplicates" options. The scores for each genomic region were calculated using the "computeMatrix" option also from deeptools, using an upstream-to-downstream distance of 1,500 bp from the center of the interval. Finally, the "plotHeatmap" function was used to make the "tornado" graphs. Graphs focused on peaks located on specific genes, also integrating the scATAC-seq open chromatin regions, were made in R using the trackplot package (v. 1.5.01). The initial motif enrichment analysis of the exclusive peaks of the two conditions and the shared peaks was performed using HOMER in de novo discovery mode using a size of the region for motif finding of 200 bp. In a second step, we specifically tested enrichment of the discovered de novo motifs in shared and exclusive peak sets. This approach enabled us to interrogate the same set of motifs for peaks unique to each condition as well as those common to both, allowing us to assess their enrichment in the studied conditions. The final enrichment results were visualized using a scatter plot through ggplot2 in R. Finally, we proceeded to integrate the three peak groups with the corresponding gene expression data, considering the contrasts between the MYOD1L122R and MYOD1 groups against the parental group. To achieve this, the lists of genes associated with peaks in each group were intersected with the differential expression data only selecting protein-coding genes and removing duplicates. This process considered the log2 fold change and adjusted p-value as thresholds, as mentioned earlier. The

result of this integration was visualized in the form of a stacked plot using ggplot2.

Single-cell RNA-sequencing analysis

Single-cell RNA sequencing (scRNA-seq) raw base call (BCL) files retrieved from Illumina Basespace underwent demultiplexing and conversion into FASTQ files using the BCL Convert software (v2.0.0) provided by Illumina. Subsequently, the 10x Genomics Cell Ranger 7.1.0 pipeline was employed to conduct sequence alignment, basic read quality filtering, and counting of cell barcodes and unique molecular identifiers (UMIs). This process utilized both the human (GRCh38) and mouse (mm10) reference genomes downloaded from 10x Genomics website for the analysis of human patient-derived xenograft (PDX) samples. The pipeline generated outputs consisting of filtered cell barcodes and transcript identifiers, as well as read counts per cell and gene, which were utilized for subsequent downstream analysis.

The scRNA-seq matrices underwent analysis utilizing the Seurat v4 pipeline78. Each sample was subject to individual analysis. Since the PDX samples might contain mouse cells, we excluded the cells having >5% mouse reads for further analysis. Additionally, we selected the cells with the number of features ranging from 1,000 to 8,000, and the maximum allowed fraction of mitochondrial genes per cell was 20%. After the preprocessing step, log normalization was performed, and the top 2000 highly variable genes were identified using method vst with default settings. To avoid the domination of highly expressed genes, we scaled the data while regressing out confounding variables, including the number of UMIs, the number of genes, the percentage of ribosome and mitochondria genes, and a fraction of mouse reads. Next, we performed dimensionality reduction using principal component analysis (PCA). The first 20 principal components (PCs) were chosen to construct the K-nearest neighbor (KNN) graph with default settings. Louvain clustering was performed to identify clusters with resolution 0.5 (resolution=0.5), and clusters were identified for each individual sample. We leveraged Uniform Manifold Approximation and Projection (UMAP) to visualize the scRNA-seq clustering results and meta-data information. To mitigate the possibility of doublets within the dataset, we employed the DoubleFinder algorithm79, utilizing optimized sample-specific pK numbers.

To determine cell identities for each cluster, we first identified differentially expressed genes (DEGs) for each individual cluster from each sample using FindAllMarkers function in the Seurat package with following parameters: only.pos = TRUE, min.pct = 0.25, logfc.threshold = 0.25, test.use = "wilcox". Next, we annotated cell identities by comparing cluster specific DEGs with published canonical marker genes.

Integration of multiple single-cell RNA-sequencing datasets

To merge scRNA-seq data originating from both MYOD1 and MYOD1L122R samples, we aggregated all high-quality cells that met our predetermined quality control standards, as outlined in the single-cell RNA sequencing analysis section. This comprised 4 scRNA-seq datasets from MYOD1 FN-RMS samples and 4 from MYOD1L122R samples (See Supplementary Table 4 for more details). Specifically, we combined raw count matrices utilizing the merge function available in Seurat v478. This resulted in a total of n = 55,500 cells from n = 8 PDX samples.

After merging, log normalization was performed, and the top 2000 highly variable genes were identified using method vst with default settings. Subsequently, the data were scaled, and PCA was applied for dimensionality reduction. Based on the elbow plot, we selected the top n = 30 PCs to construct a KNN graph with 20 nearest neighbors (k.param=20). The Louvain algorithm was then employed to identify clusters, with a resolution parameter set to 0.5 (resolution=0.5). To visualize the cells and clusters on a two-dimensional embedding, we reduced the dimensionality using UMAP.

To mitigate batch effects across diverse samples, we aligned all 8 datasets employing Seurat's integration pipeline, leveraging reciprocal PCA (RPCA) approach for the large datasets. This methodology, briefly outlined in the Seurat integration protocol, entails anchor identification between dataset pairs. Each dataset is projected into the PCA space of the other, and anchor constraints are enforced based on a shared mutual neighborhood criterion. We first conducted normalization and feature selection, retaining n = 2,000 variable features for subsequent integration from each dataset. Next, data scaling PCA was conducted on each individual object. Anchors were identified using the FindIntegrationAnchors function with the following parameters: reduction = "rpca", dims = 1:30. We generated the integrated object by utilizing the IntegrateData function, incorporating the top n = 30 PCs. We then scaled and centered the gene expression data, followed by PCA. We determined top n = 20 PCs for further analysis. A KNN graph was constructed, followed by clustering using the Louvain algorithm with a resolution parameter set to 0.2. UMAP was leveraged for visualization. To identify DEGs within each individual cluster, we utilized FindAllMarkers function available in the Seurat package, employing the following parameters: only.pos = TRUE, min.pct = 0.25, logfc.threshold = 0.25, test.use = "wilcox". We annotated cell identities by comparing cluster specific DEGs with published canonical marker genes and gene set enrichment analysis.

Gene signatures enrichment analysis

AUCell80 was employed to identify cells exhibiting active gene signatures at a single-cell resolution. Briefly, AUCell, a ranking-based method, uses the area under the curve to determine the gene set enrichment in individual cells. Having identified gene signatures from ChIP-seq data, we initially ranked all genes for each cell through the utilization of the AUCell_buildRanking function with default settings. Subsequently, the AUCell_calcAUC function was applied to compute the area under the curve for each gene signature across individual cells, with the top 5% of genes in the ranking being used. Similarly, we utilized the AUCell analytical framework to compute cell-state specific gene signatures scores, including progenitor, proliferative, and differentiated cell states. These AUCell enrichment scores were leveraged for ternary plots. To evaluate the stemness of individual cells, we established a progenitor score following the methodology outlined11.

Single-cell ATAC-sequencing analysis

Single-cell ATAC sequencing (scATAC-seq) raw base call (BCL) files retrieved from Illumina Basespace underwent demultiplexing and conversion into FASTQ files using the BCL Convert software (v2.0.0) provided by Illumina. Subsequently, the 10x Genomics Cell Ranger ATAC 2.1.0 pipeline was employed to preprocess the raw data. This process utilized both the human (GRCh38) and mouse (mm10) reference genomes for the analysis of human PDX samples. The pipeline generated outputs consisting of filtered cell barcodes and peak coordinates, as well as read counts per cell and peak, which were utilized for subsequent downstream analysis.

The scATAC-seq matrices were utilized for downstream analysis using the Signac pipeline81, with each sample analyzed individually. Due to the potential for mouse cell contamination in the PDX samples, features associated with mice were omitted, and any mouse cells identified through the 10x Genomics cellranger-atac pipeline were subsequently excluded. To assess the quality of the scATAC-seq data, various quality control metrics were computed. These metrics included nucleosome signal score, transcriptional start site (TSS) enrichment score, the total number of fragments within peaks, the fraction of fragments within peaks, and the ratio of reads in genomic blacklist regions. Subsequently, the scATAC-seq data underwent a stringent filtering process to retain only high-quality cells. Specifically, cells meeting the following criteria were subjected to further analysis: peak region fragments between 1500 and 10000, a blacklist fraction below 0.05, at least 40% of reads within peaks, nucleosome signal below 2, and TSS enrichment above 2. After QC, normalization was conducted using the RunTFIDF function with default parameters. A subset of the top n% of peaks was selected using the FindTopFeatures function with a minimum cutoff of q0. Dimensionality reduction was then performed via singular value decomposition (SVD) using the RunSVD function. It was observed that the first latent variable from SVD primarily reflected sequencing depth, indicating technical rather than biological variability. Therefore, the first component was excluded from further downstream analysis. To embed the cells into a low-dimensional space, we utilized graph-based clustering and non-linear dimensionality reduction for visualization with the following parameters: reduction = 'lsi', dims = 2:20.

To assess the activity of individual genes within the genome based on their associated chromatin accessibility, we calculated a gene activity matrix for each individual samples using GeneActivity function. Briefly, this function counts the fragments attributed to each cell, which map to regions corresponding to gene coordinates, extending to encompass the 2 kb upstream region. We utilized the gene activity matrix derived from scATAC-seq data and integrated it with the corresponding scRNA-seq data from the sample through cross-modality integration and label transfer approaches. Specifically, we identified anchors between the scATAC-seq dataset and the scRNA-seq dataset using the FindTransferAnchors function with reduction = 'cca'.

Single-cell ATAC-sequencing analysis from published data
To compare the four MYOD1L122R PDX samples with the FN- RMSMYOD1 samples, we utilized four publicly available scATAC-seq datasets (SJRHB010927_X1, SJRHB010928_X1, SJRHB013758_X1, and SJRHB013758_X2) sourced from the St. Jude Childhood Solid Tumor Network7. We retrieved publicly available FASTQ files from GEO accession: GSE174376 and implemented the same 10x Genomics Cell Ranger ATAC 2.1.0 pipeline used for our PDX samples.
Similarly, the cellranger-atac output matrices were subjected to downstream analysis using the Signac pipeline, with each MYOD1 sample analyzed individually. Due to the potential for mouse cell contamination in the PDX samples, features associated with mice were omitted, and any mouse cells identified through the 10x Genomics cellranger-atac pipeline were subsequently excluded. Before conducting dimensionality reduction, cells meeting the following criteria were subjected to further analysis: peak region fragments between 1000 and 20000, a blacklist fraction below 0.05, at least 20% of reads within peaks, nucleosome signal below 1, and TSS enrichment above 2. Following QC steps, we implemented the Signac pipeline with uniform parameters for dimensionality reduction and gene activity calculation, maintaining consistency with those applied to our PDX samples.

Transcription Factor Footprinting
Footprints are generated by TFs bound to DNA, which inhibits the Tn5 transposase from cleaving DNA within nucleosome-free regions. The HINT-ATAC algorithm82, a Hidden Markov Model (HMM)-based approach, was utilized to discern TF binding sites associated with footprints.
In order to delineate transcription factor (TF) footprints within individual samples employing HINT-ATAC, we initially prepared indexed BAM files and peak files derived from the aggregation of cells originating from each respective sample. Peak files were generated using the MACS2 callpeak function with the following parameters: -g hs -q 0.01 --nomodel --shift -100 --extsize 200 -B --SPMR --call-summits. Subsequently, we initiated footprints identification by running the function rgt-hint footprinting specifying parameters including: --atac-seq --paired-end --organism=hg38. Following footprint calling, TF-associated footprints within each sample were discerned through the detection of motifs sourced from the JASPAR vertebrates database that overlapped with the predicted footprints, facilitated by the rgt-motifanalysis matching function. Finally, to ascertain average single-cell ATAC sequencing (scATAC-seq) profiles surrounding the binding sites of each TF across individual samples, we applied the rgt-hint differential function with the following parameters: --organism=hg38 --bc --nc 32.
To identify the differences in transcription factor (TF) activities between MYOD1 and MYOD1L122R samples, we utilized publicly available MYOD1 scATAC-seq datasets from the St. Jude Childhood Solid Tumor Network. Specifically, we generated an indexed BAM file for the MYOD1L122R samples by aggregating data from MSK93202, MSK74711, MAST161, and ST67 using samtools merge function67. Similarly, we merged all MYOD1 samples, encompassing SJRHB010927_X1, SJRHB010928_X1, SJRHB013758_X1, and SJRHB013758_X2, to generate another indexed BAM file. Subsequently, we generated corresponding peak files utilizing the MACS2 70 callpeak function. We employed the same analytical HINT-ATAC workflow and predicted TF footprints across both MYOD1 and MYOD1L122R samples.
Codes used in this study was available through E.R. Github account >rheinbaylab > Wei_MYOD_RMS repository with the link https://github.com/rheinbaylab/Wei_MYOD_RMS.

For manuscripts utilizing custom algorithms or software that are central to the research but not yet described in published literature, software must be made available to editors and reviewers. We strongly encourage code deposition in a community repository (e.g. GitHub). See the Nature Portfolio guidelines for submitting code & software for further information.

Data

Policy information about availability of data

All manuscripts must include a data availability statement. This statement should provide the following information, where applicable:

- Accession codes, unique identifiers, or web links for publicly available datasets
- A description of any restrictions on data availability
- For clinical datasets or third party data, please ensure that the statement adheres to our policy

Raw and processed data is made available under the NIH GEO accession number GSE274640.

Research involving human participants, their data, or biological material

Policy information about studies with human participants or human data. See also policy information about sex, gender (identity/presentation), and sexual orientation and race, ethnicity and racism.

Reporting on sex and gender	We don't have human participants in this study, sex or gender information is not collected or provided on PDX samples as it's not relevant to investigate the molecular function of mutant MYOD1.
Reporting on race, ethnicity, or other socially relevant groupings	Again not involving human participants.
Population characteristics	No human participants except de-identified PDX or primary patient sample slides with the knowledge of whether mutant MYOD1 is present or not.
Recruitment	No human participants involved.
Ethics oversight	Patient-derived xenograft models were obtained from Memorial Sloan Kettering Cancer Center (F.D.C), St. Jude Children's Hospital (A.G.P), and Baylor College of Medicine (Dr. Nino Rainusso), with patient consents signed and IRB protocols

approved from these institutes. Single-cell RNA-sequencing analysis used some previously published data sets (GSE195709 and GSE174376), see Supplementary Table 4 for details. Patient information was blinded to researchers in this study.

Note that full information on the approval of the study protocol must also be provided in the manuscript.

Field-specific reporting

Please select the one below that is the best fit for your research. If you are not sure, read the appropriate sections before making your selection.

☒ Life sciences ☐ Behavioural & social sciences ☐ Ecological, evolutionary & environmental sciences

For a reference copy of the document with all sections, see nature.com/documents/nr-reporting-summary-flat.pdf

Life sciences study design

All studies must disclose on these points even when the disclosure is negative.

Sample size	All experiments in cell line or zebrafish work described here were performed in at least triplicate with detailed mean/median/average and p-values described in figures and figure legends. All single-cell RNA-seq or ATAC-seq were only performed in one patient-derived sample, considering the availability of the patient samples and the cost of the experiment, with an average cell load of 5,000-10,000 cells. ChIP-seq experiments were performed in duplicates, and RNA-seq were performed in at least triplicate for individual experimental group. Quality control (QC) was performed both at the bench stage, and data preprocessing and analysis. Batch effects were removed when comparing samples from different experiments, or from publications.
Data exclusions	No assessed data was excluded from this research.
Replication	Results for these experiments described in this manuscript with replicates are all replicable and reported here. No exception or exclusions.
Randomization	Random fish were used for tumor generation, tumor receiving of transplants. Also, random field of images were taken for imaging representation, and quantification.
Blinding	Yes, most image collection of immunofluorescence staining, immunohistochemistry, or colonagenic assays are captured or analyzed by a research technician who is blinded from the hypothesis, who is a co-author in this manuscript by performing this task.

Reporting for specific materials, systems and methods

We require information from authors about some types of materials, experimental systems and methods used in many studies. Here, indicate whether each material, system or method listed is relevant to your study. If you are not sure if a list item applies to your research, read the appropriate section before selecting a response.

Materials & experimental systems

n/a	Involved in the study	
☐	☒	Antibodies
☐	☒	Eukaryotic cell lines
☒	☐	Palaeontology and archaeology
☐	☒	Animals and other organisms
☒	☐	Clinical data
☐	☒	Dual use research of concern
☒	☐	Plants

Methods

n/a	Involved in the study	
☐	☒	ChIP-seq
☐	☒	Flow cytometry
☒	☐	MRI-based neuroimaging

Antibodies

Antibodies used	All antibodies used in this study was compiled and submitted as Supplementary Table 8, including the purposes, antigens, host species, manufactures and catlog number.
Validation	All antibodies used in this study have been validated and published before through these manufacturers. No in-house new clones were generated or used in this study.

Eukaryotic cell lines

Policy information about cell lines and Sex and Gender in Research

Cell line source(s)	All cell lines used here are from ATCC, or from previously published research. Yet cell lines have been further validated by ATCC STR Profiling kit to match with original patient short tandem repeat information.
Authentication	Cell lines have been authenticated by ATCC STR Profiling kit to match with original patient short tandem repeat information.

Mycoplasma contamination	No mycoplasma contamination was detected in these cells used. All cells used in this study are regularly checked on a monthly basis for potential mycoplasma contamination.
Commonly misidentified lines (See ICLAC register)	No misidentified lines to be reported.

Animals and other research organisms

Policy information about studies involving animals; ARRIVE guidelines recommended for reporting animal research, and Sex and Gender in Research

Laboratory animals	6-week-old female NSG (NO SCID gamma- Prkdc scid il2rg tm1Wjl) mice were used for PDX passage used in this study. TuAB wildtype or syngenic CG1 zebrafish with the age between 0 day - 12 months were used in this study.
Wild animals	No wild animals were used.
Reporting on sex	Sex was not considered in this study. Random sex was used for zebrafish studies. Female NSG mice were chosen because of ease of housing them in one cage. In addition, sex does not affect our research of mutant MYOD1.
Field-collected samples	No samples were collected from the field.
Ethics oversight	No ethics oversight as no human subjects were involved. No wild animals were collected from the wild or field.

Note that full information on the approval of the study protocol must also be provided in the manuscript.

Dual use research of concern

Policy information about dual use research of concern

Hazards

Could the accidental, deliberate or reckless misuse of agents or technologies generated in the work, or the application of information presented in the manuscript, pose a threat to:

No	Yes	
☒	☐	Public health
☒	☐	National security
☒	☐	Crops and/or livestock
☒	☐	Ecosystems
☒	☐	Any other significant area

Experiments of concern

Does the work involve any of these experiments of concern:

No	Yes	
☒	☐	Demonstrate how to render a vaccine ineffective
☒	☐	Confer resistance to therapeutically useful antibiotics or antiviral agents
☒	☐	Enhance the virulence of a pathogen or render a nonpathogen virulent
☒	☐	Increase transmissibility of a pathogen
☒	☐	Alter the host range of a pathogen
☒	☐	Enable evasion of diagnostic/detection modalities
☒	☐	Enable the weaponization of a biological agent or toxin
☒	☐	Any other potentially harmful combination of experiments and agents

Plants

Seed stocks

No seed or plants involved.

Novel plant genotypes

No seed or plants involved.

Authentication

No seed or plants involved

ChIP-seq

Data deposition

- [x] Confirm that both raw and final processed data have been deposited in a public database such as GEO.
- [x] Confirm that you have deposited or provided access to graph files (e.g. BED files) for the called peaks.

Data access links
May remain private before publication.

Raw and processed data is made available under the NIH GEO accession number GSE274640.

Files in database submission

fastq file, Bam file, bed files

Genome browser session
(e.g. UCSC)

Initial submission, Ensemble, Human Genome GRCh38.

Methodology

Replicates

ChIP-seq experiments were performed in duplicates for individual experimental group.

Sequencing depth

NextSeq 2000, along with P3 reagents (100 cycles) was used for the sequencing run (Illumina # 20040559). On average, >20M reads per sample were acquired, covering 75bp from paired ends for ChIP-seq libraries.

Antibodies

Antibodies used in this study was provided in Supplementary Table 7.

Peak calling parameters

The determination of the ChIP-seq peaks for each of the histone marks was carried out with the macs2 (v. 2.0.10) program70 using the "callpeak" option and input samples as a control. Difficult to map regions were obtained from ENCODE (version GRCh38)71 and removed using the "subtract" command of bedtools (v. 2.30.0)72. The differential peaks between MYOD1L122R and MYOD1 for the RD and Ruch2 cell lines FLAG ChIP-seq were obtained from the consensus between two analytical approaches. The first of these approaches was carried out using macs2. First, the average of the fragment sizes was obtained using the "predictd" option on the different samples to be contrasted. Next, the pooling of the samples of the same condition (MYOD1L122R or MYOD1) was carried out with the "callpeak" option, using the average fragment size obtained in the previous step and the input samples as a control. As a result, we obtained the effective sequencing depth for each of the conditions. Finally, the "bdgdiff" option of macs2 was used, specifying both the effective sequencing depth of each group ("--d2" option), the minimum distance to be considered between two significant peaks (60 bases in the case of the FLAG epitope, "-g" option) and the minimum length of the significant peaks (120 bases for FLAG, "-l" option). Peaks that obtained a q-value lower than 0.05 were considered significant. In the second approximation, differential peaks were called using the R package DiffBind (v. 3.8.4)73 together with edgeR (v. 3.40.2)74. The MACS2-TMM method was used as a method of normalization of the peaks and the determination of the differential peaks between the different conditions was carried out by the adjustment of quasi-likelihood negative binomial generalized log-linear model. Peaks with a Benjamini-Hochberg-adjusted p-value lower than 0.05 were considered significant. To determine the consensus peaks between the two detection methods, we calculated the intersection of the peaks obtained from the two approaches using the bedtools tool in "intersect" mode. All selected peaks were annotated using the "annotatePeaks" function of HOMER (v.4.10)31. This approach allowed us to establish three groups of peaks for the FLAG epitope in the two cell lines: the exclusive peaks of MYOD1L122R (selected in the macs2 approach as exclusive of "condition1", and significantly enriched in the DiffBind approach [adj.p < 0.05 and Fold > 0]) the exclusive peaks of MYOD1 (selected in the macs2 approach as exclusive of "condition2", and significantly enriched in the DiffBind approach [adj.p < 0.05 and Fold < 0]) and the peaks shared between the two conditions (selected in the macs2 approach as "common" to the two conditions and not significant in the DiffBind approach [adj.p > 0.05]).

Data quality

This approach allowed us to establish three groups of peaks for the FLAG epitope in the two cell lines: the exclusive peaks of MYOD1L122R (selected in the macs2 approach as exclusive of "condition1", and significantly enriched in the DiffBind approach [adj.p < 0.05 and Fold > 0]) the exclusive peaks of MYOD1 (selected in the macs2 approach as exclusive of "condition2", and significantly enriched in the DiffBind approach [adj.p < 0.05 and Fold < 0]) and the peaks shared between the two conditions (selected in the macs2 approach as "common" to the two conditions and not significant in the DiffBind approach [adj.p > 0.05]).

Software

MACS2-TMM was used for ChIP analysis.

Flow Cytometry

Plots

Confirm that:

- [x] The axis labels state the marker and fluorochrome used (e.g. CD4-FITC).
- [x] The axis scales are clearly visible. Include numbers along axes only for bottom left plot of group (a 'group' is an analysis of identical markers).
- [x] All plots are contour plots with outliers or pseudocolor plots.
- [x] A numerical value for number of cells or percentage (with statistics) is provided.

Methodology

Sample preparation	Cells were collected fresh for the flow analysis. Antibodies included PE-CD90 (BioLegend #328109) and FITC-CD44 (BioLegend #338803) used at the dilution of 1:200 in flow buffer (PBS, with 1% FBS and 1% NaN3). Aldefluor kit from StemCell technologies (Catalog# 01700) was used to detect ALDH activity in freshly collected cells. DEAB reagent was used to inhibit ALDH activity from the same kit to control for background fluorescence. DAPI was used to counter-select dead cells.
Instrument	The SORP 5-Laser BD LSRFortessa X-20 was used to perform flow analysis
Software	FlowJo (v.10.10.0) was used for data analysis and presentation.
Cell population abundance	Purity over 90% post sorting was validated post sorting for zebrafish tumor cell transplants.
Gating strategy	Supplementary Figure 1 showed gating strategy for fish cell sorting. Similar strategy was used for human cell flow analysis. Basically, SSC-H over SSC-A to remove doublets, FSC-A over SSC-A to exclude non-cell particles/debris, DAPI-negative for viable cells, and FITC, PE, APC were fluorophores chosen for staining of antibodies.

- [x] Tick this box to confirm that a figure exemplifying the gating strategy is provided in the Supplementary Information.
