# Supplementary material for: MYOD1 mutation drives cancer stem cell pathways and therapy-resistance in spindle cell/sclerosing rhabdomyosarcoma
Source: Nat Commun. 2026 Jun 3;17:7129. doi: 10.1038/s41467-026-73546-7 (PMC13396186; doi:10.1038/s41467-026-73546-7)
Supplement: Supplementary file 3 — Description of Additional Supplementary Files [file 41467_2026_73546_MOESM3_ESM.pdf]

Title: Supplementary Data 1

Description: Genes that are differentially regulated (DEGs) by MYOD1L122R based on bulk RNA-sequencing of engineered cell models, log2 Fold Change (log2FC) for magnitude (positive = up, negative = down) and adjusted p-values (padj) for significance, cutoffs, log2FC > 1 and padj < 0.05 to identify Differentially Expressed Genes (DEGs), statistical method used is DESeq2.

Title: Supplementary Data 2

Description: PDX and patient samples used in our work.

Title: Supplementary Data 3

Description: ChIP-seq binding sites of flag-tagged wildtype or mutant MYOD1 in RD cells.

Title: Supplementary Data 4

Description: ChIP-seq binding sites of flag-tagged wildtype or mutant MYOD1 in Ruch2 cells.

Title: Supplementary Data 5

Description: Progenitor genes that are specifically bound by mutant MYOD1L122R.

Title: Supplementary Data 6

Description: STR profile and identified mutations for patient-derived spindle cell/sclerosing RMS cell line JH-SRMS-7a.

Title: Supplementary Data 7

Description: Antibodies used for Western blot, immunofluorescence, flow analysis and/or ChIP-seq.
