# Supplementary material for: MYOD1 mutation drives cancer stem cell pathways and therapy-resistance in spindle cell/sclerosing rhabdomyosarcoma
Source: Nat Commun. 2026 Jun 3;17:7129. doi: 10.1038/s41467-026-73546-7 (PMC13396186; doi:10.1038/s41467-026-73546-7)
Supplement: Supplementary file 5 — Transparent Peer Review file [file 41467_2026_73546_MOESM5_ESM.pdf]

nature portfolio

Peer Review File

MYOD1 Mutation drives Cancer Stem Cell pathways and Therapy-resistance in Spindle cell/Sclerosing Rhabdomyosarcoma

Corresponding Author: Dr David Langenau

This file contains all reviewer reports in order by version, followed by all author rebuttals in order by version.

Version 0:

Reviewer comments:

Reviewer #1

(Remarks to the Author)
Summary: In the paper "MYOD1 Mutation drives Cancer Stem Cell pathways and Therapy-resistance in Spindle cell/Sclerosing Rhabdomyosarcoma", Langenau and colleagues investigate the role MYOD1L122R plays in the oncogenesis of spindle cell/sclerosing rhabdomyosarcoma (SS-RMS). The authors leverage established zebrafish models of rhabdomyosarcoma to demonstrate that expression of MYOD1L122R is insufficient to induce tumor formation on its own, and requires the presence of a constitutively active RAS. However, the tumors that form with the expression of MYOD1L122R develop faster, are more aggressive, and contain a higher proportion of tumor propagating cells (TPCs) than WT MYOD1 RMS tumors. The authors also demonstrate that the tumors that form in the MYOD1L122R expressing cells are spindle-like in that they have a higher cytoplasmic to nuclear ratio than WT MYOD1 expressing RMS tumors. The authors then shift to human models of disease where they use genetically engineered RD and Ruch2 human RMS cell lines to investigate how addition of MYOD1L122R changes the properties of these cells. Using WT or MYOD1L122R over-expression models, and a CRIPSR/Cas9 knock-in model, the authors show that MYOD1L122R changes RMS cells to be more stem-like and resistant to chemotherapy and radiation, recapitulating clinical observations of SS-RMS. Comparing RNAseq data from the engineered cell lines and patient derived xenografts, the authors show that transcriptionally MYOD1L122R mutant RMS is more progenitor-like, and that one of the genes uniquely expressed in MYOD1L122R cells is ROR2. The authors show that this transcriptional rewiring occurs because MYOD1L122R binds to MYC/MAX motifs, as opposed to the canonical MYOD1/MYOG motif, and that ROR2 is one of the newly acquired targets of MYOD1L122R that is upregulated in SS-RMS. Finally, the authors demonstrate the clinical importance of this molecular work by leveraging an antibody-drug conjugate targeting ROR2 to selectively kill MYOD1L122R mutant RMS. The authors of this work did an excellent job molecularly dissecting the importance of the MYOD1L122R mutation in a number of RMS models, and extend these findings clinically to a target for an antibody-drug conjugate. Overall, the work in this manuscript is rigorous, and supports the stated conclusions. A few outstanding questions remain that could strengthen the manuscript even more:

Figure 2a-b: Overexpression of the WT MYOD1 appears to be far less significant that the mutant MYOD1 in the Ruch2 cell lines (and the opposite seems to be true in the RD cell line). Can unequal levels of expression factor in to observed phenotypes? The authors may want to discuss this issue- but given that phenotypes are the same in both cell lines with the mutant, the argument is still valid as to the gain of function effects of the MYOD1 mutant.

Figure 2g: In the Ruch2 OE tumorspheres, MYOD1 WT appears to significantly influence the structure of these tumorspheres. Is it affecting cell viability, and are these cells reliant on Myf5 normally and overexpression of MYOD1 causes some sort of defect? The authors may want to discuss this striking change in tumorsphere behavior with OE of WT MYOD1.
Figure 3b: The authors show a right shift in dose responses after VAC and IVA treatment with overexpression of the MYOD1L122R mutant. Is this shift significant in all settings?

Figure 5a: Of all of the peaks that are L122R exclusive, what percentage of the genes annotated to those peaks are genes driving the progenitor score in Figure 4?

Figure 5a: Is MYOG one of the genes in the MYOD1 exclusively regulated set, or does it expression decrease in western blots because you are losing the more differentiated population of RMS cells with MYOD1L122R?

Figure 5c: Since the L122R has a much higher preference for MYC binding sites, could this in part be due to increased MYC levels in these mutants (and subsequent cooperative binding)? Has that been examined?

Figure 6: Quantitation of the IHC data for ROR2 should be shown in MYOD samples vs L122R and statistics should be performed to demonstrate the clear correlation of ROR2 protein with the MYODL122R mutant.

Figure 6: ROR2 expression correlates well with the L122R mutant in scRNA-seq data from PDX models. Can a similar analysis be done from direct patient samples (even in bulk?).

(Remarks on code availability)
N/A

Reviewer #2

(Remarks to the Author)
Summary
Wei and Sanchez et al present a comprehensive study understanding the role of MYOD1 mutation in influencing cancer stem cell properties in spindle-cell/sclerosing rhabdomyosarcoma (SS-RMS). This disease is one of the most aggressive forms of rhabdomyosarcoma, with poor overall survival and no targeted therapies. They develop multiple models to understand how the mutant MYOD functions, including expression in transgenic zebrafish systems, overexpressing and introducing the point mutation in human embryonal rhabdomyosarcoma cell lines. They found that mutation L122R within the DNA-binding motif of MYOD1 upregulates tumor-propagating cells and expression of stem-cell markers and prevents cellular differentiation. This mutation also leads to gain of resistance to therapy techniques of chemotherapy and radiation. At the transcriptomics level, they showed that MYOD1 L122R mutation elevates cancer stemness gene signatures and suppresses muscle differentiation signatures. In addition, the authors found a direct target of MYOD1 L122R mutation, ROR2, which was shown to activate the WNT/PCP signaling pathway contributing to persistence cancer stem cell-like characteristics and resistance to therapeutic measures. The studies described in this manuscript are very comprehensive and the progression between sections is clearly described. The manuscript is well written. Areas that could be improved on are a more thorough evaluation of the zebrafish tumor model, mechanistic insights into how mutant MYOD adopts a MYC profile, and elaborating on ROR2 as a therapeutic target in vivo. Overall, this study would be of broad interest to the rhabdomyosarcoma, cancer research, and potentially skeletal muscle community. The specific comments are below:

Specific Comments
1. Mutant MyoD adopting a MYC-like profile. How is MYC, or is MYC involved in the mechanism of mutant MYOD transformation. Does ROR2 have the MAX::MYC CACGTG motif? Does mutant MYOD now colocalize with MYC in the genome? Does mutant MYOD aberrantly dimerize with MYC for transcriptional targeting?

2. Characterization of the MYODL122R zebrafish model. Were these tumors evaluated outside of pathology? Could the pathologist elaborate on how KRAS tumors have differing histology than KRAS+MYODL122R tumors. Is the transcriptional signature of KRAS+MYODL122R tumors comparable to the human disease? The authors should also elaborate on the rationale for choosing KRAS as the cooperating gene instead of PTEN or PI3KCA.

3. ROR2 as a therapeutic target. Validating this as a therapeutic target in vivo would strengthen the conclusions. For example, generating a ROR2 knockdown/knockout in MYOD1L22R mutant embryonal rhabdomyosarcoma cell lines and performing xenograft experiments to see if it suppresses tumor growth.

4. Discussion of MYODL122R's role in the disease if it is not an oncogene. It's striking the MYODL122R mutation is not transforming alone, but really defines one of the most aggressive subtypes of rhabdomyosarcoma. Additional context on the author's thoughts of how MYODL122R its functioning to support this aggressive disease, and what is most important for transformation, would help interpretation of its role in the disease.

Minor Comments
1. In the text the MYOD1 mutant form of ssRMS is referred to generally as ssRMS, which broadly encompasses a few different rhabdomyosarcoma subtypes, and the conclusions suggest its applicable to the entire group. For example, in line 453, this is a therapeutic opportunity for MYOD1 mutant SSRMS, not the entire histological subgroup.

2. Line 133 states that zebrafish with co-expression of MYOD1 L122R and KRAS G12D mutations had an early tumor onset compared to MYOD1 wildtype and KRAS G12D. In Figure 1B, the onset looks similar between the 3 condition, this statement could be removed.

3. Figure 1, Extended Data Figure 1b: It will be beneficial to mention the Tu/AB and CGI zebrafish strain on top of the respective figure panels. Also, mention fish age in panel D of Figure 1 or in Methods.

4. Results section 2, paragraph 2, Extended Data Figure 4: The authors mention that MYOD1 L122R RMS cells failed to differentiate in low serum conditions. It will help in appreciating the cell morphology and compare differentiated cells to the MYOD1 wildtype group if the authors include phase contrast microscopy images.

5. There’s a comment in Extended Data Figure S3 that should be deleted.

6. Line 210-213: ‘Upregulated genes were enriched for epithelial-to-mesenchymal transition (EMT) genes that have previously been shown to be enriched in FN cancer stem cells, while downregulated genes contained myogenesis and muscle differentiation genes’. This needs a citation.

7. Growth assays or description of cell titer glo is missing in the methods section.

8. Github link may still be set to private: https://github.com/rheinbaylab/Wei_MYOD_RMS

(Remarks on code availability)
I was unable to review the code because the link did not work. I think it may still be set to private.

Reviewer #3

(Remarks to the Author)
This is a well written and comprehensive study of the neomorphic transcriptional activity of mutant MYOD1 in rhabdomyosarcoma. While rare this is a clinically significant mutation that is associated with aggressive disease. I believe this manuscript will be a critical data set understanding the mechanism of the mutation. In addition the authors describe a potential therapeutic strategy targeting a downstream cell surface target of the mutation that has immediate clinical significance.
Some thoughts to improve the presentation and the manuscript.
1. Has the author tried PIK3CA co-mutation in the zebrafish. While there is some redundancy with RAS pathways these are not directly overlapping.
2. Can the authors comment on the penetrance of the co-mutation. Is there a reason why <50% get tumors but when sequentially passed they are 100% penetrant?
3. Fig2a. The Ruch2 construct seems leaky with significant HA expression in the non-induced state. Given the potency of the mutation would the authors not anticipate that this would be problematic in interpretation of downstream genes?
4. In the RD cell line what happens to the endogenous WT MYOD1 expression when the mutation is present? Does the mutant feedback and diminish the endogenous expression?
5. 4a and b. The “genes” label is a little confusing. Why do they just select 17 genes to present here? I assume they think these are the critical ones but how were they selected out of the bigger group? Are these from the leading edge genes of the GSEA? The Z-score of the heatmap is not super informative. It would be nice to get a sense of the actual expression of these genes rather than a comparison between mutant and non-mutant.
6. Fig 4d. Its hard to tell in the single cell data from the plots but it appears that each of the MYOD1 mutant tumors might have at least a couple of distinct cluster while the WT tumors are more of a cloud. Are these transcriptional sublones or groups of cells dividing?
7. In Fig5a are the RD cells with FLAG constructs single cell cloned or is more heterogeneous construct. The methodology of the cell line construction in this paper should be robust and detailed as these reagents and the data coming from may be important for future investigations in the field.
8. Figure 5a. Can the authors include Chipseq using a MYOD1 antibody in the parental cell line? It is possible that some of the L122R “exclusive sites” have endogenous WT MYOD1 at these genomic locations. In parallel it would be nice to show chipseq of MYC in the same manner.
9. Fig 5e and 5f. It would be helpful to add RNAseq tracks of these genes and loci.
10. It’s a little surprising that the PDX trial of the ROR2 targeting ADC are not included in this work.

(Remarks on code availability)
I was not able to access through the github link provided. Perhaps needs to be made public.

Version 1:

Reviewer comments:

Reviewer #1

(Remarks to the Author)
The authors have done an excellent job addressing all previous concerns.

A very minor comment- in extended data Fig. 13a-c, they should clearly mark on the figure which tumors are WT vs MUT.

(Remarks on code availability)
I cannot review the code well- as I am not an informatician

Reviewer #2

(Remarks to the Author)
The authors have thoroughly addressed my concerns and significantly strengthened the manuscript.

(Remarks on code availability)

Reviewer #3

(Remarks to the Author)
The authors responses appropriately addressed my comments.

(Remarks on code availability)

Open Access This Peer Review File is licensed under a Creative Commons Attribution 4.0 International License, which permits use, sharing, adaptation, distribution and reproduction in any medium or format, as long as you give appropriate credit to the original author(s) and the source, provide a link to the Creative Commons license, and indicate if changes were made.
In cases where reviewers are anonymous, credit should be given to 'Anonymous Referee' and the source.
The images or other third party material in this Peer Review File are included in the article's Creative Commons license, unless indicated otherwise in a credit line to the material. If material is not included in the article's Creative Commons license and your intended use is not permitted by statutory regulation or exceeds the permitted use, you will need to obtain permission directly from the copyright holder.
To view a copy of this license, visit https://creativecommons.org/licenses/by/4.0/

REVIEWER COMMENTS –

Reviewer #1:

Summary of work and impact: *"The authors of this work did an excellent job molecularly dissecting the importance of the MYOD1L122R mutation in a number of RMS models, and extend these findings clinically to a target for an antibody-drug conjugate. Overall, the work in this manuscript is rigorous, and supports the stated conclusions. A few outstanding questions remain that could strengthen the manuscript even more."*

1) Figure 2a-b: Overexpression of the WT MYOD1 appears to be far less significant that the mutant MYOD1 in the Ruch2 cell lines (and the opposite seems to be true in the RD cell line). Can unequal levels of expression factor in to observed phenotypes? The authors may want to discuss this issue- but given that phenotypes are the same in both cell lines with the mutant, the argument is still valid as to the gain of function effects of the MYOD1 mutant.

As noted by the reviewer, the gain of function effects of mutant MYOD1 were the same across our cell line models and replicated many times. Yet, the original Westerns images errantly showed differences in the level of induced MYOD1 protein expression between RUCH2 and RD. We have replaced Figure 2a with a more representative example of our Western results.

2) Figure 2g: In the Ruch2 OE tumorspheres, MYOD1 WT appears to significantly influence the structure of these tumorspheres.

While there is variability between different spheres formed in the assay, we do not see differences in the overall size or morphology of spheres between RUCH2 cells overexpressing WT or mutated MYOD1. We have replaced the images in figure 2G with a larger field of view to better highlight that the frequency of sphere formation is elevated following expression of $MYOD^{1L122R}$, which is the major finding of this work.

3) Figure 3b: The authors show a right shift in dose responses after VAC and IVA treatment with overexpression of the MYOD1L122R mutant. Is this shift significant in all settings?

The right shift in dose responses is indeed significant. We have added statistical analysis to the IC^{50} plots shown in Figure 3b. We apologize for not including this in the first version of the manuscript.

4) Figure 5a: Of all of the peaks that are L122R exclusive, what percentage of the genes annotated to those peaks are genes driving the progenitor score in Figure 4?

Of 14,339 peaks exclusive to $MYOD1^{L122R}$, 4,201 were associated with protein-coding genes in the RD model. Among these, 141 peaks were linked to 73 unique genes from the progenitor signature. In total, 42.7% of the genes associated with the progenitor signature contained $MYOD1^{L122R}$ bound peaks (n=73 of the 171 signature genes). New Supplementary Table 7 now describes this list and a statement regarding this finding in now included in the revised manuscript.

5) Figure 5a: Is MYOG one of the genes in the MYOD1 exclusively regulated set, or does it expression decrease in western blots because you are losing the more differentiated population of RMS cells with MYOD1L122R?

Mutant MYOD1 ^{L122R} shows extremely little enrichment at the *Myogenin (MYOG)* promoter compared to wild-type MYOD. This suggests that the downregulation of Myogenin is at least in part due to differential binding of mutated MYOD1 ^{L122R}. Below is a reviewer figure demonstrating MYOD1 ^{L122R} and MYOD1 binding to the *MYOG* promoter ("FLAG" in the upper and lower panel) after dox-induction (2 µg/mL) for 72 hours. RNA-seq tracks shown are from each respective model.

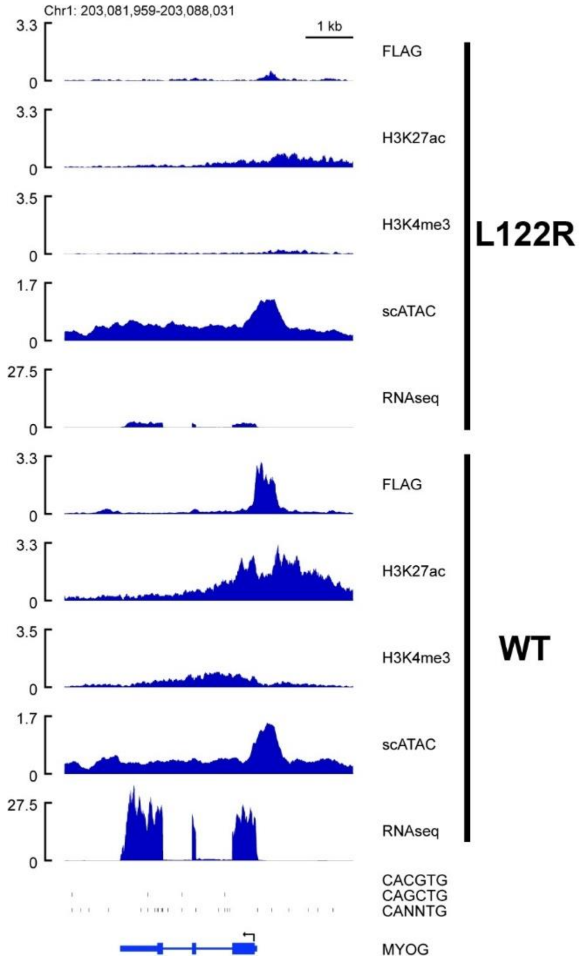

6) Figure 5c: Since the L122R has a much higher preference for MYC binding sites, could this in part be due to increased MYC levels in these mutants (and subsequent cooperative binding)? Has that been examined?

We thank the reviewer for this important question. We addressed this point through several experiments: (1) RNA sequencing and Western Blot analysis show that MYC mRNA and protein expression are not differentially regulated in our MYOD1 ^{L122R} models. (2) We tested whether MYC directly binds to MYOD1 or MYOD1 ^{L122R} using a proximity ligation assay (PLA) – a technique that enables highly specific in situ detection of protein–protein interactions or conformational changes. Pairs of antibodies conjugated to oligonucleotides undergo ligation when in close proximity (<40 nm). Subsequent rolling circle amplification generates a localized

fluorescent signal that can be quantitatively imaged. PLA confirmed MYOD1:MYOD1 homodimerization and cooperative binding of MYOD1:MYOD1^{L122R}; yet dimerization between MYC and either MYOD1 or MYOD1^{L122R} was not observed. Control experiments confirmed MYC:MAX interactions while single-antibody experiments showed no rolling circle amplification.

(3) We further performed co-immunoprecipitation of HA-MYOD1 or HA-MYOD1^{L122R} along with FLAG-MYC in HEK293T cells followed by Western blot for antibodies for anti-FLAG or anti-HA (HA-MYOD or FLAG-MYC). In these experiments, both MYOD1 or MYOD1^{L122R} failed to bind to MYC. In contrast, co-immunoprecipitation of either HA-MYOD1 or HA-MYOD1^{L122R} and FLAG-MYOD confirmed MYOD1:MYOD1 homodimerization and cooperative binding of MYOD1:MYOD1^{L122R}. Together, these results show that MYC does not directly associate with MYOD1^{L122R}, suggesting that the regulatory function of MYOD1^{L122R} is independent of MYC. We have collated these experiments into Extended Data Figure 12 and have now included additional results and discussion related to these new findings.

7) Figure 6: Quantitation of the IHC data for ROR2 should be shown in MYOD samples vs L122R and statistics should be performed to demonstrate the clear correlation of ROR2 protein with the MYODL122R mutant.

Following the reviewers request, we have now quantified IHC results by patient. In addition, we have validated high ROR2 expression in MYOD1 ^{L122R} mutated SS-RMS in additional tumors. These include seven additional MYOD-wildtype RMS and six MYOD1^{L122R} mutated RMS from St. Jude Research Hospital. Drs. Anand Patel and Selene Koo independently performed anti-ROR2 staining and quantification at their institution (St. Jude Research Hospital), which has now been added to our previous sample analysis (Extended Figure 14). Together, our work details the expression of ROR2 across 13 patients by IHC, 4 PDX models by ss-RNA sequencing, and three primary patient samples by bulk-RNA sequencing, confirming elevated ROR2 expression in all analyzed MYOD1 ^{L122R} mutated tumors analyzed. By contrast, most FN-RMS do not express high levels of ROR2.

8) Figure 6: ROR2 expression correlates well with the L122R mutant in scRNA-seq data from PDX models. Can a similar analysis be done from direct patient samples (even in bulk?).

We now provide analysis of patient RNA sequencing data from the DeMartino primary RMS and BeatCC pediatric RMS tumor datasets to address this point. As expected, we see elevated ROR2 transcript expression in 3 of 3 mutant MYOD1 ^{L122R} patient when compared with FN-RMS samples that have reduced levels of ROR2 expression (presented in new Extended Data Figure 13b-c). As noted above in reviewer point 7, we have also now performed additional IHC staining on patient samples, confirming that 13 of 13 MYOD1^{L122R} mutated SS-RMS have high ROR2 expression, and its expression is found in a majority of tumor cells.

Reviewer #2:

Summary of work and impact: *"The studies described in this manuscript are very comprehensive and the progression between sections is clearly described. The manuscript is well written. Areas that could be improved on are a more thorough evaluation of the zebrafish tumor model, mechanistic insights into how mutant MYOD adopts a MYC profile, and elaborating on ROR2 as a therapeutic target in vivo. Overall, this study would be of broad interest to the rhabdomyosarcoma, cancer research, and potentially skeletal muscle community."*

1a) Mutant MyoD adopting a MYC-like profile. How is MYC, or is MYC involved in the mechanism of mutant MYOD transformation. Does mutant MYOD now colocalize with MYC in the genome? Does mutant MYOD aberrantly dimerize with MYC for transcriptional targeting?

We thank the reviewer for these important questions. In several relevant experiments, we found no cooperative binding of MYOD1^{L122R} with MYC. This is now supported by a wide array of new analysis and experiments completed since our initial submission as detailed below.

i) We do not observe a difference in the expression of MYC mRNA or protein in the MYOD^{L122R} models. Specifically, RNA sequencing and Western Blot analysis confirmed that MYC transcription and protein expression are not differentially regulated in our MYOD1^{L122R} models (now presented in a new Extended Data Figure 12).

ii) We assessed potential association of MYC and MYOD1/ MYOD1^{L122R} using proximity ligation assay (PLA), a technique that enables highly specific in situ detection of protein–protein interactions or conformational changes by using pairs of antibodies conjugated to oligonucleotides that, when in close proximity (<40 nm), undergo ligation and rolling circle amplification, generating a localized fluorescent signal that can be quantitatively imaged. PLA confirmed MYOD1:MYOD1 homodimerization and cooperative binding of MYOD1:MYOD1^{L122R}; yet dimerization between either MYOD1 or MYOD1^{L122R} with MYC was not observed. Control experiments confirmed MYC:MAX interactions while single antibody experiments showed no rolling circle amplification. These results suggest that MYC does not associate with MYOD1 or Mutant MYOD1^{L122R}.

(iii) We performed co-immunoprecipitation using HA-conjugated beads in HEK293T cells that expressed either HA-MYOD1 or HA-MYOD1^{L122R} and FLAG-MYC followed by Western blot. In this experiment, we did not observe binding of MYOD1 or MYOD1^{L122R} to MYC. As expected, our controls confirmed MYOD1:MYOD1 homodimerization and cooperative binding of MYOD1:MYOD1^{L122R}.

Together, these results show that MYC does not directly associate with MYOD1^{L122R}, suggesting that the regulatory function of MYOD1^{L122R} is independent of MYC. We have collated these experiments into Extended Data Figure 12 and have now included additional results and discussion related to these new findings.

1b) Does ROR2 have the MAX::MYC CACGTG motif?

We apologize for not better denoting this in the original manuscript. The ROR2 gene locus does indeed have several CACGTG MAX:MYC motifs but largely lacks the MYOD binding sites – now better shown with enlarged text in Figure 6b.

2a) Characterization of the MYODL122R zebrafish model. Were these tumors evaluated outside of pathology? Could the pathologist elaborate on how KRAS tumors have differing histology than KRAS+MYODL122R tumors.

All zebrafish tumors were analyzed by board-certified pathologist Dr. Peter Neilsen – a world-recognized sarcoma pathologist with expertise in human and animals models of rhabdomyosarcoma. To date he has performed pathology analysis with our group on many publications (Wei et al. Nature Cancer, 2022; Ignatius et al., Elife 2018; Hayes et al., Cell Stem Cell 2018; Ignatius et al., Cell Reports 2017; Chen et al., PLoS Genetics 2013; Storer et al., Development 2013; and Ignatius et al., Cancer Cell 2012). His expert histopathological analysis found that MYOD1^{L122R}-expressing tumors had larger numbers of spindled cells and contained cells with a higher cytoplasm-to-nuclei ratio (n=19 of 23 in MYOD1^{L122R} tumors had spindle morphology compared with n=4 of 15 in kRASG12D-alone expressing tumors, $p<0.001$ Fisher's Exact test, Figure 1a, c). In addition, we observed higher numbers of proliferative cells in MYOD1^{L122R} expressing tumors as determined by phosphorylated Histone 3 (pHH3) staining with no overall changes in apoptosis when assessed by activated cleaved caspase 3 staining (CC3, Figure 1a).

2b) Authors should also elaborate on the rationale for choosing KRAS as the cooperating gene instead of PTEN or PI3KCA.

MYOD1^{L122R} mutations co-occur at a frequency of ~70% with PIK3CA/RAS pathway activating mutations. RAS is commonly mutated in this setting, comprising 5 of 17 reported cases (Shern et al., JCO 2021). We have added additional clarifying text to the manuscript to support our approach in testing cooperativity of activating mutations in RAS along with MYOD1^{L122R}.

2c) Is the transcriptional signature of KRAS+MYODL122R tumors comparable to the human disease?

We did not analyze the transcriptional profiles of zebrafish tumors or perform direct cross-species comparisons with human rhabdomyosarcoma, beyond the histopathology showing elevated spindle morphology akin to human SS-RMS, IHC, and apoptosis stains. Instead, our zebrafish studies focused on assessing the tumorigenic potential of MYOD1^{L122R} alone, its cooperation with RAS pathway activation, and the resulting functional effects on stem cell populations and tumor-propagating cell frequency through limiting dilution transplantation assays. Having observed striking findings in the zebrafish model—particularly an expansion of cancer stem cells and increased tumor-propagating capacity—we subsequently transitioned to human RMS models, where the underlying biology and cellular functions are directly relevant to the human disease context.

3) ROR2 as a therapeutic target. Validating this as a therapeutic target in vivo would strengthen the conclusions. For example, generating a ROR2 knockdown/knockout in MYOD1L22R mutant embryonal rhabdomyosarcoma cell lines and performing xenograft experiments to see if it suppresses tumor growth.

We agree with the reviewer that validation of ROR2 as a therapeutic target is important. We have added in vivo experiments in which we xenografted our RD dox-inducible models expressing either wild-type MYOD1 or MYOD1^{L122R} into NSG mice, allowed the tumors to engraft until palpable and then treated the mice with an ADC targeting ROR2 (Ozuriftamab vedotin) weekly for four weeks. As expected, WT MYOD1 tumors were not affected by the drug treatment, reflecting lack of ROR2 target expression. By contrast, MYOD1^{L122R} -expressing

tumors regressed after treatment and did not grow back over the course of our studies. This work mirrors our *in vitro* findings presented in the first submission and provides exciting new directions for clinical application. Relevant data is now included in Figure 7h-j and Extended Figure 19f-h.

4) Discussion of MYODL122R's role in the disease if it is not an oncogene. It's striking the MYODL122R mutation is not transforming alone, but really defines one of the most aggressive subtypes of rhabdomyosarcoma. Additional context on the author's thoughts of how MYODL122R its functioning to support this aggressive disease, and what is most important for transformation, would help interpretation of its role in the disease.

We have augmented the discussion to better define how mutant MYOD1 contributes to disease aggression as suggested by the reviewer. Briefly, our findings are mirrored by what is found in the clinic where MYOD1 mutations commonly co-occur with other oncogenic driver mutations including those in the PI3K/RAS pathway. Consistent with these clinical observations, $MYOD1^{L122R}$ by itself does not initiate tumors in zebrafish transgenic models (0/243 screened fish). Yet, zebrafish that expressed mutant $MYOD1^{L122R}$ had higher disease penetrance, worse survival and elevated the overall frequency of tumor propagating cells, in comparison to $KRAS^{G12D}$-alone.

Ultimately, in human cell line models, this translates to a greater capacity to (1) resist therapy and (2) regrow tumors post-therapy. However, tumor proliferation was not altered by mutant $MYOD1^{L122R}$. Thus mutant MYOD1 ^{L122R} contributes to RMS by elevating its overall aggression through resisting therapy and elevating the overall fraction of tumor propagating progenitor cells.

<u>Additional Minor Comments</u>

5) In the text the MYOD1 mutant form of ssRMS is referred to generally as ssRMS, which broadly encompasses a few different rhabdomyosarcoma subtypes, and the conclusions suggest its applicable to the entire group. For example, in line 453, this is a therapeutic opportunity for MYOD1 mutant SSRMS, not the entire histological subgroup.

We have revised the text to indicate that our studies are relevant only to the MYOD1 ^{L122R} mutated ssRMS.

6*) Line 133 states that zebrafish with co-expression of MYOD1 L122R and KRAS G12D mutations had an early tumor onset compared to MYOD1 wildtype and KRAS G12D. In Figure 1B, the onset looks similar between the 3 condition, this statement could be removed.*

Animals that co-expressed mutant $MYOD1^{L122R}$ and $KRAS^{G12D}$ had higher disease penetrance and worse survival when compared to $KRAS^{G12D}$-alone or $KRAS^{G12D}$+*MYOD1* expressing tumors. We have revised the text to state that tumor-free survival was different in fish with tumors that co-express $MYOD1^{L122R}$ and $KRAS^{G12D}$. Thank you for alerting us to this error.

7) Figure 1, Extended Data Figure 1b: It will be beneficial to mention the Tu/AB and CGI zebrafish strain on top of the respective figure panels. Also, mention fish age in panel D of Figure 1 or in Methods.

As requested, we have revised the figures to include the strains for each experiment. We also mention fish age in the figure caption and in the methods.

8) Results section 2, paragraph 2, Extended Data Figure 4: The authors mention that MYOD1 L122R RMS cells failed to differentiate in low serum conditions. It will help in appreciating the cell morphology and compare differentiated cells to the MYOD1 wildtype group if the authors include phase contrast microscopy images.

We have now included phase-contrast microscopy images. From these new studies, we observe large numbers of elongated, large cells with differentiated morphology in the $MYOD1^{WT}$ cells when grown under low serum, differentiation conditions. By contrast, $MYOD1^{L122R}$ expressing RMS do not have this same extent of differentiation after 7 days in culture. This data is now included in Extended Figure 4j.

9) There's a comment in Extended Data Figure S3 that should be deleted.

This comment has been removed.

10) Line 210-213: 'Upregulated genes were enriched for epithelial-to-mesenchymal transition (EMT) genes that have previously been shown to be enriched in FN cancer stem cells, while downregulated genes contained myogenesis and muscle differentiation genes'. This needs a citation.

We have now added the appropriate citation.

11) Growth assays or description of cell titer glo is missing in the methods section.

We have added description of these methods as requested.

12) Github link may still be set to private: https://github.com/rheinbaylab/Wei_MYOD_RMS

We apologize for this error. We had thought all data and github links were accessible in the first review of the manuscript. We have now made this link publicly available.

Reviewer #3:

Summary of work and impact: *"This is a well written and comprehensive study of the neomorphic transcriptional activity of mutant MYOD1 in rhabdomyosarcoma. While rare this is a clinically significant mutation that is associated with aggressive disease. I believe this manuscript will be a critical data set understanding the mechanism of the mutation. In addition the authors describe a potential therapeutic strategy targeting a downstream cell surface target of the mutation that has immediate clinical significance. Some thoughts to improve the presentation and the manuscript."*

1) Has the author tried PIK3CA co-mutation in the zebrafish. While there is some redundancy with RAS pathways these are not directly overlapping.

$MYOD1^{L122R}$ mutations co-occur ~70% with PIK3CA/RAS pathway activating mutations. RAS is commonly mutated, comprising 5 of 17 reported cases (Shern et al., JCO 2021). To date, our zebrafish modeling studies have only focused on assessing collaboration between activated RAS and $MYOD1^{L122R}$. Future work, beyond the scope of the current manuscript, could assess other known pathway regulators in modifying disease onset or phenotypes including activating mutations in PIK3CA as nicely noted by the reviewer.

2) *Can the authors comment on the penetrance of the co-mutation. Is there a reason why <50% get tumors but when sequentially passed they are 100% penetrant?*

As noted by the reviewer, zebrafish co-injected with $MYOD1^{L122R}$ and $KRAS^{G12D}$ develop RMS with a frequency of ~50%. These mosaic fish do not all express the transgenes to the same degree nor would all animals be expected to transform muscle into cancer. Yet, these mosaic fish developed RMS far more frequently than those expressing $KRAS^{G12D}$ alone or when co-expressed with wild-type MYOD1 (Figure 1d). When we harvested primary tumor cells and transplanted them into recipient fish at 20,000 cell/animal, 100% of fish engrafted with $MYOD^{L122R}$ + $KRAS^{G12D}$ expressing RMS developed tumors (n=4 tumors analyzed in Figure 1e). This reflects that these tumors were highly aggressive and contained sufficient numbers of tumor propagating cells to seed new tumors. By contrast, those that expressed $KRAS^{G12D}$ alone were far less aggressive and contained fewer overall numbers of tumor propagating cells, reflecting that only ~50% of animals developed transplantable disease. We have added additional text to clarify these results.

3) Fig2a. The Ruch2 construct seems leaky with significant HA expression in the non-induced state. Given the potency of the mutation would the authors not anticipate that this would be problematic in interpretation of downstream genes?

The differences in expression seemed to be related partly to differences in the confluence of cells at time of induction. We have repeated these experiments and replaced Figure 2a with a more representative example of our Western blot results.

4) In the RD cell line what happens to the endogenous WT MYOD1 expression when the mutation is present? Does the mutant feedback and diminish the endogenous expression?

In the dox-inducible RD model that expresses $MYOD1^{L122R}$, we do see a diminution of WT MYOD protein expression (see Figure 2b), suggesting that mutated MYOD1 might be altering binding and modulating the self-sustaining core-regulatory networks regulated in part by

MYOD1. This is an exciting finding for future studies and thank the reviewer for alerting us to this observation.

5) 4a and b. The "genes" label is a little confusing. Why do they just select 17 genes to present here? I assume they think these are the critical ones but how were they selected out of the bigger group? Are these from the leading edge genes of the GSEA? The Z-score of the heatmap is not super informative. It would be nice to get a sense of the actual expression of these genes rather than a comparison between mutant and non-mutant.

Many of the genes denoted in figure 4 were included based on their relevance to the predefined progenitor and differentiated muscle signatures. In fact, ALDH1A3, COL6A3, and COL3A1 were part of the leading-edge genes of the progenitor signature, while MYOG, ACTA1, DES, HES6, and ACTN2 were among the leading-edge genes of the differentiated muscle signature (refer to a_GSEA_Leading_Edge_Genes.xlsx).

We have now (1) only listed the cell-state associated genes in order of how they are presented in the heat map (highest to lowest FC) and (2) added colors for the genes associated with the progenitor signature (green) vs differentiated muscle signature (red) to make this clear. We have included the normalized gene expression values for all genes shown in Figure 4 in Source Table Figure 4b.xlsx.

6) Fig 4d. Its hard to tell in the single cell data from the plots but it appears that each of the MYOD1 mutant tumors might have at least a couple of distinct cluster while the WT tumors are more of a cloud. Are these transcriptional subclones or groups of cells dividing?

We do observe that some of the MYOD ^{L122R} tumors seem to contain molecularly defined TSNE clusters; yet these do not correlate with a specific cell cycle state (see below). Shown below is the scRNA-seq data generated from the PDX tumors and assessing transcriptional programs associated G1 – G2/M and S-phase.

As suggested by the reviewer, it is possible that there may be additional clonal heterogeneity in the MYOD1 ^{L122R} PDXs, yet statements about the possible clonal expansions and broad applicability to human SS-RMS will require larger numbers of samples and refined analysis.

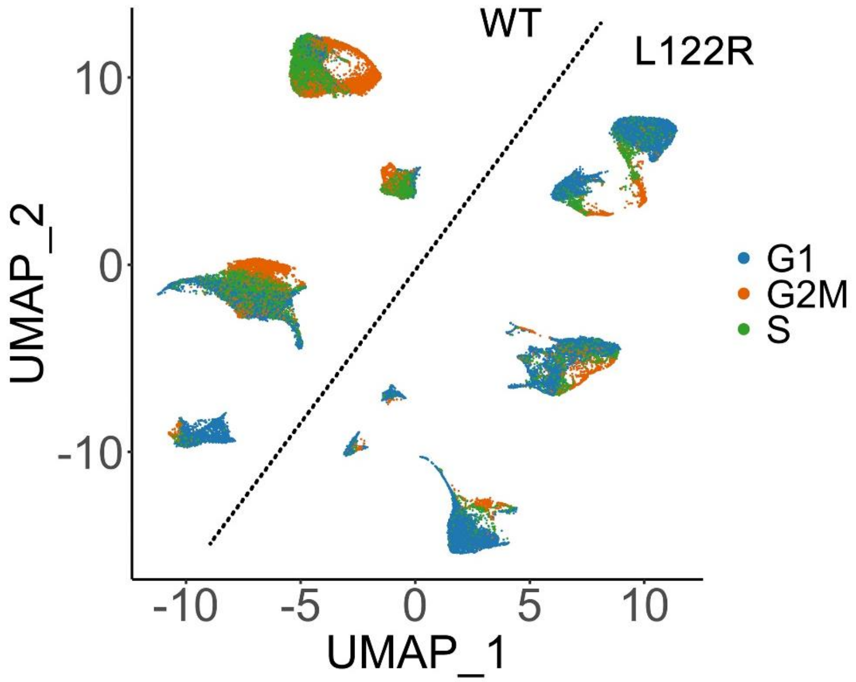

7) In Fig5a are the RD cells with FLAG constructs single cell cloned or is more heterogeneous construct. The methodology of the cell line construction in this paper should be robust and detailed as these reagents and the data coming from may be important for future investigations in the field.

We have revised the text to better define the methodology for cell line construction. These cells were obtained by bulk cell infection with lentiviral constructs that harbor flag-tagged or HA-tagged MYOD1 (see updated Methods section).

Specifically, for the generation of RD cells with dox-inducible FLAG-MYOD1, we utilized a construct from the Matthew Alexander Lab (Addgene #78330). This pInd20-FLAG-MYOD1 construct was then used to generate a mutant MYOD1 variant using site-directed mutagenesis. Plasmid was then isolated from a single clone and verified by sequencing, after which it was used to generate virus. Viruses were collected within 72 hours and used to infect RMS cells accordingly. Cells were virally-transduced and treated in bulk with Geneticin. Westerns were performed to validate overexpression or knockout in all experiments and were shown in figures.

8) Figure 5a. Can the authors include Chipseq using a MYOD1 antibody in the parental cell line? It is possible that some of the L122R "exclusive sites" have endogenous WT MYOD1 at these genomic locations. In parallel it would be nice to show ChIPseq of MYC in the same manner.

While we agree with the reviewer the proposed ChIPs would be highly valuable, unfortunately, our ChIPseq studies using antibodies directed against MYOD and MYC protein did not enrich and yielded poor quality results, prompting us to use a tagged strategy. Notably, Ruch2 does not express endogenous MYOD1. Yet to directly assess the intent of the question, we have completed a wide array of additional studies for this revision and find no cooperative binding of MYOD1 ^{L122R} with MYC. This is now supported by the following:

i) We do not observe a difference in the expression of cMYC in the $MYOD1^{L122R}$ models. Specifically, RNA sequencing and Western Blot analysis confirmed that cMYC transcription and protein expression are not differentially regulated in our $MYOD1^{L122R}$ models (now presented in a new Extended Data Figure 12).

ii) We assessed potential association of MYC and MYOD1/ $MYOD1^{L122R}$ using proximity ligation assay (PLA), a technique that enables highly specific in situ detection of protein–protein interactions or conformational changes by using pairs of antibodies conjugated to oligonucleotides that, when in close proximity (<40 nm), undergo ligation and rolling circle amplification, generating a localized fluorescent signal that can be quantitatively imaged. PLA confirmed MYOD1:MYOD1 homodimerization and cooperative binding of MYOD1:$MYOD1^{L122R}$; yet dimerization between either MYOD1 or $MYOD1^{L122R}$ with MYC was not observed. Control experiments confirmed MYC:MAX interactions while single antibody experiments showed no rolling circle amplification. These results suggest that MYC does not associate with MYOD1 or Mutant $MYOD1^{L122R}$.

(iii) We performed co-immunoprecipitation using HA-conjugated beads in HEK293T cells that expressed either HA-MYOD1 or HA-$MYOD1^{L122R}$ and FLAG-MYC followed by Western blot. In this experiment, we did not observe binding of MYOD1 or $MYOD1^{L122R}$ to MYC. As expected, our controls confirmed MYOD1:MYOD1 homodimerization and cooperative binding of MYOD1:$MYOD1^{L122R}$.

9) Fig 5e and 5f. It would be helpful to add RNAseq tracks of these genes and loci.

We have now added RNAseq tracks for these genes and loci. As expected, sites with $MYOD1^{L122R}$ binding have corresponding high transcript peaks at these locations.

10) It's a little surprising that the PDX trial of the ROR2 targeting ADC are not included in this work.

We have now added in vivo experiments in which we xenografted our RD dox-inducible models expressing either wild-type MYOD1 or $MYOD1^{L122R}$ into NSG mice, allowed the tumors to engraft until palpable and then treated the mice with an ADC targeting ROR2 (Ozuriftamab vedotin) weekly for four weeks. As expected, WT MYOD1 tumors were not affected by the drug treatment, reflecting lack of ROR2 target expression. By contrast, $MYOD1^{L122R}$ -expressing tumors regressed after treatment and did not grow back over the course of our studies. This work mirrors our *in vitro* findings presented in the first submission and provides exciting new directions for clinical application. Relevant data is now included in Figure 7h-j and Extended Figure 19f-h.

Finally, we have also now tested PDX spheres *ex vivo* and show that sphere growth is reduced in mutant MYOD1 tumors when treated with ADCs.
